# Characteristics of HIV seroconverters in the setting of universal test and treat: Results from the SEARCH trial in rural Uganda and Kenya

**DOI:** 10.1371/journal.pone.0243167

**Published:** 2021-02-05

**Authors:** Marilyn N. Nyabuti, Maya L. Petersen, Elizabeth A. Bukusi, Moses R. Kamya, Florence Mwangwa, Jane Kabami, Norton Sang, Edwin D. Charlebois, Laura B. Balzer, Joshua D. Schwab, Carol S. Camlin, Douglas Black, Tamara D. Clark, Gabriel Chamie, Diane V. Havlir, James Ayieko

**Affiliations:** 1 Kenya Medical Research Institute, Kisumu, Kenya; 2 Division of Biostatistics and Epidemiology, School of Public Health, University of California Berkeley, Berkeley, California, United States of America; 3 Infectious Diseases Research Collaboration, Kampala, Uganda, Makerere University College of Health Sciences, Kampala, Uganda; 4 Center for AIDS Prevention Studies, University of California San Francisco, San Francisco, California, United States of America; 5 Department of Biostatistics and Epidemiology, University of Massachusetts Amherst, Amherst, Massachusetts, United States of America; 6 Department of Obstetrics, Gynecology and Reproductive Sciences, University of California San Francisco, San Francisco, California, United States of America; 7 Division of HIV, Infectious Diseases and Global Medicine, University of California San Francisco, San Francisco, California, United States of America; International AIDS Vaccine Initiative, UNITED STATES

## Abstract

**Background:**

Additional progress towards HIV epidemic control requires understanding who remains at risk of HIV infection in the context of high uptake of universal testing and treatment (UTT). We sought to characterize seroconverters and risk factors in the SEARCH UTT trial (NCT01864603), which achieved high uptake of universal HIV testing and ART coverage in 32 communities of adults (≥15 years) in rural Uganda and Kenya.

**Methods:**

In a pooled cohort of 117,114 individuals with baseline HIV negative test results, we described those who seroconverted within 3 years, calculated gender-specific HIV incidence rates, evaluated adjusted risk ratios (aRR) for seroconversion using multivariable targeted maximum likelihood estimation, and assessed potential infection sources based on self-report.

**Results:**

Of 704 seroconverters, 63% were women. Young (15–24 years) men comprised a larger proportion of seroconverters in Western Uganda (18%) than Eastern Uganda (6%) or Kenya (10%). After adjustment for other risk factors, men who were mobile [≥1 month of prior year living outside community] (aRR:1.68; 95%CI:1.09,2.60) or who HIV tested at home vs. health fair (aRR:2.44; 95%CI:1.89,3.23) were more likely to seroconvert. Women who were aged ≤24 years (aRR:1.91; 95%CI:1.27,2.90), mobile (aRR:1.49; 95%CI:1.04,2.11), or reported a prior HIV test (aRR:1.34; 95%CI:1.06,1.70), or alcohol use (aRR:2.07; 95%CI:1.34,3.22) were more likely to seroconvert. Among survey responders (N = 607, 86%), suspected infection source was more likely for women than men to be ≥10 years older (28% versus 8%) or a spouse (51% vs. 31%) and less likely to be transactional sex (10% versus 16%).

**Conclusion:**

In the context of universal testing and treatment, additional strategies tailored to regional variability are needed to address HIV infection risks of young women, alcohol users, mobile populations, and those engaged in transactional sex to further reduce HIV incidence rates.

## Introduction

Universal test and treat (UTT) seeks to preserve health of persons living with HIV (PLWH) and to reduce new HIV infections through a strategy that deploys comprehensive population level HIV testing followed by HIV treatment (antiretroviral therapy, ART) for all PLWH. Three large UTT trials conducted in sub Saharan Africa demonstrated that population level viral suppression rates at or above the UNAIDS “90-90-90” threshold of 73% could be achieved within 3 years using a variety of HIV testing strategies including home-based testing and health fairs, coupled with linkage and universal ART eligibility [1–4]. UTT, as implemented in these studies, reduced but did not eliminate all new HIV infections. As public health leaders contemplate application of UTT on a sub-country level to achieve rapid high population levels of viral suppression and associated reductions in HIV incidence, it is important to both address the gaps in the HIV testing and treatment cascade of UTT as well as to identify populations still at risk for HIV infection [4–6]. Populations still acquiring HIV in the setting of UTT are logical candidates for other prevention measures such as pre-exposure prophylaxis (PrEP) [7].

The Sustainable East Africa Research in Community Health (SEARCH) trial was a community cluster-randomized UTT trial that was conducted in three regions of rural Uganda and Kenya from 2013 through 2017 (NCT01864603). As previously described [4], the SEARCH trial tested a multi-disease, streamlined care approach to reduce HIV incidence and improve community health. Both intervention and control arms in SEARCH included universal baseline HIV testing and referrals to HIV care. All persons in the intervention communities were ART eligible from study start; eligibility for ART in control communities was CD4-restricted according to country guidelines. However, one year into the study, rapid implementation of expanded ART eligibility occurred in the control arm. At the end of 3 years, there was a high cumulative testing coverage within both study arms (98% intervention and 96% control); the intervention arm achieved higher ART coverage (87% intervention versus 78% control) and population level viral suppression (79% intervention versus 68% control) [4]. Although over 3 years HIV incidence decreased by 32% in the intervention arm, HIV incidence did not differ between the study arms, a finding attributed to the comprehensive baseline HIV testing and nearly equivalent ART eligibility in the control arm. To provide insights into persons still acquiring HIV infection in the context of UTT where high levels of population level viral suppression were achieved, we sought to characterize the HIV seroconverters in the SEARCH study, evaluate gender-specific risk factors, and describe their self-reported probable sources of infection.

## Methods

### Study setting and population

Details of the SEARCH study procedures have been described previously [4]. In brief, we conducted a rapid census to capture demographics for 355,848 persons (adults and children) in the study communities at the start of the study. At baseline in both intervention and control communities, we delivered universal HIV testing through health fairs and home visits. Members of the intervention communities were all eligible for ART and were offered care in using a multi-disease, streamlined care model at government clinics, while participants in control communities received care as per in-country guidelines that rapidly expanded from CD4-specific to treating all. The primary study endpoint was measured at 3 years in an HIV incidence cohort, consisting of persons who at baseline were ≥ 15 years, had spent at least 6 months of the last year in the community, and were documented to be HIV-uninfected. HIV seroconverters were defined as members of this cohort who tested HIV positive at 3 years, and had HIV status confirmation by Bio-RaD Geenius HIV 1/2 assay and Western blot testing. HIV seroconverters were counseled and offered ART if they had not yet previously started or if they had previously started therapy and discontinued but were willing to re-engage in HIV care and treatment.

### HIV seroconversion surveys

A survey exploring potential sources of infection was administered by trained staff to all HIV seroconverters who could be traced and consented to participate within 6 months following the 3-year HIV seroconversion endpoint measurement. The survey explored participants’ thoughts on potential sources of infection. Participants were asked to describe characteristics of suspected source of infection including their age difference (< or ≥10 years), gender, relationship to seroconverter (spouse or not), residence (whether from within the seroconverter’s community or from outside the community), and whether sexual contact was in exchange for money or other goods or services (transactional sex).

### Measures

Participants’ demographics, potential risk factors, and HIV testing were collected on study-designed forms, which were then entered and stored in a central database. Birthdate, marital status, occupation, mobility, contraceptive use, and alcohol use were collected at baseline. Marital status was classified as single, married (monogamous or polygamous; a man married to more than one wife or a woman married to a man with more than one wife), divorced and widowed. Occupations were broadly categorized as formal (student, teacher, government worker, health worker, military worker, factory worker), high-risk informal (fisherman, bar owner, bar worker, truck/taxi/bike/boat driver or tourism), low-risk informal (farmer, shopkeeper, hotel worker, market vendor, household worker, or construction worker), no job, or other. Mobile residents were defined as those living >1 month of the prior year away from the community. Additionally, a wealth index (quintiles) was created using a principle component analysis of a household survey of livestock and household items [8]. Finally, at baseline multi-disease testing, participants self-reported whether they had previously tested for HIV.

### Statistical analyses

To describe who seroconverted to HIV positive within 3 years, we reported the number and proportion of seroconverters by demographic factors as well as prior and baseline HIV testing. Descriptions were provided overall and stratified by gender and region. Then using data from all members of the HIV incidence cohort, we calculated gender-specific incidence rates (IR) per 100 person-years overall and for each predictor separately and with exact Poisson confidence intervals (CI). Next, to identify gender-specific risk factors for HIV seroconversion, we used targeted maximum likelihood estimation (TMLE) [9] to estimate adjusted relative risks (aRR) for each gender separately. Our pre-specified risk factors, selected based on literature, were age group (<25 years or 25+ years), marital status, occupation, wealth index, contraceptive use, alcohol use, mobility, self-reported prior HIV testing before baseline, and the location of the baseline HIV test. These analyses additionally controlled for incomplete follow-up, region, and randomization arm. Unadjusted risk ratios were reported as secondary analysis. Additionally, based on the seroconversion surveys, we grouped together suspected sources of infection, which were summarized as proportions and stratified by gender. All analyses conducted pooled over trial arms and were pre-specified for the SEARCH trial. Additional details are available in the Supplementary Materials.

### Ethical approval

The Kenya Medical Research Institute Ethical Review Committee (Kenya), Ugandan National Council on Science and Technology (Uganda), Makerere University School of Medicine Research and Ethics Committee (Uganda), and University of California San Francisco Committee on Human Research (USA) approved the study. All participants in the SEARCH Study [10] provided verbal consent. Those who were reached and were willing to participate in the seroconversion survey provided a written consent in their preferred language.

## Results

### HIV incidence and descriptions of HIV seroconverters

Within the SEARCH trial, the HIV incidence cohort consisted of 117,114 participants of whom 1813 (1.5%) died, 8502 (7.3%) out-migrated, and 11,716 (10%) were not tested for HIV at year 3. Of the remaining 95,083 (81.2%) of participants, a total of 704 seroconversions were recorded (360 intervention and 344 control). As previously reported, the 3-year cumulative HIV incidence was 0.77% within the intervention arm and 0.81% within the control arm (RR 0.95; 95% CI 0.77,1.17); the corresponding HIV incidence rates were 0.25 per 100 person-years (95%CI 0.18,0.33) and 0.27 per 100 person-years (95%CI 0.20,0.33), respectively [4].

Of the 704 seroconverters, 443/704 (63%) were female and 330/704 (47%) occurred in Kenya (Table 1). Full characteristics of the incidence cohort reported in S1 Table. Participants aged 15–34 years accounted for 499/704 (73%) of all the seroconversions. Sixty two percent (434/704) of the seroconverters with marital status reported were married and 25% (174/704) were single. Among the 467 seroconverters with linked testing data on their known partners, 18% (85/467) were in serodiscordant relationships and 21% (97/467) had partners who did not test for HIV at baseline. Farming was the most common occupation (350/704 seroconverters with known occupation; 50%). Alcohol use was reported among 123/631 (19%) of the seroconverters who responded to the question on alcohol use. Only 477/704 (68%) of seroconverters self-reported that they had taken an HIV test prior to baseline testing.

**Table 1:** Descriptive characteristics in percent (numerator/denominator) of 704 seroconverters identified in the SEARCH test-and-treat trial in 32 rural Uganda and Kenyan communities, overall and by gender.

Characteristic		All (704)	Men (261)	Women (443)
Region	W. Uganda	38% (265/704)	44% (115/261)	34% (150/443)
	E. Uganda	15% (109/704)	15% (40/261)	16% (69/443)
	Kenya	47% (330/704)	41% (106/261)	51% (224/443)
Age	15–24 years	37% (263/704)	33% (87/261)	40% (176/443)
	25–34 years	34% (236/704)	38% (98/261)	31% (138/443)
	35–44 years	18% (126/704)	19% (49/261)	17% (77/443)
	45–54 years	7% (46/704)	5% (14/261)	7% (32/443)
	55+ years	5% (33/704)	5% (13/261)	5% (20/443)
Marital Status	Single	25% (174/703)	32% (84/261)	20% (90/442)
	Married	62% (434/703)	62% (163/261)	61% (271/442)
	Widowed	6% (45/703)	2% (4/261)	9% (41/442)
	Divorced or separated	7% (50/703)	4% (10/261)	9% (40/442)
Among married	Polygamous marriage	22% (97/434)	15% (24/163)	27% (73/271)
Occupation	Farmer	50% (350/703)	45% (117/261)	53% (233/442)
	Fishing/Fishmonger	7% (48/703)	9% (24/261)	5% (24/442)
	Student	11% (77/703)	8% (22/261)	12% (55/442)
	Hotel/Restaurant	1% (10/703)	0% (1/261)	2% (9/442)
	Transport	2% (15/703)	5% (14/261)	0% (1/442)
Wealth index	First, least wealth	21% (145/702)	18% (47/261)	22% (98/441)
	Second	18% (127/702)	20% (52/261)	17% (75/441)
	Third	20% (142/702)	18% (47/261)	22% (95/441)
	Fourth	20% (142/702)	25% (65/261)	17% (77/441)
	Fifth, most wealth	21% (146/702)	19% (50/261)	22% (96/441)
Contraception	Report use	31% (199/632)	24% (60/252)	37% (139/380)
Alcohol	Report use	19% (123/631)	33% (79/241)	11% (44/390)
Mobility	1+ month away	12% (84/704)	16% (41/261)	10% (43/443)
Testing	Report prior test	68% (477/704)	59% (155/261)	73% (322/443)
	Test at heath fair	74% (518/704)	56% (146/261)	84% (372/443)
Relation to household head	Self	38% (268/703)	54% (142/261)	29% (126/442)
	Spouse	29% (202/703)	9% (23/261)	40% (179/442)
	Child	24% (167/703)	30% (77/261)	20% (90/442)
	Parent	0% (2/703)	0% (0/261)	0% (2/442)
Partner	Discordant partner	18% (85/467)	18% (29/162)	18% (56/305)
	Partner did not test	21% (97/467)	16% (26/162)	23% (71/305)

**Fig 1:**
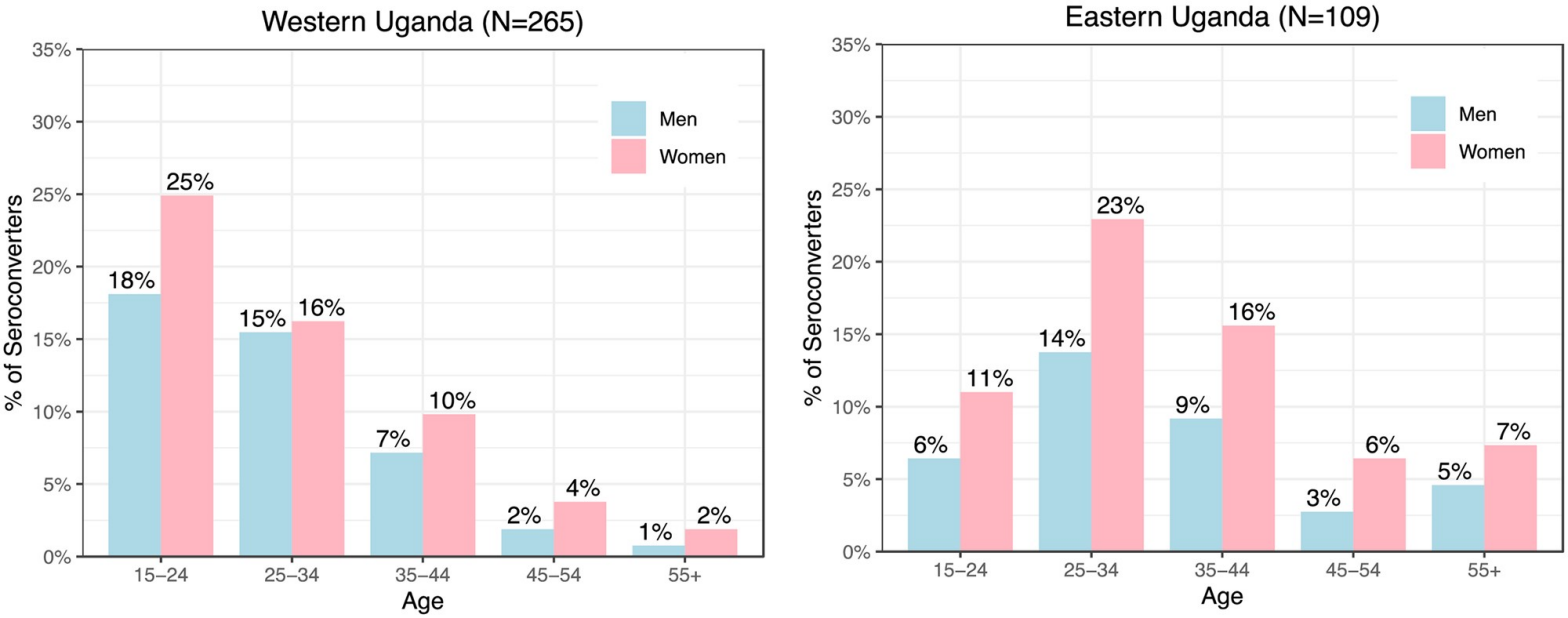
Seroconverters by age group and gender in the SEARCH test-and-treat trial in 3 regions in rural East Africa: Western Uganda, Eastern Uganda, and Kenya.

Across the three regions, women comprised the majority of seroconversions: 150/265 (57%) in Western Uganda, 69/109 (63%) in Eastern Uganda, and 224/330 (68%) in Kenya (S2 Table). Western Uganda (18%) had a higher proportion of male seroconverters below the age of 24 years as compared to Kenya (10%) and East Uganda (6%) (Fig 1).

The overall HIV incidence rates, in seroconversions per 100 person-years, were 0.22 (95%CI 0.20,0.25) for men and 0.29 (95%CI 0.26,0.32) for women (Table 2). Predictor-specific HIV incidence rates also varied by gender. Among men, incidence rates were highest among those who were divorced or separated (IR 0.48; 95%CI 0.23,0.88), in high-risk informal occupations (IR 0.48; 95%CI 0.34,0.66), without jobs (IR 0.35; 95%CI 0.20,0.58), were mobile (IR 0.33; 95%CI 0.24,0.45), and tested at home (IR 0.45; 95%CI 0.37,0.54). Among women, the highest incidence rates were among those less than 25 years old (IR 0.40; 95%CI 0.34,0.46), divorced or separated (IR 0.66; 95%CI 0.47,0.89), in high-risk informal occupations (IR 0.74; 95%CI 0.48,1.10), without jobs (IR 0.40; 95%CI 0.27,0.56), used contraceptives (IR 0.42; 95%CI 0.35,0.49), consumed alcohol (IR 0.40; 95%CI 0.29,0.53), and were mobile (IR 0.42; 95%CI 0.31,0.57).

**Table 2:** Gender-specific HIV incidence rates per 100 person-years (95% confidence intervals) in SEARCH trial (2013–2017) in rural Kenya and Uganda.

Seroconversion Predictor	Level	Male 0.22 (0.2,0.25)	Female 0.29 (0.26,0.32)
Age	25+ years old	0.23 (0.2,0.27)	0.25 (0.22,0.28)
	<25 years old	0.21 (0.17,0.26)	0.40 (0.34,0.46)
Marital Status	Divorced or separated	0.48 (0.23,0.88)	0.66 (0.47,0.89)
	Single	0.22 (0.19,0.26)	0.27 (0.24,0.3)
	Married	0.21 (0.17,0.26)	0.38 (0.3,0.47)
	Widowed	0.28 (0.08,0.72)	0.19 (0.14,0.26)
Occupation	Formal	0.1 (0.06,0.14)	0.27 (0.2,0.34)
	High-risk Informal	0.48 (0.34,0.66)	0.74 (0.48,1.1)
	Low-risk Informal	0.22 (0.19,0.26)	0.27 (0.24,0.3)
	Jobless	0.35 (0.2,0.58)	0.4 (0.27,0.56)
	Other	0.41 (0.28,0.58)	0.33 (0.18,0.56)
Wealth index	First, least wealth	0.28 (0.2,0.37)	0.39 (0.32,0.48)
	Second	0.25 (0.19,0.33)	0.27 (0.21,0.34)
	Third	0.2 (0.14,0.26)	0.3 (0.24,0.37)
	Fourth	0.24 (0.19,0.31)	0.23 (0.18,0.28)
	Fifth, most wealth	0.17 (0.13,0.22)	0.28 (0.23,0.34)
Contraceptive use	No	0.27 (0.23,0.31)	0.29 (0.25,0.32)
	Yes	0.23 (0.18,0.3)	0.42 (0.35,0.49)
	Declined to respond	0.05 (0.02,0.09)	0.18 (0.14,0.23)
Alcohol use	No	0.2 (0.17,0.23)	0.27 (0.24,0.3)
	Yes	0.27 (0.21,0.33)	0.4 (0.29,0.53)
	Declined to respond	0.28 (0.17,0.43)	0.45 (0.34,0.59)
Mobile	No	0.21 (0.18,0.24)	0.28 (0.25,0.31)
	Yes	0.33 (0.24,0.45)	0.42 (0.31,0.57)
Prior HIV test	No	0.45 (0.37,0.54)	0.36 (0.28,0.45)
	Yes	0.16 (0.13,0.19)	0.28 (0.25,0.31)
Baseline testing	Home-based testing	0.23 (0.2,0.27)	0.25 (0.22,0.28)
	Health fair	0.21 (0.17,0.26)	0.4 (0.34,0.46)

### Gender-specific risk factors for seroconversions

**Fig 2:**
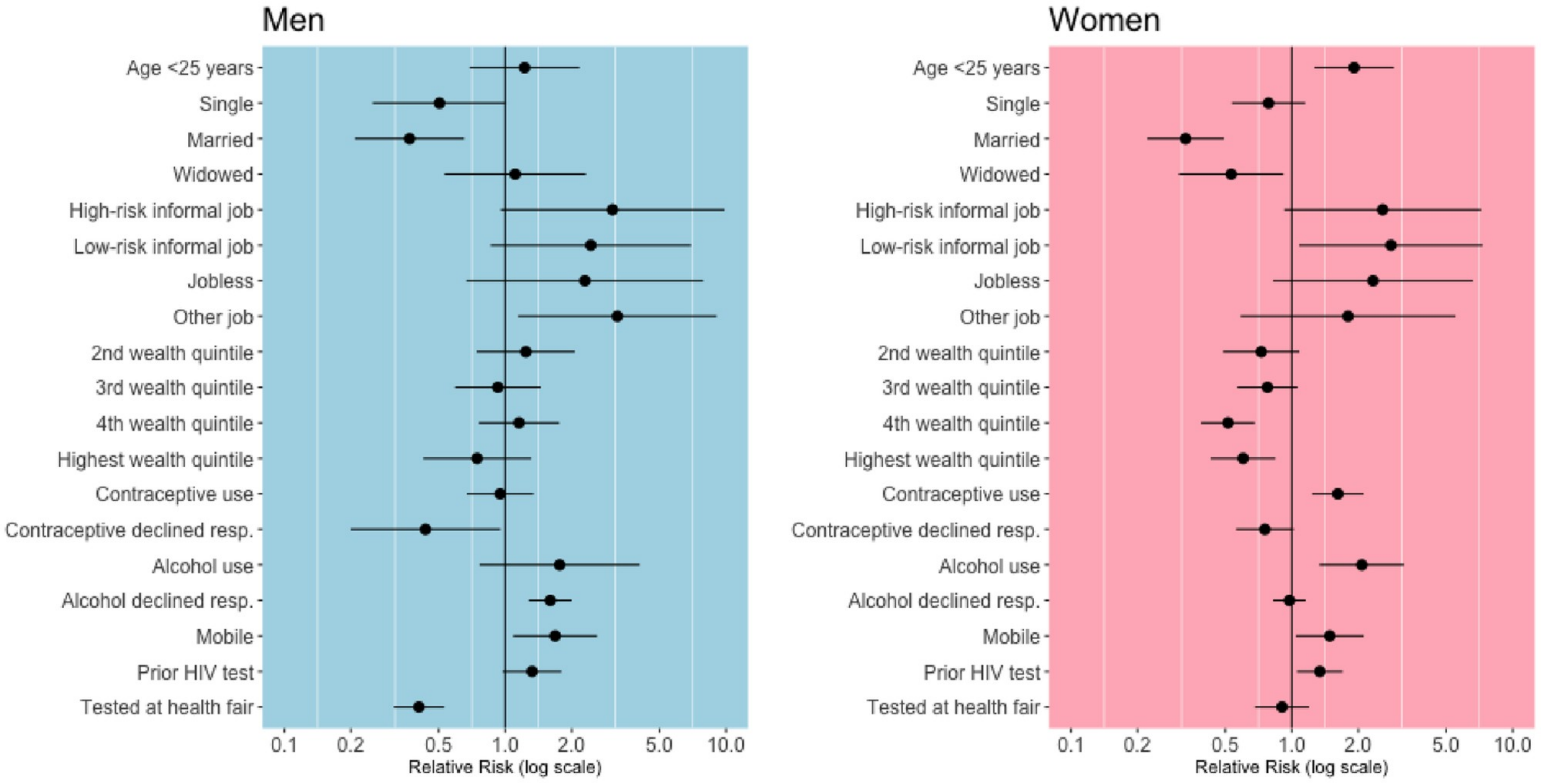
Adjusted risk ratios for HIV seroconversion stratified by gender. Analyses conducted among members of the HIV incidence cohort using targeted maximum likelihood estimation, controlling for incomplete follow-up and additionally adjusting for region and randomization arm *. * Reference categories were age 25+ years, separated or divorced, formal occupation, lowest wealth quintile, no contraceptive use, no alcohol use, non-mobile, no prior HIV test reported, and home-based testing.

In multivariable analyses, risk factors for seroconversion differed by gender (Fig 2; S3 Table). Analyses pooling over gender revealed that men as compared to women were at significantly lower risk of seroconversion (aRR 0.64; 95%CI 0.50,0.83; S2 Fig). Among women, factors significantly associated with increased risk of seroconversion included younger age (aRR 1.91; 95%CI 1.27,2.90), working in a low-risk informal job (aRR 2.81; 95%CI 1.08,7.32), contraceptive use (aRR 1.62; 95%CI 1.24,2.11), alcohol use (aRR 2.07; 95%CI 1.34,3.22), mobility (aRR 1.49; 95%CI 1.04,2.11), and self-reported prior HIV testing (aRR 1.34; 95%CI 1.06,1.70), while factors significantly associated with decreased risk included being married (aRR 0.33; 95%CI 0.22,0.49), being widowed (aRR 0.53; 95%CI 0.31,0.92), and higher wealth (aRR 0.60; 95%CI 0.43,0.84). Among males, declining to discuss alcohol use (aRR 1.60; 95%CI 1.28,2.0) and being mobile (aRR 1.68; 95% CI 1.09,2.60) were significantly associated with increased risk of seroconversion, while being married (aRR 0.37; 95% CI 0.21,0.65) and having tested at the baseline health fair (aRR 0.41; 95% CI 0.31,0.53) were significantly associated with reduced risk of seroconversion. Unadjusted risk ratios yielded similar results and are provided in the S1 Fig. Results restricting to the intervention arm, where UTT was implemented at baseline, were also similar (data not shown).

### Suspected sources of infection

Of the 704 seroconverters, 607 (86.2%) were reached and consented to participate in the survey exploring potential sources of infection. The remaining 97 (13.8%) either could not be traced (86(12.2%)) or declined to take part in the survey (11(1.6%)). Women were more likely to report their spouse as the source of infection (51% versus 31%), while men were more likely to report their suspected source of infection as transactional sex (16% versus 10%; Table 3). Men were also more likely to report their suspected source to be a short-term casual relationship (59% versus 30%; Table 4). Women were more likely than men to report a greater than 10-year age difference with their suspected source of infection (28% versus 8%). None of the participants reported a men-who-have-sex-with-men (MSM) relation as the suspected source of infection in the survey. Seventy-seven percent (470/607) of the seroconverters were able to identify the residence of their suspected sources of infection. Out of these, 133/470 (28.3%) reported that their suspected source of infection was from outside the participants’ communities: 66/247 (26.7%) in the intervention arm versus 67/223 (30.0%) in the control arm.

**Table 3:** Results in % (numerator/denominator) for the seroconverter survey for suspected sources and modes of HIV infection among the 607 participants, overall and by gender.

	All (607)	Male (219)	Female (388)
**1) Suspected source of infection was their spouse**			
Known	85% (516/607)	83% (182/219)	86% (334/388)
Yes	44% (228/516)	31% (56/182)	51% (172/334)
No	56% (288/516)	69% (126/182)	49% (162/334)
**2) Suspected source of infection was transactional sex**			
Known	85% (516/607)	83% (182/219)	86% (334/388)
Yes	12% (62/516)	16% (30/182)	10% (32/334)
No	88% (454/516)	84% (152/182)	90% (302/334)
**3) Age difference with suspected source of infection**			
Known	77% (467/607)	75% (165/219)	78% (302/388)
< 10 Years Older	79% (368/467)	92% (152/165)	72% (216/302)
≥10 Years Older	21% (99/467)	8% (13/165)	28% (86/302)
**4) Residence of suspected source of infection**			
Known	77% (470/607)	74% (161/219)	80% (309/388)
Inside	72% (337/470)	65% (105/161)	75% (232/309)
Outside	28% (133/470)	35% (56/161)	25% (77/309)

**Table 4:** Overall and by gender, the reported suspected source of infection among the 521 seroconverters who participated in the survey and responded to this question.

	Overall		Males		Female	
*Reported suspected source*	*n*	*%*	*n*	*%*	*n*	*%*
Infected spouse in marriage	228	43.8	56	31.1	172	50.4
Short term casual sexual relationship	208	39.8	107	59.4	101	29.6
Long term relationship	30	5.8	6	3.3	24	7.0
Cultural wife inheritance practice*	27	5.2	3	1.7	24	7.0
Occupational exposure**	9	1.7	1	0.6	8	2.3
Rape/non-consensual sex	5	1.0	1	0.6	4	1.2
Does not know source	6	1.1	2	1.1	4	1.2
Refused to reveal	8	1.5	4	2.2	4	1.2
**Total**	**521**	**100**	**180**	**100**	**341**	**100**

*Wife inheritance- cultural cleansing practice that involves sexual intercourse after the death of husband.

**Occupational exposure- includes caring for a sick relative, injuries with infected items such as razor blades, needles etc.

## Discussion

In the context of a successful UTT implementation in rural Kenya and Uganda, clearly identifiable subgroups of persons continue to experience high risks of HIV seroconversion. Analyses of ~100,000 persons from the SEARCH study indicate particular increased risks for young women, alcohol users, and mobile populations. Interviews of seroconverters further indicate increased risks for those women whose partners are substantially older and those men who engaged in transactional sex. The annual HIV incidence declined by 30% within this high HIV prevalence setting in the context of a population-level HIV test-and-treat intervention using a multi-disease, streamlined care approach [4]. However, we still observed a significant number of new HIV infections. This demonstrates that UTT with population-level viral suppression exceeding 73% may not by itself be the means to end the epidemic and, instead, might serve to stabilize and slow the epidemic as other interventions to achieve full control are sought. Age continues to exert a powerful effect on HIV infection risk, with youth comprising the majority of the new HIV infections observed consistent with findings from other studies [11, 12]. Of note, however, a fair proportion of new infections were observed among older individuals (>50 years), suggesting that while attention is given to the youth and adolescents, prevention interventions should incorporate the older age groups that have previously not received as much attention in the HIV control response.

Our findings demonstrate geographic heterogeneity in the pattern of new HIV infections, with Western Uganda recording more infections among young men (aged <25 years) compared to East Uganda and Kenya. This finding correlates to studies in similar settings that have demonstrated geographical variation of HIV risk factors [13, 14]. We speculate that age-gender-specific differences in socio-cultural practices between the two regions, including risk-enhancing behaviors such as alcohol use, may be responsible for the observed difference. This phenomenon has been demonstrated in past studies [15, 16]. In addition, the impact of the UTT approach may not be uniform across populations, and this may be responsible for variations of incidence across different groups. For example, a study in eSwatini showed different gains of ART scale up by gender; greater reduction in HIV incidence in males and more reduction in mortality among females within the same period of time [17]. This finding points to the need for varied preventive interventions tailored to regional differences that address the unique risk factors within different groups.

Women recorded higher numbers of new HIV infections compared to their male counterparts despite a near similar high gender-specific rate of viral suppression [4]. Within rural East Africa, HIV transmission to women is primarily through heterosexual transmission [18]. Biological factors that cause mucosal damage of the female genital tract such as bacterial vaginosis (BV) and endemic herpes simplex virus (HSV) that is estimated to affect about 50% of the women in Southern and Eastern Africa are significant contributors to this difference in HIV incidences [19, 20]. Additionally, behavioral factors, mostly driven by poverty [21], such as engagement in age-disparate sexual relationships that are characterized with hegemony and significant power differences [22–24], use of alcohol that results in impaired decision-making, disinhibition and engagement in risky sexual encounters [25–27], put women at increased risk for HIV acquisition. These may be responsible for the gender difference observed.

Within the sub-Saharan African setting, access to maternal and child health services provides an avenue for women to have more frequent and consistent contact with the healthcare system. This creates an opportunity for earlier HIV diagnoses, initiation of treatment and consequently better health outcomes among women compared to their male counterparts. Repeat HIV testing and use of contraceptives are some of the indicators of awareness, contact with the healthcare system and possibly good health seeking behavior [28, 29]. However, our findings indicate that these were important indicators of higher risk of seroconversion among women. One plausible explanation for this would be that repeat HIV testing and contraceptive use might be pointers of high sexual activity that may be of a high risk nature. Past studies in other settings have suggested that HIV repeat testers were more likely to engage in risky sexual behaviors and would continue to engage in risky encounters in between HIV tests [29, 30]. Also, this might be a pointer to the existence of other underlying risk factors such as behavioral characteristics within this population that might not be fully addressed during their interactions with the healthcare system. Thus, there is need for targeted holistic prevention interventions at the point of HIV testing and uptake of contraceptive services which may be markers of high risk behavior.

The majority of the seroconverters in this study were married. Of note is that the putative sources of infection within this setting varied significantly by gender. While women were more likely to attribute the new HIV infections to their spouses, most men attributed the new infections to short term casual relationships with other extra-marital sexual partners as well as transactional sex. Additionally, cultural practices such as wife inheritance and assigned gender roles (men as providers and women as nurturers and caregivers) are still significant drivers of HIV in rural East Africa [26]. In the quest to meet their roles as providers, men tend to move from one point to another and spend significant time away from their spouses [31, 32]. During these periods, men may engage in casual relationships for companionship and/or transactional sex that may expose them to the risk of infection [31, 33]. However, a study conducted in South Africa showed that the direction of infection is not always from the mobile male spouse returning home to the wife, but could also be from the wife to the mobile spouse [34]. This highlights the need for preventive interventions that not only target the mobile populations but also their non-migrant spouses. Our survey further revealed that women felt that their role as caregivers and nurturers continues to expose them to the risk of infection by caring for sick relatives without protection. Although the risk from such exposures is minimal, within regions of high HIV prevalence, there is need for interventions that equip caregivers with the knowledge of how best to protect themselves.

No respondent reported an MSM relationship as their putative source of infection. This may have been a result of the fact that unlike in most developed countries where homosexuality is increasingly getting embraced and protected by the law, in sub Saharan Africa homosexuality is characterized by numerous complexities such as unfavorable social settings, punitive legal frameworks and unresponsive healthcare systems [35]. Individuals in this demographic group may feel unsafe to reveal their involvement in MSM relationships.

Our analysis was not without limitations. First, potential risk factors measured on the HIV incidence cohort were largely limited to demographic predictors and did not include sexual behaviors or history of sexually transmitted infections. Second, our findings from the sources of infection survey were subject to social desirability bias due to self-report. We attempted to mitigate this bias in the following ways: 1) staff were trained on applying interviewing techniques to minimize fear of being judged; 2) participants were assured that the information provided would not be linked to them, but was to help in understanding how to deal with controlling the new infections; and, 3) surveys were conducted by clinicians who had built close relationships with the participants as their patients and were willing to share personal details even during other clinic visits. Third, there might have been a significant time lapse between infection and diagnosis and thus inaccuracy in the reported sources of infection (recall bias), and some persons may not have known their source of infection. Similar future studies should employ phylogenetic analysis to confirm the sources of transmissions as well as employ study designs that would minimize the time gap between infection and diagnosis.

## Conclusion

Scale-up of UTT has gone a long way in slowing down the HIV epidemic in rural East Africa by reducing the number of new HIV infections. However, within this setting, some demographic groups such as young girls, alcohol users, mobile populations, men who engage in transactional sex as well as women in intergenerational sexual relationships continue to record high HIV incidence rates. Consequently, in order to achieve control of the HIV epidemic, there is need for expansion of existing preventive interventions like PrEP and development of other targeted prevention interventions that are tailored not only to the unique needs of these populations but also to their contextual regional differences.

## Supporting information

S1 File SEARCH protocol version 6.0. (PDF)

S2 File SEARCH statistical analysis plan. (PDF)

S3 File SEARCH trial additional analytic details. (DOCX)

S1 Table Description of the HIV incidence cohort in the SEARCH trial. (DOCX)

S2 Table Description of HIV seroconverters identified in the SEARCH trial. (DOCX)

S3 Table Adjusted relative risks for SEARCH trial HIV seroconversion by gender. (DOCX)

S1 Fig Unadjusted relative risks for HIV seroconversion in SEARCH trial by gender. (DOCX)

S2 Fig Adjusted risk ratios for HIV seroconversion in SEARCH trial pooling over gender. (DOCX)
