# Supplementary material for: Characteristics of HIV seroconverters in the setting of universal test and treat: Results from the SEARCH trial in rural Uganda and Kenya
Source: PLoS One. 2021 Feb 5;16(2):e0243167. doi: 10.1371/journal.pone.0243167 (PMC7864429; doi:10.1371/journal.pone.0243167)
Supplement: S1 File — (PDF) [file pone.0243167.s001.pdf]

Sustainable East Africa Research in Community Health (SEARCH)

A Study of the:

Makerere University – University of California, San Francisco (MU-UCSF)
Research Collaboration

Protocol version: 6.0, October 1, 2015

Protocol Chairs:

Diane V. Havlir, MD
University of California, San Francisco, USA

Moses Kamya, MBChB, MPH, PhD
Makerere University
Kampala, Uganda

Maya L. Petersen, MD, PhD
University of California, Berkeley

Protocol Vice-Chair:

Edwin Charlebois, MPH, PhD
University of California, San Francisco, USA

Study supporters and sponsors: the Kenya Medical Research Institute (KEMRI); Infectious Diseases Research Collaboration (IDRC); the World Bank; National Institute of Allergy and Infectious Diseases (NIAID); PEPFAR; and Gilead Sciences, Inc.

TABLE OF CONTENTS

PROTOCOL TEAM ROSTER: 5
PROTOCOL SYNOPSIS 8
GLOSSARY 10
1.0 STUDY CONTEXT 11
1.1 Background 11
1.2 Rationale 12
1.3 Antiretroviral therapy 13
2.0 STUDY OBJECTIVES 16
2.1 Primary Study Objective 16
2.2 Secondary Objectives – Health 16
2.3 Secondary Objectives – Economic and Education Outcomes 17
2.4 Secondary Objectives – Cost and Cost-Effectiveness 17
3.0 STUDY DESIGN 18
4.0 STUDY POPULATION 19
4.1 Community Level Inclusion Criteria 19
4.2 Individual Level Inclusion Criteria 19
4.3 Community Level Exclusion Criteria 19
5.0 STUDY INTERVENTION 20
5.1 Antiretroviral Therapy in a Streamlined Care Delivery 20
6.0 COMMUNITY LEVEL STUDY EVALUATIONS 24
6.1 Baseline Household Community Level Census 24
6.2 Community Health Campaign (CHC) 24
6.3 Mortality and New Births, and HIV and TB Disease Surveillance 30
6.4 Feedback of CHC Process and Clinical Data 31
7.0 CLINCAL AND LAB EVALUATIONS IN ART INTERVENTION ARM 33
7.1 Recruitment 33
7.2 Selection of Participants in Intervention Arm 33
7.3 Informed Consent and Enrollment 33
7.4 Schedule of Evaluations 34
7.5 Timing of Evaluations 34
7.6 Definitions of Evaluations 35

8.0 ART TOXICITY GRADING AND MANAGEMENT FOR INTERVENTION ARM 37
8.1 Toxicity Screening 37
8.2 Management of Laboratory Toxicities 37
8.3 Management of Specific Clinical Syndromes 37
9.0 ECONOMIC AND EDUCATION EVALUATIONS 40
9.1 Overview 40
9.2 Recruitment and Enrollment of Study Participants 40
9.3 Procedures 40
10.0 HEALTH CARE COSTING EVALUATIONS 42
10.1 Overview 42
10.2 Recruitment and Enrollment of Study Participants 42
10.3 Procedures 42
11. STATISTICAL PLAN 45
11.1 Overview of Study Design 45
11.2 Primary Outcome Measurement 47
11.3 Randomization of Community Pairs 49
11.4 Primary Analyses 50
11.5 Secondary Analyses 54
12. DATA COLLECTION AND MONITORING 60
12.1 Data and Safety Monitoring Plan 60
12.2 Baseline Household Community Level Census 62
12.3 Community Health Campaign 63
12.4 ART Intervention 64
12.5 Grade 3 and 4 Adverse Event and Serious Adverse Event Monitoring 64
12.6 Household Socio-Economic Survey 65
12.7 Health Care Costing Evaluations 66
12.8 Data Security and Integrity 66
13. HUMAN SUBJECTS 67
13.1 Ethical Considerations 67
13.2 Institutional Review Board (IRB) and Informed Consent 67
14. PUBLICATION OF RESEARCH FINDINGS 68
REFERENCES 69
Appendix A Guidelines for Adverse Event Grading – DAIDS Toxicity Table for Adults and Children 72

Appendix B Table of Study and Consent and Commitment Procedures 76
Appendix C Uganda Antiretroviral Therapy Guidelines, in place until 2014..........................78
Appendix D Kenya Antiretroviral Therapy Guidelines..80
Appendix E Sample Pediatric ART Dosing Chart..82

PROTOCOL TEAM ROSTER:

Co-Chair:

Diane Havlir, MD
Chief, HIV/AIDS Division
San Francisco General Hospital
University of California, San Francisco
UCSF Box 0874
San Francisco, CA 94143-0874, USA
Phone: (415) 476-4082, x424
Fax: (415) 476-6953
Email: diane.havlir@ucsf.edu

Co-Chair:

Moses Kamya, MBChB, MMed, MPH, PhD
Makerere University
Plot 4B, Kololo Hill Drive
P.O. Box 7972
Kampala, Uganda
Phone: +256-414-533200
Fax: +256-414-540524
Email: mkamya@infocom.co.ug

Co-Chair and Protocol Statistician:

Maya L. Petersen, MD, PhD
Assistant Professor of Biostatistics and Epidemiology
School of Public Health
University of California, Berkeley
Berkeley, CA 94720-7358, USA
Phone (510) 642-0563
Email: mayaliv@berkeley.edu

Vice-Chair:

Edwin Charlebois, MPH, PhD
Professor of Medicine
University of California, San Francisco
Co-Director of Prevention, AIDS Policy Research Center, Center for AIDS Prevention Studies, AIDS Research Institute
550 16th Street, 3rd Floor
San Francisco, CA 94143, USA
Phone: (415) 476-6241
Fax: (415) 476-5348
Email: Edwin.Charlebois@ucsf.edu

Investigator:

Elizabeth Bukusi, MBChB, M.Med, MPH, PhD
Chief Research Officer and Deputy Director
Kenya Medical Research Institute
Co-Principal Investigator, FACES
Box 54840
Nairobi, Kenya
Phone: +254 733617503
Fax: +254 20 2737658
Email: ebukusi@rctp.or.ke

Protocol Virologist:

Teri Liegler, PhD
Associate Professor, Medicine-HIV/AIDS
Director, ARI-UCSF Laboratory of Clinical Virology
University of California, San Francisco
UCSF Box 1234
San Francisco, CA 94143-1234, USA
Phone: (415) 206-8929
Email: teri.liegler@ucsf.edu

Investigator:

Jane Achan, MBChB, MPed
Makerere University
MU-UCSF Research Collaboration
P.O. Box 7475
Kampala, Uganda
Phone: +256-414-530692
Fax: +256-414-540524
Email: achanj@yahoo.co.uk

Investigator:

Craig Cohen, MD, MPH
Professor of Obstetrics, Gynecology and Reproductive Sciences
University of California, San Francisco
550 16th Street, 3rd Floor
San Francisco, CA 94143, USA
Phone: (415) 476-5874
Fax: (415) 597-9300Email: CCohen@globalhealth.ucsf.edu

Investigator:

Tamara Clark, MPH
University of California, San Francisco
UCSF Box 0874
San Francisco, CA 94143-0874, USA
Phone: (415) 206-8790
Fax: (415) 648-8425
Email: tamara.clark@.ucsf.edu

Investigator:

Elvin Geng, MD, MPH
Assistant Professor of Medicine
HIV/AIDS Division
University of California, San Francisco
UCSF Box 0874
San Francisco, CA 94143-0874, USA
Phone: (415) 476-4082, x409
Fax: (415) 476-6953
Email: genge@php.ucsf.edu

Investigator:

Gabriel Chamie, MD, MPH
Assistant Professor of Medicine
HIV/AIDS Division
University of California, San Francisco
UCSF Box 0874
San Francisco, CA 94143-0874, USA
Phone: (415) 476-4082, ext. 445
Fax: (415) 476-6953
Email: gabriel.chamie@ucsf.edu

Investigator:

Vivek Jain, MD, MAS
Assistant Professor of Medicine
HIV/AIDS Division
University of California, San Francisco
UCSF Box 0874
San Francisco, CA 94143-0874, USA
Phone: (415) 326-3402
Fax: (415) 476-6953
Email: vivek.jain@ucsf.edu

Investigator:

Dalsone Kwarisiima, MBChB, MPH
Makerere University Joint AIDS Program
Plot 4B Kololo Hill Drive
P.O. Box 7072
Kampala, Uganda
Phone: +256-772-607274
Email: dkwarisiima@mjap.or.ug

Investigator - Economics:

Harsha Thirumurthy, PhD
Assistant Professor
Gillings School of Global Public Health
University of North Carolina at Chapel Hill
Campus Box 7411
Chapel Hill, NC 27599-7411, USA
Phone: (919) 966-9756
Email: harsha@unc.edu

Investigator - Cost Effectiveness:

James G. Kahn, MD, MPH
Professor of Health Policy and Epidemiology
Philip R. Lee Institute for Health Policy Studies
University of California, San Francisco
UCSF Box 0936
San Francisco, CA 94143-0936, USA
Phone: (415) 476-6642
Email: JimG.Kahn@ucsf.edu

Investigator:

Theodore Ruel, MD
Assistant Professor of Pediatrics
Department of Pediatrics
University of California, San Francisco
UCSF Box 0136
San Francisco, CA 94143-0136, USA
Phone: (415) 476-9198
Fax: (415) 476-1343
Email: Ruelt@peds.ucsf.edu

Investigator:

Zachary Kwena, MA
Kenya Medical Research Institute
Co-Principal Investigator, FACES
Box 54840
Nairobi, Kenya
Phone:
zkwena@kemri-ucsf.org

Investigator – Qualitative Studies:

Carol Camlin, PhD, MPH
Assistant Professor
Department of Obstetrics, Gynecology and Reproductive Sciences and Center for AIDS Prevention Studies
University of California, San Francisco
550 16th Street, 3rd Floor
San Francisco, CA 94143, USA
Phone: (415) 597-4998
Email: Carol.Camlin@ucsf.edu

Investigator – Statistics and Modeling:

Laura Balzer, MPhil
Biostatistics PhD student
School of Public Health
University of California, Berkeley
Berkeley CA 94720-7358, USA
Phone: (203) 558-3804
Email: lbbalzer@berkeley.edu

Data Manager:

Geoff Lavoy
Data Center Manager
Infectious Diseases Research Collaboration
P.O. Box 7475
Kampala, Uganda
Phone: +256 714 964 634
Email: glavoy@muucsf.org

Investigator – Statistics and Modeling:

Mark van der Laan, PhD
Professor of Biostatistics and Statistics
School of Public Health
University of California, Berkeley
Berkeley, CA 94720-7358, USA
Phone: (510) 643-9866
Email: laan@stat.berkeley.edu

Industry Representative:

James F. Rooney, MD
Gilead Sciences, Inc.
333 Lakeside Drive
Foster City, CA 94404, USA
Phone: (650) 522-5708
Email: jim_rooney@gilead.com

PROTOCOL SYNOPSIS

Overall Goal: The SEARCH study will quantify the health, economic and educational impact of early HIV diagnosis and immediate ART (antiretroviral therapy) using a streamlined care delivery system in rural communities in East Africa. The study intervention is designed to improve the entire continuum of care, to reduce structural barriers for all populations including those most "at risk" and build upon evidence based prevention interventions including adult male circumcision.

Study Hypothesis: ART initiation at any CD4 count with streamlined delivery compared to CD4-driven ART initiation will reduce cumulative 5-year HIV incidence and protect and improve health, economic and education outcomes in communities with annual HIV testing campaigns.

Study Partnerships: SEARCH is designed to inform governments and health policy makers and to benefit affected communities. To that end, SEARCH is a partnership with input and sponsorship from global and local health and development agencies, foundations, governments and the study communities. These include the ministries of health in Uganda and Kenya, the National Institutes of Health, the World Bank, and PEPFAR.

Study Design: SEARCH is a cluster randomized community trial. Community health campaigns will be conducted in all study communities at baseline and will offer HIV testing and multi-disease prevention and treatment services. The intervention is annual and targeted HIV testing and ART independent of CD4 cell count delivered in a streamlined approach for all HIV infected adults and children. Components of streamlined care include ongoing HIV combination prevention strategies including male circumcision. Control communities will follow country guidelines for ART.

HIV incidence will be measured using an efficient community cohort design (ECCO) comprised of three key elements: A) baseline household community level census, B) community health campaigns (CHC) incorporating HIV testing that use unique identifiers to link individuals between successive waves of the intervention, and C) tracking and evaluation of individuals who do not participate in CHCs.

Study Population: Thirty-two communities with a population of approximately 10,000 persons each will participate in the following three regions: A) Mbarara/Western Uganda (n=10), B) Tororo/Eastern Uganda (n=10), and C) Southern Nyanza Province, Kenya (n=12). Randomization to intervention vs. control will occur in pairs of communities matched based on key health, geographic and ethnographic variables including: A) geographic region B) population density C) number of trading centers D) transportation index, and E) occupational mix.

Primary Endpoint: Cumulative 5-year HIV incidence in men and women ages ≥15 years.
Secondary Endpoints: The health-related secondary endpoints include: 1) mortality (overall, maternal, and infant mortality), 2) mother-to-child HIV transmission, 3) AIDS (WHO stage 4), 4)

tuberculosis, and 5) HIV drug resistance. The economic/education secondary endpoints include: 1) adult and child employment levels, 2) asset holdings, 3) school attendance levels, 4) programmatic costs, 5) health gains expressed in averted Disability Adjusted Life Years (DALY), and 6) cost effectiveness (e.g. cost per infection averted and per DALY averted). Other secondary endpoints include: cumulative 3-year HIV incidence in men and women ages ≥15 years; implementation of the HIV, hypertension and diabetes care cascades including testing, linkage and retention to care; and attitudes of community, patients and providers on care delivery in control and intervention communities.

Study Antiretroviral Treatment Regimen: The study intervention is provision of ART for all individuals at any CD4+ cell count. ART – the regimen of efavirenz, emtricitabine and tenofovir disoproxil fumurate or equivalent – will be provided by the study for those who do not meet in-country guidelines to start ART. These individuals will be guaranteed 3 years of ART. After three years, these individuals will continue uninterrupted ART provided by their country of residence through agreements with the Ministry of Health.

Study Duration: The study will follow the communities for 5 years after the first community health campaign.

Statistics: We are powered to detect a 40% reduction in 5 year cumulative incidence in treatment versus control communities under conservative assumptions regarding plausible values for cumulative incidence in the control communities, baseline HIV prevalence, incomplete follow up, and between-community variation (matched pair coefficient of variation). Estimation of cumulative incidence within each community will account for incomplete attendance at the community health campaign and tracking. Primary analyses will consist of pair matched community level analysis.

GLOSSARY

3TC	Lamivudine
ABC	Abacavir
AFB	Acid-fast bacilli test
ALT	Alanine aminotransferase
ANC	Absolute neutrophil count
ART	Antiretroviral therapy
ATV	Atazanavir
AZT	Zidovudine
BUN	Blood urea nitrogen
CO2	Bicarbonate
CBC	Complete blood count
CHC	Community health campaign
CHR	Committee on Human Research, UCSF
CI	Cumulative incidence
CK	Creatine kinase
CL	Chloride
DAIDS	Division of AIDS, NIH
DALY	Disability Adjusted Life Years
ECCO	Efficient community cohort design
EFV	Efavirenz
eGFR	Estimated glomerular filtration rate
FTC	Emtricitabine
GIS	Geographic information system
GPS	Global positioning system
HBV	Hepatitis B
HCG	Human chorionic gonadotropin pregnancy test
ICER	Incremental Cost-Effectiveness Ratio
IRB	Institutional Review Board
K	Potassium
KEMRI	Kenya Medical Research Institute
LPV	Lopinavir
MDRC	Modification of Diet in Renal Disease (formula)
MTCT	Mother-to-child transmission
MU	Makerere University
Na	Sodium
NDA	National Drug Authority in Uganda
NIAID	National Institute of Allergy and Infectious Diseases
NNRTI	Non-nucleoside reverse transcriptase inhibitor
NRTI	Nucleoside reverse transcriptase inhibitor
NVP	Nevirapine
RDT	Rapid diagnostic test
RNA	Ribonucleic acid (e.g., HIV-1 plasma RNA)
RTV	Ritonavir
SOM-REC	MU School of Medicine - Research and Ethics Committee
TB	Tuberculosis
TDF	Tenofovir disoproxyl fumarate
TMP/SMX	Trimethoprim/sulfamethoxazole
UNCST	Uganda National Council of Science and Technology
WHO	World Health Organization

1.0 STUDY CONTEXT

1.1 Background

The SEARCH study is designed to test the impact of a bold intervention in rural East Africa – treatment of all HIV-infected persons from near the onset of infection—on community health. The HIV epidemic has decimated health, education and economic gains that were made in Africa in the 1970s, leaving many countries with decreased life expectancy and mortality rates not seen in the US since the early 1900s [1]. HIV's effects on the population level are amplified by disabling the work force, damaging maternal health, increasing orphans and fueling the overlapping epidemics of TB and malaria [2-5]. The HIV epidemic represents one of the greatest public health challenges of all time.

The combination of prevention efforts and antiretroviral therapy (ART) has reduced the incidence of new HIV infections as well as mortality over the last decade. However, there were still over 2 million new HIV infections last year, and over 20 million individuals have died from HIV [1]. HIV is the leading cause of death among women of reproductive age in Sub-Saharan Africa. At the beginning of the 21st century, global approach to deploy ART to reduce AIDS mortality prioritized treatment for the most ill patients based on CD4 cell count. In Uganda and Kenya, ART is generally administered in adult patients when their CD4 count falls below 350 cells/mm^3, when they are diagnosed with a WHO Stage III or IV disease, and in other patients at high risk of HIV disease progression, including those with tuberculosis (TB). New data show that treating HIV *early* can prevent AIDS and prevent TB, a leading killer of HIV infected patients [6]. New data also show that ART can reduce HIV transmission by 96% in HIV sero-discordant couples [7]. These data illustrate a dual purpose for ART – prevent AIDS (including TB) in the HIV infected individual *and* prevent HIV transmission to the uninfected partner. The identification of HIV infection early and initiation of treatment thus has the potential to influence the overall health of the community as well as its economic and educational strength and viability.

The proposed community randomized trial will quantify the effect of an early HIV diagnosis and ART approach ("test and treat") on the health, economic productivity and educational outcomes of rural communities in East Africa. There have been several mathematical models, including a landmark publication by Granich et al and a subsequent economic analysis, showing frequent HIV testing and ART will reduce overall HIV incidence over a period of 5 to 10 years, and that the upfront investments required for such an approach result in net savings over 13 years in South Africa [8]. These models have generated heated debate within the scientific community based on various assumptions inherent in the models [9, 10]. These models may have also underestimated the benefits of ART because the evaluation framework did not include all the health benefits of ART, such as prevention of TB or the socioeconomic benefits of the preservation or return of good health afforded by ART. Thus it is time to test a population-based approach to early HIV diagnosis and treatment approach and to evaluate its cost and effects with an evaluation framework that includes health, economic and education metrics [11-13].

Inherent to the scale up of HIV treatment in general, and an important part of the SEARCH is the need to develop new models of chronic health care delivery at the community level that are

lower in resource needs and are sustainable. Finding patients earlier in HIV disease, keeping these individuals healthy with early ART, and delivering their care in a streamlined manner in fact may be the only viable path to deal with the health care worker shortage which is amplified by late HIV diagnosis, and the medical expertise and facilities needed to care for patients who present and are treated after HIV has significantly progressed.

Also inherent to the design of the SEARCH intervention is the recognition that a “test and treat” approach must find an overwhelming majority of HIV infected individuals, improve upon the entire continuum of care including participation of the most at risk populations and build upon prevention interventions known to work such as male circumcision [14, 15]. The SEARCH study is built upon biomedical evidence that is being applied and tested in a manner which incorporates social science evidence and approaches.

The SEARCH study is multicounty collaboration built upon expertise from a broad spectrum of scientific disciplines. It is grounded upon partnerships with scientific, health, and development global agencies. SEARCH is designed to inform the health sector, finance ministries, and the scientific and lay communities on the medical and economic effects of early antiretroviral therapy in rural East Africa.

1.2 Rationale

There is overwhelming evidence that the benefits of ART extend well beyond those originally appreciated, and those that are currently measured. ART reduces mortality among persons with HIV. The HPTN 052 study showed that ART reduces AIDS related illness even in persons with CD4 cell counts above thresholds of CD4 cell counts (i.e. 350 cells/mm3) [7]. ART also dramatically reduces TB risk both on the individual and the community level [6, 16, 17]; reduces the risk of malaria in individuals [18]; Reduces mother to child transmission (MTCT) of HIV; and reduces maternal and child mortality [19, 20].

ART is also a key component in a multilevel HIV prevention strategy. The HPTN 052 study shows definitively that ART reduces HIV transmission and preserves the health of the HIV infected persons receiving treatment [7]. These results build upon prior observational cohort studies showing reductions in HIV transmission by ART in HIV sero-discordant couples [21]. Biomedical interventions such as ART are likely to be highly complementary to proven interventions such as male circumcision and have an important role in reducing new HIV cases.

ART and the associated restoration of health also have important effects on socio-economic outcomes. Studies conducted in various settings in sub-Saharan Africa and South Asia have documented a significant improvement in the employment outcomes of adults following the initiation of ART [15, 22-25]. These studies have shown a large and rapid increase in labor supply and labor productivity, from levels that were initially very low to levels that were similar to those of HIV uninfected adults. In many cases, the increase in employment outcomes took place within 3-6 months of ART initiation, a result that is consistent with the rapid improvement in health and functional capacity due to ART. Furthermore, studies have found that the treated patients’ family members (particularly children) also benefit substantially when a working-age

adult becomes healthy and productive [26]. Following ART initiation in Kenya, there was a significant increase in the school attendance of children living with treated patients, as well as a reduction in child labor and improvement in nutritional status. These studies suggest that earlier ART initiation would *prevent* a decline in socio-economic status and help to protect living standards.

ART can contribute to prevention of new HIV infections and prevention of AIDS and TB, enhance economic productivity, and improve socio-economic outcomes more generally. A key question then is why is the ART "test and treat" strategy not being deployed?

First the strategy requires knowledge of HIV status, and globally, most individuals are not aware of their HIV status. *We aim to identify HIV status throughout the community through annual community health campaigns that we have piloted and refined in western rural Uganda*. Second, there is a stigma for participating in care that prevents even those with known HIV infection from getting treated. *This study will be conducted in communities where we have established ongoing community engagement work, and care delivery will be adapted to the community to promote participation and retention*. Third, there are few data in developing nations to prove or disprove that asymptomatic individuals will initiate and adhere to ART. *We are currently studying predictors of adherence in this population and will incorporate this knowledge into care delivery*. Non-adherence to ART has serious consequences for the HIV epidemic because it means that A) individuals will not immediately benefit, B) HIV can become resistant requiring new and more expensive medications, and C) resistant HIV can spread sexually and during mother to child transmission.

Community engagement strategies will be deployed in this study to maximize adherence. Finally, ART (despite 10-fold reductions in drug price over the last decade) is expensive to deliver. Providing it according to the current health delivery system might not be possible due to shortages in financial and human resources, particularly when doubts are expressed about the cost-effectiveness of such an intervention relative to many other priority health interventions. Thus data are needed to inform policy makers about the full range of benefits and risks of a test and treat ART strategy. During the course of the study, we will also use social science approaches to understand how the attitudes of the community, patients and providers influence the results of the study.

1.3 Antiretroviral therapy

The 2010 WHO Antiretroviral Treatment Guidelines recommended ART for all adults with WHO stage III or IV disease, tuberculosis or CD4≤350 cells/mm3. ART was recommended for all children less than 2 years of age, and subsequent ART is dependent on a variety of disease and CD4 specific criteria. The WHO recommended a variety of approaches of ART for HIV+ pregnant women who otherwise do not meet the adults guidelines for ART. The specific country guidelines for ART treatment for countries participating in SEARCH starting in October, 2011 are summarized in Table 1 below.

Table 1: 2010 ART treatment guidelines

Country	Population	ART initiation
Uganda[27]	Adults and children ≥5 years	CD4 count ≤350 cells/mm^3
	Adults and children co-infected with tuberculosis (TB), co-infected with hepatitis B (HBV), with WHO Stage III or IV disease, and pregnant women (prophylaxis)	Initiate ART irrespective of CD4 % or count
	Children 2 to <5 years	CD4 % <25% or CD4 count <750 cells/mm^3
	Children <2 years	Initiate ART irrespective of CD4 % or count
Kenya[28]	Adults and children ≥12 years	CD4 count ≤350 cells/mm^3
	Adults and children co-infected with tuberculosis (TB), co-infected with hepatitis B (HBV) with evidence of liver damage, with WHO Stage III or IV disease, with HIV-associated nephropathy, and pregnant women (prophylaxis)	Initiate ART irrespective of CD4 % or count
	Children 5 to 12 years	CD4 % <20% or CD4 count <500 cells/mm^3
	Children 2 to <5 years	CD4 % <25% or CD4 count <1000 cells/mm^3
	Children <2 years	Initiate ART irrespective of CD4 % or count

In 2013, the WHO revised ART initiation guidelines, recommending antiretroviral therapy for persons with CD4+≤500 cells/ml, all pregnant women , HIV serodiscordant couples and other most at risk populations.. These recommendations are summarized at http://apps.who.int/iris/bitstream/10665/85321/1/9789241505727_eng.pdf. These guidelines were adopted in Uganda and Kenya in 2014. In this protocol, the intervention arm will provide antiretroviral therapy for all community members who otherwise do not meet the country criteria for ART initiation. The selection of antiretroviral regimen for this study was based upon the following regimen characteristics: antiretroviral therapy efficacy, safety profile, monitoring requirements, pill burden, knowledge of HIV drug resistance that emerges under the drug use, prior use and experience within the study countries, consultation with in- country advisory board, consultation with in -country regulatory bodies The following regimens will be provided to

the study populations below in the intervention arm that otherwise do not meet criteria for government supported ART.

Table 2: Study Treatment[1]

Population	First line regimen	Recommended substitutions/second line regimens
Adults and adolescents, 13 years and above	Emtricitabine or lamivudine, plus tenofovir disoproxil fumurate and efavirenz	Abacavir or zidovidine plus lamivudine plus atazanavir/ritonavir or lopinavir/ritonavir[2]
Children 3-12 years	Abacavir, lamivudine, and efavirenz	Zidovudine, lamivudine and lopinavir/ritonavir
Children <3 years	Abacavir, lamivudine, and nevirapine	Zidovudine, lamivudine and lopinavir/ritonavir
Pregnant women and women attempting conception[3]	Emtricitabine or lamivudine, plus tenofovir disoproxil fumurate and efavirenz.	Abacavir plus lamivudine plus atazanavir/ritonavir or lopinavir/ritonavir or nevirapine

1. Exceptions to the regimens can be made in accordance with in-country treatment guidelines
2. Second-line therapy
3. Women may receive ART via in-country program (not study intervention) if ART is started immediately and lifelong (option B plus)

2.0 STUDY OBJECTIVES

2.1 Primary Study Objective

To determine the effect of a strategy to start ART in HIV diagnosed persons at any CD4 count with streamlined delivery of HIV care compared to a country based ART guidelines on 5-year cumulative HIV incidence in rural communities with annual HIV testing.

2.2 Secondary Objectives – Health

2.2.1 To compare the three year cumulative incidence of HIV infections between the 2 study arms.

2.2.2 To compare time from diagnosis to AIDS between the 2 study arms.

2.2.3 To compare incidence of AIDS-defining events between the 2 study arms.

2.2.4 To compare proportion of total TB and incident TB cases associated with HIV between the 2 study arms.

2.2.5 To compare mortality between the 2 study arms.

2.2.6 To compare maternal and child mortality between the 2 study arms.

2.2.7 To compare mother to child transmission between the 2 study arms.

2.2.8 To compare population HIV RNA metrics between the 2 study arms.

2.2.9 To determine the association between population HIV RNA metrics and HIV incidence.

2.2.10 To compare the prevalence of transmitted HIV drug-resistance mutations and pharmacologic measures of ART between the 2 study arms.

2.2.11 To compare rates of linkage to and retention in care for HIV between the 2 study arms.

2.2.12 To compare time to ART-initiation between the 2 study arms.

2.2.13 To characterize treatment outcomes in high CD4 count individuals (CD4>350) including: A) CD4 cell count recovery, B) rate of virologic suppression, C) treatment-associated toxicities and grade 3 and 4 adverse events, and D) HIV drug resistant mutations after 1 and 2 years of treatment.

2.2.14 To compare the five year cumulative incidence of internally derived HIV infections (infections genetically linked to a prior infection among members of the same community) between the 2 study arms.

2.2.15 To evaluate attitudes of community, patients and providers on care delivery in control and intervention communities.

2.2.16 To evaluate implementation of other disease care cascades (hypertension, diabetes, women and children health services) including testing, linkage and retention to care.

2.3 Secondary Objectives – Economic and Education Outcomes

2.3.1 To compare the trends in average levels of adults' on- and off-farm employment between the 2 study arms.

2.3.2 To compare the trends in average levels of children's on- and off-farm employment (child labor) between the 2 study arms.

2.3.3 To compare the trends in average levels of children's time allocation to schooling and household activities between the 2 study arms.

2.3.4 To compare the trends in average asset holdings (durable good and livestock) between the 2 study arms.

2.3.5 To compare the trends in agricultural output and other economic production, such as fishing, between the 2 study arms.

2.3.6 To compare the trends in average levels of cash and in-kind transfers between the 2 study arms.

2.4 Secondary Objectives – Cost and Cost-Effectiveness

2.4.1 To compare costs of programming (campaigns, ART) between the 2 study arms: overall; per person identified, linked to care, and started on ART; and per ART-month, CD4 level recovered, and viral load suppressed.

2.4.2 To compare disease burden (expressed in disability adjusted life years, DALYs) between the 2 study arms, during and modelled beyond the study period.

2.4.3 To compare the savings from averted disease associated treatment costs between the 2 study arms.

2.4.4 To compare the occurrence and consequences of false positive HIV diagnosis (new).

2.4.5 To calculate the incremental cost-effectiveness of the intervention, as net cost per DALY averted.

2.4.6 To evaluate streamlined vs. non-streamlined care including time in motion studies for staff and clients.

3.0 STUDY DESIGN

SEARCH is a cluster randomized community trial. The primary study hypothesis is: ART initiation at any CD4 count with streamlined delivery compared to ART initiation according to country guidelines will reduce cumulative 5-year HIV incidence and protect and improve health, economic and education outcomes in communities with annual HIV testing campaigns. The primary study endpoint is cumulative 5 year HIV incidence in men and women ages ≥ 15 years. The study will be conducted in rural communities in Uganda and Kenya.

Community health campaigns will be conducted in all study communities at baseline and will offer HIV testing and multi-disease prevention and treatment services. The intervention is ART independent of CD4 cell count delivered in a streamlined approach for all HIV infected adults and children. This intervention will be applied in the context of ongoing HIV combination prevention strategies including male circumcision. In control communities ART will be provided by country programs according to their guidelines.

HIV incidence will be measured using an efficient community cohort design (ECCO) comprised of three key elements: A) baseline household community level census, B) community health campaigns that use unique identifiers to link individuals between successive waves of the intervention, and C) tracking and evaluation of individuals who do not participate in annual CHCs.

4.0 STUDY POPULATION

4.1 Community Level Inclusion Criteria

4.1.1 Non-adjacent geopolitical units in south-western and eastern Uganda and western Kenya.

4.1.2 Most recent census population between 9,000 and 11,000 individuals.

4.1.3 Served by an ART providing health center.

4.1.4 Community leader commitment for study participation and implementation.

4.1.5 Accessibility to health center via a maintained transportation route.

4.1.6 Community location with sufficient distance from other potential study communities to limit contamination of intervention or control conditions (buffer zone)

4.2 Individual Level Inclusion Criteria

4.2.1 Residency of individual in community, defined as present in household for at least 6 months of the calendar year.

4.3 Community Level Exclusion Criteria

4.3.1 Presence of ongoing community-based ART intervention strategies that provide treatment outside of the current in-country treatment guidelines.

4.3.2 An urban setting defined as a city with a population of 100,000 or more inhabitants.

4.3.3 Absence of a health center able to provide ART.

5.0 STUDY INTERVENTION

5.1 Antiretroviral Therapy in a Streamlined Care Delivery

5.1.1 Streamlined ART Delivery

In this study, all HIV+ participants in intervention communities will be offered HIV therapy at ART providing health centers via “streamlined care,” in order to maximize efficiency and clinic throughput, and engender the smallest impact of expanded ART access on current clinical sites, while maintaining treatment efficacy and safety. “Streamlined care” is defined as a method of enrolling ART-naïve participants, and initiating, monitoring, and sustaining ART delivery, in a manner consistent with the principles of care outlined in Table 3, recognizing that both facilities and patterns of care will vary somewhat between sites. In addition, intervention sites will employ enhanced services for linkage and retention. These include provision of provider cell phone number for referral clinic; cell phone reminder before appointments; standardized tracking for linkage from CHC to clinic and missed visits; and accelerated ART start for patients not on treatment. For participating health centers that do not currently offer ART in this streamlined fashion, study investigators will work with clinic staff to design and adapt existing procedures to this approach. During the course of the study, we will evaluate provider and patient attitudes and the implementation of streamlined and routine care.

Table 3. Features of “Streamlined” ART Delivery Model

ART Clinic	***Healthcare Team***	***ART Monitoring***
• Rapid ART initiation available with expedited counseling# • Short throughput* for patients with no active issues • Targeted adherence support • Convenient ART refill process <br>*Throughput: time spent from clinic check-in to completion of visit #ART initiation may begin at CHC	• For stable, high CD4 patients, non-MD health care worker responsible for: 1. Screening for ART-related adverse events and toxicities 2. Dispensing, managing and altering ART regimens 3. Maintaining patient and drug accountability records <br>• Back-up support for care and consultation by physician	• Streamlined visit schedule conducted by non-MD health care workers • Targeted laboratory evaluation schedule • Viral load monitoring at 6 months after ART start and then at least yearly. Provider counseling with client on all viral load results. Back-up support by physician

Study investigators and staff may provide additional support to ART providing health centers where needed. This may include assistance with staffing, training on good clinical practices and HIV care and non-communicable disease management, operation of linkage to care procedures, and or oversight of study drug accountability.

5.1.2 Provision of ART to persons who do not qualify for treatment by country guidelines

ART study medication will be provided to participants in communities randomized to intervention who do not meet in-country treatment guidelines. The study treatment will consist of a 3-drug ART regimen that will be provided to participants by the study (Table 2).

5.1.2.1 Regimen and Administration

Adults and adolescents, 13 years and above

First Line Regimen:	Tenofovir disoproxil fumurate (TDF) 300mg, *PLUS*
	Efavirenz (EFV) 600mg, *PLUS EITHER*
	Emtricitabine (FTC) 200mg, *OR*
	Lamivudine (3TC) 150mg
Recommended Second Line Regimen:	Abacavir (ABC) 300mg, *PLUS*
	Lamivudine (3TC) 150mg, *PLUS EITHER*
	Atazanavir (ATV) 300mg and ritonavir (RTV) 100mg, *OR*
	Lopinavir/ritonavir (LPV/RTV) 200mg/50mg, administered as a fixed-dose combination

Children 3 – 12 years

First Line Regimen:	Abacavir, *PLUS*
	Lamivudine, *PLUS*
	Efavirenz
Recommended Second Line Regimen:	Zidovudine (AZT), *PLUS*
	Lamivudine, *PLUS*
	Lopinavir/ritonavir, administered as a fixed-dose combination

Individual dosing information by weight can be found in Appendix E.

Children < 3 years

First Line Regimen:	Abacavir, *PLUS*

	Lamivudine, *PLUS*
	Nevirapine (NVP)
Recommended Second Line Regimen:	Zidovudine, *PLUS*
	Lamivudine, *PLUS*
	Lopinavir/ritonavir, administered as a fixed-dose combination

Individual dosing recommendations by weight can be found in Appendix E.

<u>Pregnant women and women attempting conception*</u>

First Line Regimen:	Tenofovir disoproxil fumurate (TDF) 300mg, *PLUS*
	Efavirenz (EFV) 600mg, *PLUS EITHER*
	Emtricitabine (FTC) 200mg, *OR*
	Lamivudine (3TC) 150mg
	Or if patient provider desire: Tenofovir disproxil fumerate, *PLUS EITHER*
	Emtricitabine (FTC) *OR* Lamivudine (3TC), *PLUS*
	Lopinavir/ritonavir 200mg/50mg, two tablets PO twice daily, administered as a fixed-dose combination. After the first trimester, women may switch to efavirenz 600mg, as described above.

* Women may receive ART via in-country program (not study intervention) if ART is started immediately and lifelong (option Bplus)

Recommended Second Line Regimen:	Abacavir 300mg, two tablets PO daily, *PLUS*
	Lamivudine 150mg, one tablet PO twice daily, *PLUS EITHER*
	Atazanavir 300mg and ritonavir 100mg, once PO daily, *OR*
	Nevirapine 200mg, one tablet PO daily for first 2 weeks, then one tablet PO twice daily

Based on variations of standard first- and second-line ART regimens suggested among in-country treatment guidelines, substitutions to the above can be made at the investigators' discretion.

5.1.2.2 Product Supply and Accountability

Supply

Each participant will be provided with ART treatment over the course of his or her participation. Truvada® and efavirenz, if prescribed, will be provided by the study; other medications will be provided by their country of residence through agreements with the Ministry of Health.

Accountability

Staff will maintain records of study drugs distributed according to standard guidelines. At most sites, lot number and the number of pills given to each participant at each visit will be recorded. For Gilead provided study medications, current product labels, Certificates of Analysis, date received, lot number, expiration date, and date used will be maintained within the site regulatory binder for the study. Monthly inventory will be conducted.

6.0 COMMUNITY LEVEL STUDY EVALUATIONS

6.1 Baseline Household Community Level Census

Prior to the start of the study, study team members will meet with local officials and community representatives to discuss the study and plans for the census. Using a map of the boundaries of the selected communities, study staff will systematically cover the entire area within the boundaries to identify and enumerate all households. A head of the household will provide informed consent for the following information to be collected about household members:

- Name
- Relationship to head of household
- Mobile phone number
- Demographic information such as age, gender, occupation and marital status.
- A fingerprint of each household member will be collected with the use of a fingerprint biometric device.
- The household's location relative to the local health center will be mapped using hand-held GPS receivers.

At the time of the census, household members will be consented verbally for participation in the community health campaign and for the census itself. Household members not present at the time of the census will not be excluded from participation in community health campaigns.

6.2 Community Health Campaign (CHC)

6.2.1 Overview

A baseline community health campaign will be conducted in all study communities and will offer A) HIV testing, and B) multi-disease diagnostic, prevention, treatment, and referral services (such as malaria, deworming), tailored to the community. The CHCs will serve two primary purposes. First, the campaigns will allow for community-wide HIV testing and prompt linkage to HIV care – a critical aspect of care delivery for both intervention and control communities. Multi-disease service delivery in the context of a community health fair will encourage broad communication across all demographic groups and encourage HIV testing as a routine part of health care. Second, the campaigns will provide an evaluation framework for multiple study outcomes, including health, economic and education outcomes, such as HIV incidence, community HIV viral load, interval vital status and AIDS assessments for efficient community cohort participants. CHCs will be conducted annually in the intervention communities. CHCs will be conducted at baseline and after 3 and 5 years of follow up in the control communities. Adults who do not attend the campaigns will have follow up "tracking" visits to ascertain HIV status and offer ART start for infected persons.

6.2.2 CHC Procedures

Each community will have a baseline campaign performed within 4 months after the census and performed up to annually. Roving CHC teams assigned to specific study communities will conduct the campaigns. Campaign training will be performed at the start of the SEARCH study, and updated as needed.

6.2.2.1 Community mobilization

The primary goal of community mobilization will be to maximize CHC participation and linkage to care by informing the community of the purpose, dates and locations of the community health campaign days, and of the services that will be available. These activities will include meetings with village leaders throughout each study community, and may include poster and leaflet advertising, radio advertising and enlisting community-based volunteers to describe campaign activities and encourage participation in the CHCs and linkage to care. Community mobilization will incorporate the principles of a study community engagement plan which may include incentives at the CHC, such as a raffle for prizes, supported by the community.

6.2.2.2 CHC Services

The campaign will consist of a series of stations arranged so as to maximize participant privacy and flow through the campaign. Participants will proceed through stations such as those described below, but which could be adapted to the services provided in the individual communities. Examples of other activities that could be included are: vaccines, referral stations for adult male circumcision, women and children health services, tuberculin skin testing, urgent care station, men's health services, and dermatological screening and services.

Welcome Station

At the start of each campaign, participants will review the services offered at the campaign with campaign staff. If participants have not taken part in census activities prior to the campaign, informed verbal consent will be obtained from all adults for themselves and their children to participate in the campaign and to take part in census activities. The verbal consent form will be read to them in the local language, and their fingerprint biometric will be recorded on tablet computers as affirmation of their agreement to participate. Identifying information will be collected from each community member. Identification of participants will be based on the following: name, village of residence, age, gender, and fingerprint biometric. Campaign staff will provide each participant with a campaign results card to record their screening results. Participants will then initiate campaign activities.

Health and Socioeconomic Interview Station

Health and socioeconomic questionnaires will be performed for each participant, in order to collect updated information about health and economic status, including interim births, illnesses, hospitalizations; changes in employment or educational attainment over the past year; and migration patterns and social network. Staff will interview women of child-bearing age regarding any births and deaths of children over the past year.

Pre-test Counselling Station

Prior to undergoing diagnostic testing in the campaign field laboratory, group pre-test counselling will take place to inform and update participants on the diagnostic services offered and answer questions.

Campaign Field Laboratory

Multiple diagnostic services will be offered in the field laboratory. Some will be offered at all campaigns, and others will be recommended but not required, depending on country guidelines and local resources.

a. All participants:
    i. HIV Antibody testing for all participants >9 months (Kenya) or >18 months (Uganda) according to country policy at the baseline CHC. In subsequent years, HIV testing will be optional for persons ≤10 years of age, depending on epidemiology of region:
        1. Initial rapid HIV test
        2. Participants with a negative result will be informed that they are HIV-negative
        3. Participants with an initial positive result will undergo a second rapid HIV test. If the result is positive, participants will be informed of their HIV-positive status.
        4. Participants with discordant rapid test results (first test positive, second test negative) will undergo a third "tie-breaker" HIV test. Participants with a positive "tie-breaker" test will be informed that they are HIV positive. Participants with a negative "tie-breaker" test will be informed that they are HIV-negative.
        5. At follow up years 3 and 5, an additional confirmatory HIV test will be performed for research measurement confirmation of HIV infection and not for clinical management. At follow up year 2, this test will also be performed for a subset of communities in Uganda and Kenya for quality control of the confirmatory assay.
    ii. Dried Blood Spot on filter paper for all HIV-positive participants
    iii. At the baseline CHC, HIV testing (for HIV DNA) on Dried Blood Spots may be performed on either:
        a) all infants ≤9 months (Kenya) or ≤18 months (Uganda) of HIV positive or HIV status unknown mothers; or
        b) on infants of ≤18 months old who test rapid HIV Antibody positive, according to routine country policy.
    iv. *Optional:* Malaria rapid diagnostic test (RDT) for participants with a temperature taken at the CHC of $\geq 38^{o}C$
    v. *Recommended:* Finger-stick blood random plasma glucose measure for all participants ≥13 years and/or HB A1c screening when available
    vi. *Recommended:* Syphilis screening (RPR) for all >15 years

vii. *Recommended:* Blood pressure measurement for all ≥18 years*Optional:* Tuberculosis screening: Communities may be randomized to receive participant TB screening as part of the CHC
viii. *Optional*: Malaria screening: Communities may be randomized to collect filter paper blood spots for genotyping

b. HIV-infected participants:
    i. Point-of-care CD4 cell count testing
    ii. HIV RNA, by fingerprick or phlebotomy
c. Children (<18 years)
    i. *Recommended:* Anthropomorphic Measures: Height and weight
    ii. *Recommended*: Malaria rapid diagnostic test (RDT) for children <10 years old with a recorded temperature ($\geq 38^oC$) at the CHC

Post-test Counselling

All adult participants, regardless of test results, will receive standard post-test counselling per country guidelines, including participants newly diagnosed with HIV. For known HIV+ participants already engaged in care, counselling will be adapted to meet their needs. Child participants will receive post-test counselling with a parent or guardian. On-site malaria treatment will be available at this station as well. All malaria RDT positive participants will be offered on-site malaria treatment, according to Ugandan or Kenyan standard of care for non-severe malaria. Cases of severe malaria will be offered transportation the nearest in-patient health center for hospital-based treatment.

Linkage Station

Post-test counsellors will direct any participants with a positive screening test (i.e. HIV, hypertension, diabetes, etc.) to the referral station in order to meet disease-specific clinical staff and to schedule intake appointments for the appropriate clinic(s). Campaign staff at the referral station will focus on three aspects of linkage-to-care:

a. Patient education: All participants will receive information regarding early treatment and the benefits and importance of linking to disease-specific care after diagnosis.
b. Patient Navigation: A clinician will meet with each participant not already in care in order to introduce him or herself and answer questions regarding the referral clinic. Following this introduction, a study assistant will schedule an intake appointment, or arrange immediate linkage to the clinic if available and the participant agrees. Participants who tested HIV-negative at their prior CHC or home-based testing who are newly diagnosed with HIV may be provided with specialized counseling, including targeted information on the benefits of linking to care and addressing participants' specific fears. Participants may also be offered immediate counseling from study clinic leadership by mobile phone.
c. Structural barrier to Linkage: Transportation costs to clinics represent a major barrier to linkage to care in this study population. HIV-infected participants will

receive transportation vouchers redeemable for transportation expenses *after linking to care* for their first visit at baseline in all communities and subsequently in intervention communities. SEARCH linkage vouchers will be collected by a research assistant at the clinic to which the participant is referred at the time of linkage to HIV-specific care. HIV-infected participants may also receive a one-month supply of TMP/SMX. Some patients may also be started on ART at the CHC if agreements have been arranged with the local clinic for assurance of continued care. For HIV, hypertension and diabetes, linkage may include tracking of participants who did not link to care.

<u>Distribution Station/Campaign Exit</u>

In the final step of each CHC, participants will have their fingernails marked with permanent ("voting") ink, to prevent persons from repeating campaign activities during a CHC period, and receive several additional services:

a. All children ≥12 months and ≤5 years of age will be provided with Mebendazole (one 500 mg tablet).
b. All children ≥ 6 months and ≤5 years of age will be provided with Vitamin A supplementation (one 200,000 IU capsule)
c. Distribution of male condoms

<u>Documenting Participation</u>

Upon completion of every campaign, each community's baseline census enumeration will be compared to the list of campaign attendees using all identifying information collected at the Welcome Station (including electronic fingerprint). After each CHC, all community members (defined by enumeration in the baseline census and including HIV-infected and uninfected residents) who did not attend the CHC will be tracked and evaluated.

6.2.2.3 CHC Procedures Intervention Arm

Participants in all communities who are newly diagnosed with HIV will be encouraged to visit their local health center for care. Participants in intervention communities who do not meet country guidelines to start ART will be asked to visit the study supported ART providing health center for their community, where they will be introduced and consented to the ART Intervention study. Participants will receive transportation vouchers, which can be redeemed at the health center. The value of the transportation voucher will depend on the distance from the participant's home to the clinic. Patients may be started at ART at the CHC at the discretion of the local health care center.

6.2.3 Targeted/Supplemental HIV Testing

Existing testing services in intervention communities will be assessed at baseline, and SEARCH will partner with these services to ensure linkage to the streamlined care delivery system,

including ART at all CD4 counts for individuals diagnosed in these locations. In addition we will use HIV prevalence and incidence data from CHC testing campaigns and national HIV information to identify important most at risk populations (MaRPs) for supplemental HIV testing at time periods in between community health campaigns. Supplemental HIV testing for MaRPs will be tailored to the needs of the individual MaRP population and may include the following strategies:

- Chain referral contact (sometimes called snowball or respondent driven sampling) to access hard to reach populations. In chain referral contact “seed” members of MaRP populations are used to recruit their contacts that are also members of the MaRP risk group for supplemental HIV testing and those tested persons are then asked to recruit their MaRP contacts in a similar fashion. Each successive recruit is asked to participate in recruiting his or her MaRP contacts for testing. Numbered invitation cards and incentives may be used to enhance recruitment and testing uptake.
- Private location or home-based testing will be utilized where MaRP populations have issues of confidentiality and public stigma.
- Venue based recruiting (such as brothels or bars) will be employed where appropriate to identify MaRPs for referral to supplemental testing at an offsite private location/home or to clinic-based or community HIV testing services.
- For MaRP populations with elevated HIV incidence we may employ rapid HIV testing technologies that are able to identify individuals with acute HIV infection prior to HIV antibody responses by testing for the presence of HIV p24 antigen.
- Engagement of the leadership of social or business organizations with significant MaRP membership may be used to provide access to opportunities for recruitment for supplemental HIV testing for the organizations’ members. Recruitment may take the form of personal or group invitations at meetings, numbered invitation cards and incentives, or co-location of mobile HIV testing services at group functions as needed.
- Non-cash incentives may be used where appropriate to enhance recruitment and uptake of HIV testing among MaRPs. Appropriateness of type and amount of incentive will be informed through discussions with the study community advisory boards and key informant members of MaRP populations. Incentives may include mobile phone air time cards, transport reimbursement, or a liter of fuel for transport drivers, that does not have a value of more than $5 USD.

6.2.4 Tracking CHC non-participants

<u>Tracking Procedure</u>

Locator information collected during the baseline census enumeration will be used to locate CHC non-participants. The community tracker then records the outcome of the patient after tracking in the community. Data of new household members including immigrants will be captured as well.

<u>Tracking Evaluation</u>: Evaluation will consist of at least the following:

- Vital status with verbal autopsy in the event of a reported death, to capture cause of death (e.g. trauma, illness, suicide, childbirth) whenever possible.
- Field HIV antibody rapid testing according to the testing algorithm (with confirmation and tie-breaker testing) used in the CHC
- CD4 cell count and HIV RNA testing among HIV+ participants
- A health, economic and educational interview, similar to the interview conducted during CHCs.
- Trackers will also evaluate reasons for not coming to the recent CHC, and investigate incentivizes for participation in the subsequent CHC.
- For people who cannot be tracked: reasons for not finding the person (e.g. emigration out of the study community, migration within the study community, or other reasons).
- *Recommended for consideration*: The collection of Dried Blood Spots on filter paper, finger-stick blood glucose measures, syphilis screening (RPR) for all above 15 years, and blood pressure measurement for everyone 18 years and older.

6.3 Mortality and New Births, and HIV and TB Disease Surveillance

To maximize ascertainment of SEARCH study secondary outcomes, we will conduct community-based surveillance for key study outcomes in the one-year period between annual community health campaigns (CHCs).

6.3.1 Mortality and New Births Surveillance

Deaths and births within each study community will be ascertained using a combination of data from the CHC, post-CHC tracking and local death and birth registries. Depending on the location, staff may also work with government sponsored village health teams, village elders or community leaders, or their equivalent to obtain this information.

a) <u>CHC data</u>: During the CHC, research assistants may update each community's baseline census to reflect interim deaths and causes of death (when known) of study community members. Information (including electronic fingerprint and residence information) on all children born to members of the study community will be added to the updated census.
b) <u>Post-CHC Tracking</u>: We will update each community's baseline census based on the birth and mortality data collected during post-CHC tracking (see above).
c) <u>Local Death Registries</u>: Study staff may work with government officials to build capacity to maintain ongoing lists of deaths as they occur in their community, depending on the desires of the village representatives. Study staff will review these local death registries periodically and incorporate the information into the study census for that community.

Immediately prior to each CHC, study staff will produce an updated community census based on all birth and death data collected in the past year. The updated census will represent the target community, including documentation of CHC participation and tracking of CHC non-attendees.

6.3.2 Morbidity/Disease Surveillance, Infant Diagnosis and Care Delivery

Health Center Surveillance: Study staff will regularly collect information available from routine encounters at local health centers and, where needed, hospitals within the community. The following information will be collected but not limited to:

- Visit date and identifying information, including name, age, gender, village of residence, and fingerprint, will be collected on all clinic attendees at the time of clinic visit.
- Diagnosis
- Laboratory results
- Medications
- Hospitalizations
- Mortality

Study staff will also work with implementing partners and health facilities to ascertain HIV status of children born to HIV+ women at the heath facility.

A sample of health provider and patients will be surveyed using standard instruments on a) patient satisfaction, b) provider satisfaction, c) perception of empathy and respect in order to assess qualities of the patient-provider interaction in care delivery.

TB Surveillance: Staff will visit all clinics providing TB therapy at regular intervals throughout the year. The government is the sole supplier of anti-tuberculosis antibiotics in Uganda and Kenya, and therefore TB therapy is only available through government-run or associated clinics and hospitals. All TB clinic dispensaries keep government registries with diagnostic and treatment-related outcomes for every case, including name, age, HIV status and residence information. Staff will enumerate all clinics providing TB therapy at baseline. At the start of the SEARCH study and during regular visits to TB clinics, staff will collect the following information, as available:

- Overall interval number of TB cases reported to the clinic, with name, age, residence, and acid-fast bacilli (AFB) smear results
- TB treatment: Data on treatment status of each case at initiation of anti-TB therapy (new TB cases, retreatment TB, and TB treatment defaulters)
- TB treatment completion and failure rates.
- All-cause TB case fatality rates: the proportion of TB patients who died on TB treatment each year, excluding those cases that left the community during treatment.

6.4 Feedback of CHC Process and Clinical Data

The CHC implementation team for each community will be provided with data on CHC operations, including linkage to care, determine what, if any, alterations will need to be made to outreach, testing, tracking and surveillance procedures to make them more effective. In addition, the study team will evaluate how the intervention is affecting the community: describe

attitudes, beliefs and social norms surrounding HIV testing, disclosure, and ART; and describe experiences of HIV stigma and disclosure, experiences with engagement in HIV care and ART, and sexual behavior, among HIV-positive individuals. These modifications will be incorporated into subsequent campaigns in an ongoing effort to improve community trial efforts throughout the five-year study period. This will allow the CHC procedures in each community to evolve in response to data. Individual plasma HIV-1 RNA levels measured at the CHC as part of this research study will be provided to clinics for subjects on ART.

7.0 CLINCAL AND LAB EVALUATIONS IN ART INTERVENTION ARM

7.1 Recruitment

At the time of first awareness of HIV infection, either during a community health campaign or any other location, participants in intervention communities will be asked to visit the study supported ART providing health center in their community, where they will be introduced to the study. All patients who do not meet in-country treatment guidelines for ART will be offered to be screened for the study.   The remainder of this section refers only to patients who will receive ART from the SEARCH study because they do not meet ART guidelines.

7.2 Selection of Participants in Intervention Arm

7.2.1 Inclusion Criteria

7.2.1.1 HIV-1 infection diagnosed by a rapid HIV test or any licensed ELISA test kit. For patients diagnosed in a setting other than study-conducted community health campaigns, HIV status will be re-verified at the time of study screening.

7.2.1.2 Most recent CD4+ cell count > 350 cells/uL, performed within the past 6 months.

7.2.1.3 Willing to initiate ART.

7.2.1.4 Ability to swallow oral medications.

7.2.1.5 Ability and willingness of participant to give informed written consent.

7.2.2 Exclusion Criteria

7.2.2.1 Currently taking ART.

7.2.2.2 Allergy or sensitivity to prescribed ART.

7.2.2.3 Active World Health Organization (WHO) HIV stage III or IV disease.

7.2.2.4 Any other clinical condition that, in the opinion of the site investigator, would make the participant unsuitable for the study, unable to comply with dosing requirements or qualify the participant for ART initiation according to in-country treatment guidelines.

7.3 Informed Consent and Enrollment

Written informed consent to participate in the study will be obtained from all participants. Consent forms will be translated from the original English to the language(s) spoken in the community. The consent form will be read to participants in their local language.

After consent, participants will undergo screening procedures to determine eligibility by clinical evaluation and laboratory testing. Once eligibility is verified, participants will undergo baseline procedures and to receive ART medications. Screening and baseline procedures may be done

on the same day. Patients may be re-screened for participation according to patient-provider discussions.

7.4 Schedule of Evaluations

Table 5: Schedule of Evaluations

Procedure or Evaluation	Screen	Baseline	Study Week						
			4	12	24	36	48	Every 12 weeks	144
Demographic information	X						X		X
Medical history	X								
Targeted physical exam		X							
Tuberculosis screen		X	X	X	X	X	X	X	X
Vital signs		X							
Symptom screen		X	X	X	X	X	X	X	X
Dispense medications		X	X	X	X	X	X	X	X
Adherence assessment			X	X	X	X	X	X	X
Documentation of HIV infection	X								
Pregnancy		X			X		X	X[1]	X
Hemoglobin		X					X		X
Liver function tests[2]		X[6]			X				
Creatinine		X			X		X[6]	X[3]	X
Glucose		X					X		X
CD4 count	X[4]								
Plasma HIV-1 RNA[5]		X[4]			X		X[5]	X[5]	X[5]
HIV seroconversion questionnaire			X[7]						

[1] Repeat every 24 weeks between Week 48 and Week 144.
[2] ALT at timepoints shown above. AST, total and direct bilirubin, alkaline phosphatase as indicated.
[3] Repeat at Week 96 only.
[4]Only if not available within the past 6 months. CHC viral load collection fulfils this criteria.
[5] Viral loads will be measured annually. Samples collected ruing annual CHC attendance, home-based testing during tracking or clinic visits will qualify for this viral load measurement.
[6]As clinically indicated.
[7]Optional; may be performed any time within the first 6 months of enrolment at the discretion of the clinician.

7.5 Timing of Evaluations

- The baseline visit must be performed within 4 weeks of screening evaluations.
- Weeks 4 through 144 must occur +/- 21 days from the protocol-specified target date.

7.6 Definitions of Evaluations

7.6.1 Demographic information

The following information will be collected: date of birth, sex, clinic ID (if applicable) and place of residence.

7.6.2 Medical history

A medical history will be collected including current health complaints and allergies to medications.

7.6.3 Targeted physical exam

Participants will undergo a physical exam covering the following systems: oropharynx, heart, lungs, skin and abdomen. Cervical cancer screening may be done in clinics where this is part of standard care.

7.6.4 Vital signs

Weight, temperature, pulse and blood pressure (in participants ≥18 years) will be recorded.

7.6.5 Symptom screen

Participants will be asked whether they currently have any active symptoms.

7.6.6 Dispense medications

Study medications will be dispensed to participants in intervention arm. The standard interval for dispensing medications is 12 weeks, but this may be extended at the discretion of the investigator for persons whose travel makes the follow up appointment not possible. It will be the responsibility of the SEARCH staff to maintain and document contact with the patient at the routine 12 week interval.

7.6.7 Adherence assessment

Participants will be asked to perform a 3-day adherence recall.

7.6.8 Documentation of HIV-1 infection

HIV-1 infection determined by previous testing will be documented at the time of study screening.

7.6.9 Pregnancy testing

Women of child-bearing potential will be tested by urine HCG (human chorionic gonadotropin). This testing will be repeated every 24 weeks between Weeks 48 and 144, or sooner if clinical suspicion of pregnancy exists.

7.6.10 Hemoglobin

This will consist of a hemoglobin measurement.

7.6.11 Liver function tests

- ALT will be performed at Baseline, Week 24 and as clinically indicated. AST, total and direct bilirubin, alkaline phosphatase will be performed as clinically indicated.

7.6.12 Creatinine

At Baseline, estimated glomerular filtration rate (eGFR) will be calculated by the Modification of Diet in Renal Disease (MDRD) formula, which is as follows:

$eGFR = 186 * \text{Serum creatinine}^{-1.154} * Age^{-0.203} * [1.21 \text{ if African}] * [0.742 \text{ if female}]$

7.6.13 Glucose

Blood glucose will be measured.

7.6.14 Tuberculosis

Tuberculosis screening will be performed according to each clinic's standard of care.

7.6.15 CD4 counts

CD4 counts will be measured at Screen only if a result is not available within the past 6 months.

7.6.16 Plasma HIV-1 RNA

This will consist of a quantitative determination of the HIV-1 plasma RNA level (in copies/mL) at study specified intervals or as clinically indicated.

7.6.17 HIV seroconversion interview

An optional interview to be completed by the study staff within the first 6 months of enrollment which will include questions to newly HIV infected subjects on their sexual partner history, prevention strategies, HIV testing experience and preferences and related issues.

7.6.18 Pharmacokinetic evaluations

In addition to the evaluations described in Table 5, pharmacologic measurements of ART levels may be performed on collected hair samples at one or more visits after Week 0.

7.6.19 Stigma questionnaire

A random subset of community members will be asked to complete a questionnaire related to HIV stigma in their community.

8.0 ART TOXICITY GRADING AND MANAGEMENT FOR INTERVENTION ARM

8.1 Toxicity Screening

At each follow-up study visit, staff will ask participants about any new symptoms. Depending on the results of this screen, staff may refer the participant to a clinician for further evaluation. Laboratory testing will be done according to the Schedule of Evaluations (Section 7.1) and results will be assessed for evidence of laboratory values indicating possible grade 3 or 4 toxicity according to the DAIDS Toxicity Table, December 2004.

8.2 Management of Laboratory Toxicities

8.2.1 Grade 1 or 2 Toxicities

Participants who develop grade 1 or 2 adverse events or toxicities may continue study medications without alteration of dosage. Participants experiencing such events will be managed at the discretion of the site investigator and staff.

8.2.2 Grade 3 Toxicities

Clinicians will evaluate all grade 3 toxicities at the time of awareness. If there is evidence that the adverse event or toxicity is NOT associated with the study drug, dosing may continue. If adverse event/toxicity IS thought to be related to study drug, ART may be withheld or switched at the clinician's discretion. Participants should be re-evaluated every 1-2 weeks if possible and if patient is able to return for follow-up on that schedule, until the adverse event returns to ≤ grade 2 or until stabilized and no longer in need of frequent monitoring, to be determined by the site investigator. ART, if withheld, may be re-introduced anytime at the discretion of the site investigator.

8.2.3 Grade 4 Toxicities

If a symptomatic grade 4 adverse event or toxicity develops, ART should be withheld or switched at the discretion of the site investigator, and the patient should be monitored frequently until the adverse event returns to ≤ grade 2 or until stabilized and no longer in need of frequent monitoring, to be determined by the site investigator.

If an asymptomatic grade 4 adverse event or toxicity develops, ART may be continued or discontinued or switched at the discretion of the site investigator.

8.3 Management of Specific Clinical Syndromes

Patients will be managed according to local standard care general guidelines, including use of the targeted management described below.

8.3.1 Rash

<u>In general</u>:

Patients will be evaluated for the severity, location, and characteristics of any rash. If any concern exists about serious medical conditions that feature a rash, such as Stevens-Johnson syndrome, clinicians will consult with the site investigator to determine the best course of action.

Issues related to EFV:

Any rash while on efavirenz (EFV) should prompt suspicion and evaluation for non-nucleoside reverse transcriptase inhibitor (NNRTI) hypersensitivity rash. This should include clinical evaluation for systemic symptoms (fever, arthralgias, myalgias), and laboratory evaluation for suggestive findings (AST/ALT >2x upper limit of normal, peripheral eosinophilia).

Participants felt to be experiencing an NNRTI hypersensitivity reaction should not be rechallenged with the suspected causal agent.

An isolated rash while taking EFV does not constitute and should not raise concern for NNRTI hypersensitivity.

For any serious rash (e.g., exfoliation, mucosal involvement, target lesions [erythema multiforme] or evidence of Stevens-Johnson syndrome):

Participants should discontinue all ART, and staff will confer with investigators as to proper management. Upon resolution to grade 1 or resolved, rechallenge with EFV ART versus restarting with a different ART can occur at the discretion of site investigator.

Issues related to ABC:

Abacavir (ABC) hypersensitivity is characterized by symptoms of rash (maculopapular or urticarial, but sometimes absent), fever, gastrointestinal symptoms, respiratory symptoms, and malaise. If ABC hypersensitivity is suspected, participants should undergo a clinical and laboratory evaluation. Elevated AST/ALT, CK or creatinine, or decreased lymphocytes, are often present.

Participants felt to be experiencing ABC hypersensitivity reaction should stop ABC immediately. They should not be rechallenged and should instead restart ART with an alternate NRTI.

8.3.2 Nausea and Diarrhea

Nausea and diarrhea are fairly common side effects patients experience during the first few weeks of ART, but usually subside and resolve promptly. Participants can be encouraged to take medicines with food, or to take anti-emetic symptomatic therapy. For diarrhea, unless an infectious cause is suspected, antidiarrheal agents may be used for symptomatic relief.

8.3.3 AST/ALT Elevation

If a participant’s AST and/or ALT are elevated >5x the upper limit of normal, including the baseline measurement, they will be referred to a clinician for immediate evaluation. Toxicity management will proceed according to the plan for grade 3 or 4 events.

8.3.4 Creatinine Increase and/or Creatinine Clearance Decrease

If a participant's glomerular filtration rate is found to be <60 mL/minute by the MDRD formula, including the baseline measurement, they will be referred to a clinician for evaluation for the need to switch ART regimen.

8.3.5 Hyperbilirubinemia

For isolated grade 3 or 4 unconjugated hyperbilirubinemia attributed to atazanavir (ATV), the drug should be continued unless associated with jaundice or scleral icterus that presents an intolerable cosmetic concern to the participant. For events that cannot be attributed to ATV or a non-study drug-related cause, clinicians will consult with the site investigator and all study medications will be held pending evaluation of etiology.

8.3.6 Management of Pregnancy

For women who become pregnant, EFV will be continued in accordance with country guidelines. EFV may also be discontinued and replaced with lopinavir/ritonavir (LPV/RTV). EFV can replace LPV/RTV after the first trimester of pregnancy is complete at the discretion of the site investigator.

8.3.7 ART Substitutions

Recommendations for alternative ART regimens are outlined in Table 2.

9.0 ECONOMIC AND EDUCATION EVALUATIONS

9.1 Overview

A longitudinal household survey will be conducted among a random sample of adult participants in order to record information about the socio-economic status of participants and their households. These surveys will provide information needed to assess the effects of the intervention on a number of outcomes related to the economic and educational status of community members.

9.2 Recruitment and Enrollment of Study Participants

9.2.1. Recruitment

Participants will be recruited after the annual community health campaigns. In each of the 32 communities, 100 HIV-positive campaign participants and 100 randomly selected HIV-negative participants will take part in the household survey from both campaign attenders and non-attenders. Selected participants' homes will be visited after the campaign using geographic data collected during the baseline household census. Participants will be asked whether they would be willing to take part in a household socio-economic survey and their identity will be confirmed by name and electronic fingerprint.

9.2.2. Informed Consent and Enrollment

Informed consent for participation in the Household Socio-Economic Survey will be conducted the participant's home shortly after the annual health campaign. Consent will be conducted in the appropriate language with the study candidates; translators will be used if necessary. The informed consent will be available in local languages and will be read aloud to the study candidates. Enrollment will be limited to adult participants in the annual community health campaigns.

9.3 Procedures

9.3.1 Household Survey

Participants will be visited at their homes by trained interviewers and consent will be obtained from those agreeing to take part. Community elders and local chiefs will be informed about the survey prior before interviewers begin visiting households. The interviewers will begin by locating the participant at the location described in the baseline household survey. We will attempt to arrange for respondents to be interviewed by somebody of the same sex. If the participant is not present, one additional re-visit to the household will be conducted at a later time. The household survey questionnaire will have a modular format, with each module covering a different topic. SOPs will be developed to provide further instructions on how the surveys will be administered. Quantitative information will be collected on various topics, such as:

- Demographic characteristics of households, such as age and sex of household members

- Health and education of household members
- Marital characteristics of household members
- Income and employment of household members
- Housing characteristics and asset ownership of households
- Transfers, gifts, and loans to and from the household
- Subjective expectations about future health and income
- Food insecurity
- Consumption and spending patterns
- Health care utilization
- HIV RNA testing (if not done within the past 3 months) and plasma for HIV drug resistance testing will be performed on persons identified as HIV+
- Anthropometric measurements may be collected as performed in the baseline CHC
- CD4 testing (optional)

The household survey will be administered in a private area so that the respondent can answer questions freely. Information about the schooling, employment and income will be collected from a knowledgeable household member in cases where the respondent is not able to provide accurate information. We will also offer tuberculin skin testing to measure latent tuberculosis infection on a population level at sites in Uganda as agreed upon with National TB Control programs. This is will be voluntary test that individuals may choose to opt out. Compensation for time to participate in the HSE survey will be made up to the amount of $10 USD per household and paid directly to the index case identified during the randomization process.9.3.2 Duration of Survey

The household survey will last approximately 2.0 hours, as is standard for comprehensive household surveys that contain multiple modules.

9.3.3 Follow-up Visits

Households that participate in the annual community health campaigns will be revisited least at years 2 and 5, and up to annually, in order to measure changes in socio-economic outcomes. A similar household survey questionnaire will be used during each visit, with additional modules that will record changes in household composition. Reasons for entry and exit of household members will be recorded.

9.3.4 Linkage to Community Health Campaign Data

Information collected in the household socio-economic survey will be linked to the data from the community health campaign by the CHC ID number of the participant who was recruited for the household survey. The linked community health campaign data will provide information on the HIV status of the participant as well as measures of the CD4 cell count and viral load of HIV-positive participants.

10.0 HEALTH CARE COSTING EVALUATIONS

10.1 Overview

We will undertake a micro-costing of the resources needed to carry out the activities contemplated in achieving this project's primary objectives. Activities to be costed include both the community health campaigns, and the provision of ART including pMTCT to both the intervention and control communities. The unit costs of the full range of services provided will be calculated. When combined with incidence data for HIV, as well as all-cause mortality and morbidity, these data will be used to estimate the incremental cost-effectiveness of the intervention.

10.2 Recruitment and Enrollment of Study Participants

As described in section 7.1, either during a community health campaign or upon testing at a health care facility, participants in intervention communities will be asked to visit the ART distributor main facility for their community where they will be introduced and enrolled into the study. The medical care resources required by each enrolled patient will be assessed as follows. A cost analysis will be carried out at each of the participating government health facilities that provides ART to enrolled patients and at that and other facilities that provide non-ART care to study patients for malaria, TB and the chronic diseases that the community campaigns seek to mitigate. We expect to complete the cost analysis at the health facilities providing ART within the study communities.

10.3 Procedures

To assess the costs of the additional activities assessed in this study, we will conduct incremental unit cost analyses using standard micro-costing techniques [29]. Incremental unit cost comparisons will be completed, based on the costs of implementing the community campaigns and the five-year costs of providing ART. This will also include time in motion studies.

10.3.1 Cost Data Teams

Incremental costs of the interventions will be assessed using a uniform cost data collection protocol for gathering expenditure data at each of the study sites. We will work in close consultation with staff at each site to complete this protocol retrospectively over the 5 years of follow-up. Following training and instrument piloting to be conducted prior to study initiation, the data collection effort will carried out by three teams, one in each study area Each team will consist of a medically trained person coupled with a person trained in finance, economics or accounting. They will be supervised by a senior in-country expert who will coordinate and communicate with the SEARCH economics team. We anticipate that the initial visit to each site will require 5 days to complete the cost instruments but that this will drop to 2 – 3 days during the subsequent visits.

10.3.2 Organization of Cost Data

Expenditures will be classified in one of four categories; (i) personnel (including fringe benefits); (ii) recurring supplies and services; (iii) capital and equipment; and (iv) facility space (as appropriate). We will also collect retrospective expenditure data to document program start-up costs. The costs of each program activity will be identified through interviews with administrative, finance and human resources officers, supplemented by direct observation in a limited number of formal time and motion studies. The costing approach will emphasize resources utilized, rather than out-of-pocket costs. For example, where expenditures do not fully reflect the opportunity cost of the resources used (e.g., donations or transfer payments), we will adjust the valuations accordingly. Costs for capital items will be amortized on a straight-line basis over their expected useful life, and assuming no salvage value. Facility space required by the interventions, will be valued at the market rental rate. Following assignment of expenditures to these four broad categories, we will further allocate each expenditure item across three areas, (i) service delivery; (ii) staff training directly related to service delivery; (iii) indirect costs consisting of intervention overhead and administration.

10.3.3 Personnel Costs and the Allocation of Overhead Across Activities

Overhead and administrative costs will be allocated to the programs in proportion to the full-time equivalent staff (FTEs) that study intervention service providers constitute of all service provider FTEs at the study sites [30]. We expect that the preponderance of intervention costs will be personnel time. The appropriate approach to measuring personnel time will depend upon the way services are organized at the study sites. For example, if dedicated staff is hired specifically for these interventions, costs can be obtained directly from compensation data. In the more likely case that service providers have multiple responsibilities, the time dedicated to these interventions can be obtained via interviews supplemented by direct “time and motion” observations, including completion by staff of logs recording major activities, for one week periods approximately six months apart.

10.3.4 Measuring Unit Costs

Outputs (denominator of the unit cost) include the numbers of patients receiving each type of study-supported services. Unit costs are defined as the relevant program costs divided by each of these outputs, respectively. To supplement this information, we will also collect information on patient-level contact hours, to allow us to examine the importance of participant and intervention-level factors related to variation in unit cost. We will also assess the variation in unit costs across the study sites and identify the major determinants of that variation. If possible, we will document changes in unit cost over time as programs potential achieve greater scale and administrative efficiency. These findings are intended to provide program managers with insights into costs structures that may be used to enhance program efficiency. We will calculate the cost per added person receiving ART, NCD and pMTCT interventions, based on other study findings.

10.3.5 Health Care Utilization and Spending by Households

The household survey mentioned in section 9.3.1 will include a section on health care utilization, using short term (1 month) recall for care sought for illness episodes and longer term (6 months)

for inpatient hospital care. These questions will identify the range of health care providers used, the frequency, and family expenditures.

11. STATISTICAL PLAN

11.1 Overview of Study Design

This is a community-level cluster randomized controlled trial, in which 32 communities in three sites in East Africa (two in Uganda and one in Kenya) will be randomized to either an intervention arm, consisting of annual community-health campaigns including voluntary counseling and testing for HIV along with a strategy of HIV antiretroviral therapy for all HIV infected persons regardless of CD4 cell count (Universal ART) coupled with a streamlined ART delivery system, or to a control arm, consisting of baseline community-health campaigns including voluntary counseling and testing for HIV and the current country standard guidelines for the initiation of HIV antiretroviral therapy for HIV infected persons (Standard ART). As the study is testing a community-level strategy, communities – rather than individuals – are the unit of randomization. An individually randomized trial could be used to study the effect of standard ART compared to ART at all CD4 counts on individual outcomes. In contrast, our interest is in the impact of a community-wide universal ART strategy, as compared to a standard ART strategy, on HIV incidence and a range of secondary community level health, economic, and educational outcomes.

Randomization will take place within pair-matched communities. Communities will be matched on site region and major factors influencing HIV transmission dynamics and health care delivery system structure. The primary outcome measure is five year cumulative HIV incidence. This will be measured for each community using an efficient community cohort design, in which a) community members are enumerated using a baseline household based census; b) individuals are serially assessed for HIV status at community health campaigns; and, c) individuals failing to participate in each community health campaign are tracked and receive home-based HIV testing. Community-level cumulative HIV incidence, together with all secondary outcomes for which sufficient data are available, will be evaluated 3 and 5 years after the first community health campaign.

a. Target Population of Communities
   The target population that we wish to generalize the results of this research to are rural and semi-rural African communities with moderate levels of HIV prevalence and incidence and served by health centers within or adjacent to the community. We are targeting communities of approximately 10,000 persons, a size which fosters social familiarity and connectedness, and which are organized as one or two adjacent geopolitical units served by a common health center. Community has in past work been defined as groups of individuals who live next to one another and participate in common practices; depend on one another; make decisions together; identify themselves as part of something larger than the sum of their individual relationships; and commit themselves to the group's well-being [31, 32]. Our target communities for this study represent units of organization that reflect these dimensions of communality.

b. Selection of Countries
   The two countries participating in this study (Uganda and Kenya) were chosen to meet the criterion that HIV incidence could be used as the primary endpoint, and that shared

common features of HIV/TB co-infection, and general levels of maternal and child mortality and economic and educational structure and productivity.

c. Selection of Site Regions
We determined, on the basis of the power analysis, that 32 communities are needed to test the primary study hypothesis (see Section 11.5.5). The study will be conducted within three site regions in two countries, in an effort to balance feasibility and cost concerns with generalizability and protection against potential regional instability. We have chosen two site regions in Uganda – Western Uganda centered on the Mbarara District, Eastern Uganda centered on the Tororo District and in Kenya – Western Kenya centered on southern Nyanza Province. Each of the two Uganda site regions (Western, Eastern) will have 10 study communities each and within Kenya, the Nyanza Province will have 12 study communities. With 10-12 study communities per site, a central study operations center may efficiently serve these widely separated rural study communities.

d. Selection of communities from target population
We identified a subset of 54 candidate communities from the target population based on the following criteria:

i. Inclusion criteria:
1) Most recent census population between 9,000 and 11,000 individuals.
2) Served by a government health center already providing ART or a highly functioning health center at one organizational level below those generally providing ART
3) Community leaders' consent to ethnographic mapping.
4) Accessibility to health center via a maintained transportation route
5) Community location with sufficient distance from other potential study communities to limit contamination of intervention or control conditions (buffer zone).

ii. Exclusion Criteria:
1) Presence of ongoing community-based ART intervention strategies that provide treatment outside of the current in-country treatment guidelines.
2) An urban setting defined as a city with a population of 100,000 or more inhabitants.
3) National government not willing or opposed to support commodities needed for Community Health Campaign, if provided by an outside organization.

e. Rationale for Selection of Study Communities and Use of Matched Pairs
Fifty-four communities were chosen using the systematic selection criteria listed in section 11.1.d. Our study design calls for the creation of 16 matched community pairs within which study randomization will take place. The rationale for matching in this setting is three-fold: 1) matching can increase study power and the precision of effect estimates if communities are matched well on factors closely associated with the study

outcome of interest; 2) we propose to match on more community level drivers of HIV transmission than can be accommodated by the alternative approach of stratified randomization (given the sample size of 32 communities); and, 3) prior experience from HPTN-043 has shown a high community acceptability of the matched pair design and allows for the utilization of validated procedures and community preparedness protocols from Project Accept.

f. Criteria for Community Pair Matching
Communities will be matched based on the following criteria: 1) site region, 2) population density, 3) number of trading centers in the community, 4) major occupational mix category (mixed agricultural, mining, tea plantation, fishing), and 5) migration index (measure of mixture with outside communities). The top 16 pairs of matched communities will be selected.

11.2 Primary Outcome Measurement

The primary outcome measure for this community cluster-randomized trial is community specific 5-year HIV cumulative incidence (CI). The general framework for measuring cumulative incidence is a community cohort of HIV uninfected persons identified at baseline in each community. Community membership will be identified through a community-wide, brief household enumeration done at baseline. HIV status of individuals in the cohort will be assessed at baseline and after 3 and 5 years of follow up through HIV testing at a community health campaign with tracking and home-based HIV testing for individuals failing to participate in the community health campaign. Each of these steps is described in greater detail below.

a. Baseline Household Enumeration
At baseline in each community we will perform a simplified community-wide, brief household enumeration to identify community members. Staff in cooperation with community volunteers will conduct an enumeration of households in the community and GPS coordinates will be recorded. Each household will be approached, and a head of the household will be provided with an explanation of the study. A minimum of 2 repeat visits to the household will be made until contact with a head of the household is made. An enumeration of the members of the household will be conducted with a head of household listing the names, age, sex, relationship to head of household, occupation, length of residence, and general travel history/frequency for each household occupant. Inclusion and exclusion criteria for eligible participants in the community cohort are:

i. Inclusion Criteria:
1) Stable residency of individual in community, defined as present in household for at least 6 months of the calendar year
2) Able and willing to provide verbal informed consent.
3) For legal minors and children-consent of legal parent or guardian.

b. Cohort Participant Identification
To aid in the identification of community participants over time, eligible persons in the household will be approached for consent to participate in the community health

campaign and tracking activities. Information to be collected includes names, alternate contact information such as nearby relatives or mobile phone numbers, and a unique biometric identifier generated electronically from the individual's fingerprint using a portable computer.

c. <u>HIV Testing in Community Health Campaign</u>
HIV testing for community cohort participants will be conducted as follows: Identity confirmation using biometric fingerprint identifier will be conducted for all subjects. For those who have not participated in biometric identification, consent and fingerprint ID will be performed along with collection of tracking information as in the baseline household census. All participants will answer a brief questionnaire regarding prior HIV testing. Rapid HIV testing using a serial HIV testing algorithm will be performed using an initial rapid HIV test. All negative results will be informed of their HIV-negative status. All positive results will be confirmed with a second rapid HIV test. Discordant results (first test positive, second test negative): participants with discordant rapid tests will undergo a third "tie-breaker" HIV rapid test. Participants with a positive "tie-breaker" test will be informed that they are HIV positive. Participants with a negative "tie-breaker" test will informed that they are HIV-negative.

At participating sites, we will obtain from participants with positive rapid HIV antibody test results 1) a rapid CD4+ T-cell count test, 2) dried blood spots, and 3) blood plasma.

d. <u>Definition of HIV Uninfected Community Cohort</u>
Individuals who are 15 years of age or older, have HIV tested HIV seronegative and are eligible per inclusion criteria (stable residency and informed consent) will be considered to be part of the HIV Uninfected Community Cohort. Persons who subsequently migrate into the community following the baseline enumeration will have data collected but not be considered as part of the Community Cohort for purposes of measurement of community specific HIV cumulative incidence (although data on these individuals will still be collected and can be used in secondary analyses). Participants with evidence of HIV care (as recorded on Ministry of Health records) prior to the date of the baseline CHC will be excluded from the incidence cohort. In order to ensure non-differential application of this exclusion criteria, enumeration data from all SEARCH participants (including name, sex, age, and parish/sub-county) will be matched to existing data from all primary SEARCH clinics (Uganda) and the FACES OpenMRS system (Kenya).

e. <u>Tracking and HIV Testing for CHC Non-Participants</u>
Biometric identifiers will be used to identify those community members enumerated during the baseline census who fail to attend the baseline CHC. These individuals will be tracked by the community tracker using locator information collected during the baseline census enumeration

When located, if alive an HIV antibody rapid test will be performed, and if positive the individual will be referred to care in accordance with treatment arm. At following subsequent CHCs, the identical procedures will be performed among all enumerated community members who fail to attend the CHC.

11.3 Randomization of Community Pairs

11.3.1 Randomization Strategy

We will utilize an established randomization strategy that is both scientifically valid and transparent to the community stakeholders and uses local idioms to make the concept of randomization easily understood by traditional leaders and community members [33]. Community randomization is conceptualized as an ongoing part of the community preparedness process in partnership with the community leaders and community individuals. An example of this strategy is described below:

To explain the concept of a randomized controlled trial (RCT) to community groups such as Community Advisory Board (CAB) members, we may use a supplementary feeding analogy that the communities are already familiar with, likening randomizing each matched pair of communities to a set of twins from one family who end up attending two different schools, only one of which offers an indigenous energy drink during the morning break to supplement the child’s lunch box from home. To explain the random allocation of communities, we will also use local language idioms that would resonate with traditional leaders, such as words meaning ‘by chance’, ‘luck of the draw’, and ‘lottery’.

For the random assignment within matched pairs of each site’s communities to intervention or control status we could employ a public lottery of community names to achieve maximum public acceptance of the randomization results by enhancing transparency and spreading ownership of the process. For each study site area, the computer will randomly designate the randomization status for each pair of study communities only as the community name that would be ‘picked up’ or ‘not picked up’ in the public lottery (see sample randomization scheme in Table 5.)

Table 6: Sample Study Site Community Pair Lottery Randomization Scheme

**Matched Pair #**	**Community Names**	**Picked-Up at Public Lottery**	
		**Picked-Up**	**Not Picked-Up**
1	Atiri<br>Mukuju	Intervention	Control
2	Malaba<br>Koitangiro	Control	Intervention
3	Paya<br>Nawire	Control	Intervention
4	Mulanda<br>Mwello	Control	Intervention
5	Nabuyoga<br>Pawanga	Intervention	Control

The lottery could be a public event with members of the CAB and local leadership present along with guests from other community-based organizations and the general public to witness the lottery conducted by local chiefs or leaders.

The lottery could be conducted in a series of draws, one for each of the matched pairs of

communities by their local chiefs or community leaders. For each drawing, the two community names are written on a separate piece of paper or card and folded in half to obscure the name from view. For the draw, the names of each matched pair could be placed in a sealed box with a hand hole cut out of the top of the box. A flip of a coin could be used to decide which of the two community leaders will pick the community name from the box. The unselected leader would be responsible for holding and shaking the selection box. The selected chief or leader will then draw a paper from the box without looking into the box and the picked-up community name was read aloud by both of the community leaders in turn. If the randomization protocol indicated that the "picked-up" community was to receive the intervention (i.e., community health campaigns and ART initiation regardless of CD4 cell count), then the host country Principal Investigator (PI) would announce the drawn community as an intervention community. If the protocol called for the "picked-up" community to be the control (i.e., community health campaigns and standard ART initiation), then the host-country PI would announce it as a comparison community. This process would assure equal chance of being randomized to the intervention or comparison arm, and eliminates any residual fears of bias or rigging.

11.4 Primary Analyses

11.4.1 Overview of Analyses for HIV Incidence Outcomes
Estimation of the impact of the intervention on five year cumulative incidence will be based on a two stage analysis. In the first stage, we will estimate cumulative incidence of HIV for each community. The community level cumulative incidence will then be compared between treatment and control arms, adjusting for a measure of population HIV RNA or baseline prevalence, with the adjustment variable chosen using a pre-specified algorithm, and accounting for the pair matched design.

In our primary analysis death will be treated as a right censoring event. In the estimation of the community specific five year cumulative incidence we will adjust in the first stage to allow for potentially informative incomplete tracking success and right censoring.

11.4.2 Data Structure

a. <u>Baseline Community-Level Data</u>
   For each community in the sample, baseline variables will be measured by the census (including population size, summaries of demographic data such as age and sex distribution, and occupational mix), and in ethnographic mapping.

b. <u>Longitudinal Individual Data</u>
   For each community in the sample, the J stable members of the community will be enumerated. We define stable as subjects who spent at least 6 months of the previous year in that community. Note that number of stable residents J is itself a community level random variable. We measure individual level data at time of census ($t=0$), including age, sex, occupation, location of residence, and marital status.

In addition, in the intervention arm in each year during the duration of the study (*t=1,…,6*), we measure on each individual an indicator of whether he or she attended the CHC and an indicator of whether he or she was tracked at time *t*. In the control arm, we collect analogous data at baseline and after 3 and 5 years (*t=1,4,6*). We will aim to track all stable community residents included in the baseline enumeration who do not attend the each CHC and have not already been documented to have died.

For individuals who come to the CHC at time *t*, we measure HIV status, and individual – specific covariates, the intervention assignment, including changes in any of the baseline enumeration variables (residence, occupation, marital status) and secondary outcomes as specified elsewhere in the protocol. For subjects who are tracked at time *t,* we ascertain their vital status and if alive measure HIV status and the individual-specific variables measured at the CHC.

c. Observed data
The observed data for a given community consist of baseline community level covariates, and *J* copies of the individual level data structure above. We observe this community level data structure on 32 pair matched communities, where one community in each pair receives the treatment and one community the control level of the intervention.

11.4.3 Estimation of Community Specific Cumulative Incidence.

a. First Stage Target Parameter
In our primary analysis, we define our HIV negative cohort as those individuals in the baseline enumeration who are alive, stable residents, ≥ 15 years of age, and HIV negative at the first CHC (or at subsequent tracking). As we aim to track and test 100% of CHC non-attendees at baseline, this cohort should be highly representative.

b. Estimation
The outcome of interest, HIV status at follow up years 3 and 5, is not observed for all subjects in our negative cohort due to incomplete CHC attendance with partial tracking of non-attendees, and due to right censoring. In the control arm there is an increased potential for informative missingness due to the longer intervals between serial testing. To address the potential bias from differential measurement of HIV between the study arms, we will use baseline and year three data for estimation of the three year cumulative incidence, and baseline, year three, and year five data for estimation of the five year cumulative incidence in both study arms. By using equivalent data in intervention and control communities, under most plausible scenarios this approach reduces bias and in simulations results in close to nominal confidence interval coverage and type I error control. In secondary analyses we will employ a mixed approach that makes full use of the annual data available in the intervention communities.

In primary analysis we will adjust for potentially informative missingness in both arms to the extent possible given measured individual level covariates using a targeted

maximum likelihood estimator[34]. Controlling for individual level covariates isexpected to reduce bias to the extent that missingness of the outcome is dependent on HIV status due to incomplete tracking success or informative censoring, and conditioning on some larger subset of the observed past removes some of this dependence. Further, adjustment for a larger subset of the past may improve efficiency, to the extent that the observed past is strongly predictive of final status. However, a larger adjustment set also runs the risk of an increased finite sample variance that outweighs any bias gains. Therefore we will also conduct secondary analysis without individual level adjustment for informative missingness.

11.4.4 Estimation of Intervention Effect.

a. Second Stage Target Parameter.
We will evaluate the effect of the intervention on HIV cumulative incidence, defined as the difference in the cumulative incidence of HIV if all communities in the sample had received the intervention versus all communities in the sample had not received the intervention. This definition provides inference for the study communities, treating them as fixed rather than randomly sampled from some hypothetical target population.

b. Accounting for the Matched Design
Under the matched design used in SEARCH, the intervention is randomly allocated within matched pairs of communities, where the matched pairs themselves are generated based on applying an algorithm to a set of candidate communities. The experiment is thus complicated by the fact that the strata (matched pairs) within which the intervention is randomized are a function of the entire sample (in particular, of the baseline community characteristics to which the matching algorithm is applied). As a result, dependence is introduced by the matching process and the observed data on the 32 communities in the sample do not correspond to 32 i.i.d copies of a random variable; nor do they correspond to 16 i.i.d. copies of a random variable. To address this challenge, we propose to apply recent work that investigates this dependence and develops estimators under this design[35].

c. Adjusted Analysis
Randomization of the intervention ensures that an unadjusted estimator will be unbiased for the causal effect of interest. However, imbalances between treatment and control communities may occur by chance, and adjustment for baseline community level covariates, which include aggregates of baseline individual level covariates, can improve efficiency and confidence interval coverage without jeopardizing unbiased estimation[35]. In particular, both baseline prevalence and population HIV RNA levels will be measured on each community at the time of the first CHC and are known to affect incidence, but are not available for matching.The limited number of communities restricts the size of the possible adjustment set, and it is not known *a priori* which baseline covariate will result in the greatest efficiency gain. We will therefore use leave-one-out cross-validation to select one baseline variable for adjustment. Our primary analysis will adjust for this community-level covariate using a targeted maximum likelihood estimator.

Inference will be based on the estimated influence curve[36]. This approach has been shown in simulations to result in excellent confidence interval coverage and type I error control while resulting in potentially substantial increases in power for testing the null hypothesis of no intervention effect.

d. Unadjusted Analysis
As a secondary analysis, we will obtain an unadjusted estimate as the mean difference in the community level outcomes in the treatment versus the control communities within matched pairs. We will use the standard variance estimator the sample variance of the pairwise differences. A paired t-test with appropriate degrees of freedom will be used to test the null hypothesis of no difference in cumulative incidence between the treatment and control communities.

11.4.4 Power and Sample Size

The trial is being conducted in 20 community pairs in Uganda and 12 community pairs in Kenya, each with a population of approximately 5000 stable adult residents. We assume a baseline HIV prevalence of 10%, HIV status measured at baseline among 80% of residents, and 75% of the approximately 3600 of those HIV negative at baseline with an outcome observed at year 5 (t=6). This yields approximately 2700 residents per community who are HIV negative at baseline and have their HIV status known at year 5 (t=6). We note that the exact number of residents per community will vary; if the actual sample size per community is at least 2700 individuals then the following calculations can be considered conservative. We further note that moderate deviations from this number of individuals are not expected to have a strong effect on power (Figure 1).

Figure 1

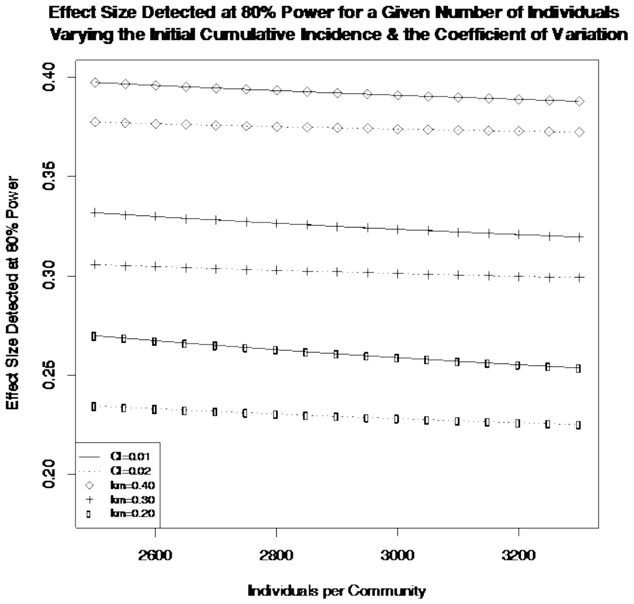

We calculated the number of matched pairs of communities needed to provide at least 80% power to detect a 40% reduction in 5 year cumulative incidence of HIV infection in the treatment versus control communities, using a two sided test at a 5% level of significance. We based our sample size calculations on the simple unadjusted effect estimator; this approach should provide a conservative effect estimate given the potential for covariate adjustment to improve precision. Sample size calculations were thus based on the formula in Hayes and Bennet for an unadjusted comparison of proportions in a matched trial [37].

Our sample size calculations assumed a 1% five year cumulative incidence in the control communities. This estimate is conservative based on the available literature, which suggests that HIV transmission rates are approximately 0.5 to 2% [38-40]. For example, assuming a current incidence density of 0.5 cases per 100 person years, and allowing for a 10% decline in transmission rate per year in the absence of the intervention (due to concurrent prevention activities), the incidence density method would suggest a five year cumulative incidence of approximately 2%. Figure 1 shows the effect size we are powered to detect under the less conservative assumption that the control proportion is 2%.

We further assumed a matched pair coefficient of variation (km) of no greater than 0.4. We note that, while ideally external data would be available to inform choice of km, 1) km values depend (among other things) on which covariates are matched on, how close a match is achieved, and the strength of association between these covariates and the outcome, limiting generalizability between studies; and 2) recent work has demonstrated the instability of estimates of km based on empirical data [32]. We note that prior studies performed in similar settings have assumed a km of closer to 0.25 (Project ACCEPT, Mwanza Trial as discussed in Hayes and Moulton 1999 [31]); in the case that this more optimistic km holds we will be powered to detect a smaller (approximately 30%) reduction in cumulative incidence. Figure 1 shows a graph of the percent reduction we will be powered at 80% to detect under a range of deviations from the assumptions above, including variation in the number of individuals per community who are HIV negative at baseline and have known HIV status at year 5 (t=6), a range of five year cumulative incidence values, and a range of kmvalues (for 16 matched pairs, and correcting for the loss of degrees of freedom when using a paired t-test, page 115 [31]).

11.5 Secondary Analyses

11.5.1. Secondary Health Outcomes

a. Overview.

In addition to estimating the impact of the treatment on expected 3 and 5 year cumulative incidence of HIV among adults, we will also estimate the treatment effect at 3 and 5 years follow up for the set of secondary outcomes detailed in section 2.2 for which data are available. Key secondary outcomes include: vertical transmission; adult, maternal and pediatric mortality; plasma HIV RNA levels; antiretroviral resistance; AIDS; tuberculosis and opportunistic infections; and, linkage, time to ART initiation, retention in care for HIV-infected subjects. Given a community level estimate for each of these

secondary outcomes, statistical analysis to evaluate the intervention's effect on each of these outcomes will follow the general approach described for evaluation of the primary outcome, based on unadjusted and adjusted comparison of treatment and control communities. In the following subsections we outline the data that will be collected to estimate each secondary outcome.

b. Mortality

Deaths and births within each study community will be ascertained using a combination of data from the CHC, post-CHC tracking, local death registries and partnerships between staff and government sponsored village health teams or their equivalent. We will estimate all-cause mortality among adults, children < 1 year of age (infant mortality), children < 5 years of age (pediatric mortality), and women who are pregnant or within 42 days of termination of pregnancy (pregnancy-related mortality).

c. Mother to Child Transmission of HIV

We will evaluate the effect of the intervention on the proportion of live births still alive and HIV uninfected at two years, among all births and among births to HIV-infected mothers. We focus on the outcome among infants two years after birth to capture the interventions effect on transmission prenatally, during birth, and during breastfeeding. Evaluating HIV-free survival among all births, and not only among HIV-infected mothers will allow us to capture the effect of the intervention on vertical transmission rates due to its effect on decreasing the prevalence of HIV infected mothers as well as any effect due to reducing the probability of a mother who is HIV infected transmitting the virus to her baby.

Data from implementing partners on the HIV status of children born to HIV+ women at health facilities will be assessed throughout the study. In addition, estimation of 2 year HIV free survival rates for each community will be performed based on CHCs, combined with tracking of non-attendees. This will provide us with a birth cohort that is representative of the entire community (and not only of those mothers who engage with antenatal care). Specifically, an infant will enter the cohort when his or her mother is seen at the CHC or tracked and the birth reported.

d. Plasma HIV RNA levels, CD4 Cell Count, and Antiretroviral Resistance

Quantitative HIV-1 RNA PCR testing will be used for HIV RNA level metrics, including geometric and arithmetic mean and median HIV RNA level and proportion with HIV RNA level below the limit of detection among all HIV-infected individuals. In addition, HIV RNA will be measured from all HIV+ members of the household socioeconomic survey, providing annual data from both control and intervention communities.

Drug resistance among HIV-infected individuals will be measured by assaying dried blood spots collected during the CHC, HSE and at tracking for the mutations K103N, M184V, and K65R. We will estimate proportion of treatment naive individuals with each

and with any of these three mutations as a marker of transmitted resistance in each community. As markers of acquired resistance, we will also estimate proportion of HIV infected individuals with resistance mutations among those individuals who initiated treatment.

Finally, point of care CD4 cell count testing at the CHC and among tracked subjects will allow us to estimate CD4 cell count recovery rates annually in the intervention arm and will provide population based data after 3 and 5 years of follow up in both arms.

Importantly, the use of the CHC plus tracking will provide us with estimates of each of these metrics among all HIV-infected individuals, regardless of whether they are retained in care. In addition to comparing these community level metrics between treatment and control communities, we will also assess how these outcomes vary as a function of CD4 at antiretroviral initiation, including estimating these metrics in the subpopulation of subjects who initiate therapy at CD4 >350 cells/uL. Supplemental data on HIV+ patients in care will also be obtained from clinic records.

e. Linkage, Retention, and Time to ART Initiation

Use of the CHC combined with tracking of a random sample of non-returnees (irrespective of HIV status), and linkage of resulting data to clinic records will allow us to generate estimates of linkage and retention rates among all HIV infected individuals. Specifically, we will estimate for each community over time the proportion of newly diagnosed HIV infected individuals who successfully link to care (defined as any visit to clinic), as well as the proportion retained in care (defined as at least two visits in the past 12 months). In addition, we will estimate the average time from first HIV diagnosis to ART initiation for each community.

f. Internally Derived HIV Infections

Viral consensus sequences will be used to estimate phylogenetic relationships and genetic distances between HIV viruses sampled during the study. These data, together with additional reference sequences, will be used to classify incident HIV infections among community cohort members as linked or not linked to previously documented infections among community members [41].

Internally derived incident HIV infection will be defined as an incident HIV infection in a study participant classified, based on sequence analysis, as linked to a virus previously measured from a member of the same community. Externally derived incident HIV infection will be defined as infection with a virus classified as unlinked to a previously measured virus in a member of the same community.

The community specific outcome for this secondary analysis will be the probability of becoming infected over the course of the study by an internally derived virus.

g. Additional Health Outcomes

Measurement of AIDS-defining events, TB, and treatment-associated toxicities and adverse events will include passive surveillance systems and secondary data sources, as described in section 6.3.2 and 7.4, with analytic methods used whenever possible to reduce bias in estimates of the underlying population parameters.

Confirmed active TB cases will be identified using existing registries. HIV status of confirmed cases will be based on a) HIV status as recorded in the registry and b) linkage with SEARCH study based on name and demographic information (following an initial feasibility study).

AIDS-defining events among HIV-infected individuals will be measured by obtaining WHO Stage IV diagnoses recorded in clinic. Other information may be obtained from community health campaigns, tracking or hospital records. Note that measurement at the clinic will rely on linkage and retention of HIV infected patient in care. The resulting outcome data will thus be subject to both selection bias and potentially informative interval censoring under a non-monotone missingness pattern (i.e. patient will be seen at clinic intermittently, some not for long intervals, and detection of these outcomes will only be possible when they are seen).

11.5.2 Supplementary Analyses

a. Analysis of Process Outcomes

For each step of the care cascade (testing update, linkage, and retention) we will report basic descriptive statistics, including unadjusted and adjusted associations between individual level characteristics and retention in the cascade.

b. Effect Modification and Mediation

A modification of the two stage approach described for the primary and secondary outcomes above will also be applied to investigate how individual level characteristics modify the effect of the intervention, and the extent to which the intervention effect is mediated by update of specific intervention components. Secondary analyses may also include pooled individual level analyses.

11.5.3 Economic and Education Evaluations

The objective of our analyses will be to determine the causal effect of early ART initiation on several socio-economic and education outcomes. The outcomes will have a different data structure than the health outcomes described above, with annual longitudinal data being obtained for a sample of baseline CHC participants and baseline non-participants and their households. For the entire sample of household socio-economic (HSE) survey participants and for the baseline CHC and non-CHC samples our analysis will assess whether changes in socio-economic outcomes differ between intervention and comparison communities. The analysis will also examine the relationship between socio-economic status and HIV status of respondents.

For the sample of HIV-positive study participants we will analyze changes in socio-economic status as a function of changes in CD4 cell count and viral load. Analyses will be performed using STATA 11.0 statistical software.

Sample Size

A total of 200 participants and their household will be recruited for the HSE in each of the study communities. This will include 100 HIV-infected individuals and their households and 100 HIV-uninfected individuals and their households. The sample will consist of individuals who did and did not participate in the CHC.

Estimation Strategy

The study objectives will be met by comparing changes in the main outcomes between intervention and comparison communities. The following outcome variables will be studied: adults' on- and off-farm employment; children's on- and off-farm employment (child labor); children's time allocation to schooling and household activities; asset holdings (durable good and livestock); agricultural output; cash and in-kind transfers.

For each outcome, a mixed effects regression model will be estimated in order to determine whether there are significant differences between intervention and control communities over time. Standard errors will be clustered at the community level in regressions that include individual- or household-level observations. A limited set of household-level characteristics will be included as covariates in the model. Differences between intervention and control communities will be examined at year 5 and at each of the follow-up periods during which the HSE is conducted in order to identify short- and long-term effects of ART. Analyses will be conducted separately for baseline CHC participants and non-participants. For children's education and labour supply outcomes, the analyses will test for different effects on young and old children due to variation in the ways in which households adjust time allocation of household members. Effects of ART on the labour supply of adults will also be allowed to vary for men and women. In the models that are estimated, trends in the outcome variable that are not due to ART will be identified by including controls for the month during which the HSE was conducted.

11.5.4 Health Care Costing Evaluations

Costs

Costs will be assessed both from the health care system analytic perspective, and from the patient's perspective. During the five-years of cost data collection, costs will be measured using empirical data as described in section 10.3 above. However, because important health effects of ART and the treatment of other chronic disease extends beyond this period, we will model the consequences of early versus later ART initiation using the best available data on disease progression and the health states and associated medical care costs.

Unit Cost Measures

We will calculate several measures of the cost per programmatic goal achieved, from proximate to distal. The proximate measures will be cost per HIV+ person identified, linked to care, and started on ART. The intermediate measure will be cost per ART-month (person on ART for a month). The distal efficiency measures will focus on surrogate biological markers of ART success: cost per CD4 level recovered and viral load suppressed.

Health Status

We will translate observed and projected health events into a standard metric of disease burden, Disability Adjusted Life Years (DALYs). The calculated DALYs occurring will reflect health benefits (e.g., added years of life from ART) estimated from the morbidity and mortality measured during the study, plus future health effects of HIV incidence measured during the trial, using our published methods to project future health burden of HIV adjusted for ART access.

Cost-Effectiveness

Finally we will estimate incremental cost-effectiveness. This is the net added cost per health outcome, e.g., per DALY averted. Both the numerator and the denominator represent the difference between study arms. Thus, the **numerator** will reflect differences in the cost of ART use and in savings from averted disease. (Both arms have a community testing campaign.). The **denominator** will represent the difference in DALYs due to the clinical benefits of ART, HIV infections averted, and any other observed disease effects. The **ratio** is the ICER (incremental cost-effectiveness ratio), in dollars per DALY averted. If the intervention saves money and improves health (“dominant” in cost-effectiveness parlance), no ICER will be calculated (since there is no cost-health tradeoff); results will be expressed as economic savings and health gains.

Sensitivity Analyses

To estimate the impact of uncertainty in inputs, we will conduct extensive one-way and two-way sensitivity analyses. We will also using Monte Carlo multi-variable simulations to estimate the confidence intervals associated with the base-case incremental cost-effectiveness ratios.

12. DATA COLLECTION AND MONITORING

12.1 Data and Safety Monitoring Plan

The SEARCH project will employ a multi-tiered approach to monitoring the progress of the trial for ethics, safety, efficacy, and futility. Monitoring will take place at the levels of the community, the host country, and study-wide through defined groups and processes (see Table 6).

Table 7: SEARCH Trial Monitoring Information Sources and Responsibilities

Information Tier	Organization Unit	Responsibilities
Community	Community Advisory Boards (CAB)	Experiences and concerns of community members of the study communities and agencies.
Host Country	Local Advisory Boards (LAB)	Ethical and policy issues and developments potentially impacting the conduct and rationale of the trial within the host country.
Science & Ethics	Scientific Advisory Board (SAB)	Scientific and ethical issues and potentially impacting the conduct and rationale of the trial.
Study Data	Data Safety Monitoring Board (DSMB)	Study progress including incidence of significant adverse events, interim efficacy and futility analyses, and issues from community and scientific advisory boards.

Information on the conduct and acceptance of the study will be obtained at the community level through the local Community Advisory Boards who are charged with representing the experiences and concerns of community members of the study communities. Issues and concerns about the conduct and impact of the trial on the community will be communicated in writing from local CAB to study investigators and presented in summaries to the study Data Safety Monitoring Board (DSMB). Likewise, scientific and policy issues and developments potentially impacting the conduct and rationale of the trial at the host country level will be assessed by the study-wide and host-country site Scientific Advisory Boards and communicated in writing to study investigators and presented in summaries to the study DSMB. Finally, study progress including incidence of significant adverse events and interim analyses will be presented to the study DSMB and study statisticians in written form and in person at annual or ad-hoc DSMB meetings.

12.1.1 Data and Safety Monitoring Board

Pursuant to the NIH policy for Data and Safety Monitoring: a Data Safety and Monitoring Board will be convened to provide oversight of the SEARCH trial. The role of the DSMB will be to review implementation and progress of the trial and to review the accumulating data from the study to detect early, significant benefit or harm for communities and persons while the trial is in progress. The DMSB will consist of three main categories of Members: 1) Voting members, 2) Advisors, and 3) Observers. *Voting* Members will consist of 5 individuals, including a Chair, who will be nominated by the protocol Principal Investigators and approved by the Executive Steering Committee for the study. At least one of the voting members will be a national of the host site countries. Voting Members are appointed for the duration of the study and participate in all closed-session meetings and votes. The voting membership will possess expertise in medicine, epidemiology/trials, statistics, and ethics/participant advocacy***.*** *Advisor* members will consist of 3-5 individuals nominated by Voting Members who provide & exchange knowledge, context and advice, and are approved by the Executive Steering Committee for the study. Advisors do not participate in DSMB voting activities. Observer members are board members that support operations and functions of the DSMB and its participant members but do not directly participate in discussions as representatives of areas of expertise or outside agencies. The DSMB will convene to review study progress and safety and may be called into ad hoc sessions as the Board sees fit or at the request of the study Principal Investigator. At the meeting, the study Statistician and Principal Investigator will present summaries of issues and concerns from the local and study-wide advisory boards and the trial progress and safety and efficacy data, including the results of any planned interim analyses, to the DSMB for consideration. Following its meetings, the DSMB will act in an advisory capacity to the investigators to monitor study participant safety and data quality and evaluate progress of the study and present its recommendations in writing to NIH, study sponsors and the study Principal Investigator.

12.1.2 Interim Reports and Study Stopping Guidelines

Early in the first year the DSMB shall meet to review the DSMB Charter and its guiding principles and to familiarize itself with the study protocol, the primary statistical analysis plan, and the trial safety stopping rules. The first DSMB data review will occur after all 32 communities have completed their first community health campaign and subsequent tracking and a period of 60 days has elapsed to allow for referral patient linkage-to-care. Over the course of the trial one scheduled interim analysis will be performed when all 32 communities have completed collecting 3 years of incidence data. Interim analysis of HIV incidence and of all secondary outcomes for which data are available will be completed as described in the statistical plan (Section 11). At the time of the interim analysis a meeting of the DSMB will be convened and the study team will present information on study progress and the results of interim analyses. Should event triggers such as an unanticipated serious adverse event, series of unanticipated adverse events, or significant developments in the field indicate to the DSMB Chair and the Principal Investigator that the DSMB should meet, an ad hoc review will be scheduled as soon as possible.

DSMB reports will contain information on community and advisory board issues and concerns and on study progress and data quality (including community health campaign testing, ART

initiation and distribution) and safety data (serious adverse events and deaths). For stopping guidelines, the protocol team recommends that an exceptional difference (in either direction) in deaths between the study arms could justify early termination of the study. For the purposes of this study, an exceptional difference in death rates among HIV infected persons with a risk ratio of 3 or greater that is statistically significant according to Peto-Haybittle criteria. The protocol team does not recommend stopping the trial for early indications of intervention efficacy without full consideration of the treatment and policy landscape. . A rationale for continuation in the setting of early significant evidence of efficacy is the value in evaluating the magnitude and durability of the intervention over the full study period. Another rationale is the need for complete data on the economic impact of the intervention (work productivity, reduced health care expenditures for serious HIV illnesses) and the beneficial effect on the other health conditions and children's education. On the other hand, the benefits to continuing the study in its current form may be outweighed by the benefits (to control communities, policy makers, the larger region, or the global community) of the making study results public or modifying the study design at year three. Because the scientific, treatment and policy landscape is evolving rapidly, the protocol team feels that the DSMB will be better positioned to weigh these tradeoffs immediately prior to disclosure of the interim analysis. Prior to any presentation of pre-specified study analyses, the DSMB will meet and consider the full scientific, treatment, and policy landscape at the time of the interim analysis. In consultation with advisors, the DSMB will then issue a formal recommendation regarding criteria for stopping, modifying, or un-blinding the study based on results of the interim analysis. On balance the protocol team believes continuing to evaluate the full effect of the intervention could be ethical and warranted, but the understanding that the board should consider all important contexts in its recommendations.

12.2 Baseline Household Community Level Census

Household Census data will be collected by teams using hand-held computers (tablets). Prior to conducting the census, the census questionnaire will be programmed into the hand-held computers. Programming will include range checks, structure checks and internal consistency checks. Before leaving the household, the completed questionnaire will be checked for mistakes and completeness, ensuring each household has a unique identifier. Data from these devices will be transferred daily via a secure electronic transfer to our data center facility in Kampala and stored on a secure server.

Each household location will be mapped using a hand-held GPS receiver. Readings will be taken from the door of the household, if possible, or from a point that is most representative of the household. The GPS coordinates for each household will also be recorded in the tablet computer at the time of administering the census questionnaire. GPS data will be synchronized from the GPS to a Microsoft Access database daily and then transferred via a secure electronic transfer to the data center facility in Kampala and stored on a secure server.

A digital biometric identifier based on an electronic fingerprint of each household member will be captured in an electronic database on the hand-held computer and linked to the household member name. A portable fingerprint reader will be connected to the tablet computer via a USB port and the biometric identifier will be saved into the electronic database on the tablet. The

database will be transferred daily via a secure electronic transfer to the data center facility in Kampala and stored on a secure server.

12.3 Community Health Campaign

12.3.1 Welcome Station

The first stop for participants during most Community Health Campaign will be the Welcome Station. There will be an electronic database of all the biometric identifiers collected during the Census Survey available at the Welcome Station. Staff members will verify participant's fingerprint biometric identifier against the database to ensure they have a biometric identifier in the system. If a participant did not have a biometric identifier taken during the Census Survey, a biometric identifier based on his or her digital fingerprint will be taken at the Welcome Station and added to the database. Once the participant's biometric identifier is verified, the participant will be given a bracelet with a unique identifier on it. The unique identifier will be added to the biometric identifier database and linked to the participant's biometric identifier. The participant will then be tracked through the Community Health Campaign with the unique identifier on the bracelet.

12.3.2 Health and Socioeconomic Interview Station

During most campaigns, health and socio-economic data will be collected by trained staff members electronically using tablet computers. Prior to conducting the Health and Socioeconomic Interview, the interview will be programmed into the tablet computers. Programming will include range checks, structure checks and internal consistency checks. The unique identifier on the participant's bracelet will be used to link the participant to their interview data. Data from the tablet computers will be transferred daily via a secure electronic transfer to our data center facility in Kampala and stored on a secure server.

12.3.3 Other CHC Stations

During the campaign, all information recorded at each of the stations will be recorded in Log Books by staff members. The unique identifier on the participant's bracelet will be used to link the participant to their data in the Log Books. Afterwards, the Log Books will be entered directly into an electronic database. Data Integrity checks will be written into the database to limit the entry of incorrect data and ensure entry of data into required fields. All data will be double entered to verify accuracy of entry. The database will be transferred regularly via a secure electronic transfer to the data center facility in Kampala and stored on a secure server.

12.3.4 Tracking CHC Non-Participants

Apart from the verbal autopsy questionnaire, data collected during evaluation of non-participants will be collected using hand-held computers (tablets). Prior to conducting the evaluation, the questionnaire will be programmed into the hand-held computers. Programming will include range checks, structure checks and internal consistency checks. Data from these devices will be transferred daily via a secure electronic transfer to our data center facility in Kampala and stored on a secure server.

12.3.5 Morbidity/Disease Surveillance

Study staff will regularly collect information available from routine encounters at local health centers and, where needed, hospitals within the community. This information will be available in the clinic's standard visit forms and recorded by staff for data entry.

Afterwards, the surveillance forms will be entered directly into an electronic database. Data Integrity checks will be written into the database to limit the entry of incorrect data and ensure entry of data into required fields. All data will be double entered to verify accuracy of entry. The database will be transferred regularly via a secure electronic transfer to the data center facility in Kampala and stored on a secure server.

12.4 ART Intervention

All data will be obtained from encounter forms at local health centers and recorded onto standardized case record forms by study staff. Afterwards, the forms will be entered directly into an electronic database. Data Integrity checks will be written into the database to limit the entry of incorrect data and ensure entry of data into required fields. All data will be double entered to verify accuracy of entry. The database will be transferred regularly via a secure electronic transfer to the data center facility in Kampala and stored on a secure server.

12.5 Grade 3 and 4 Adverse Event and Serious Adverse Event Monitoring

Grade 3 and 4 adverse events (AEs) and serious adverse events (SAEs) will be monitored in all sentinel cohorts. We will utilize the DAIDS Toxicity Table for Adults and Children grading scale (Appendix A) for reported symptoms and laboratory monitoring. The sentinel cohorts will be composed of persons in the intervention arm with CD4 >350 cells. These individuals will be matched on CD4 to individuals not receiving ART and evaluated semi-annually through chart review supplemented by tracking visits.

The following definitions for serious or unexpected adverse events will be followed:

A Serious Adverse Event (SAE) is any AE that results in any of the following outcomes:

- Death,
- Life-threatening adverse experience,
- Inpatient hospitalization or prolongation of existing hospitalization,
- Persistent or significant disability/incapacity,
- Congenital anomaly/birth defect, or cancer, or
- Any other experience that suggests a significant hazard, contraindication, side effect or precaution that may require medical or surgical intervention to prevent one of the outcomes listed above,
- Event occurring in a gene therapy study
- Event that changes the risk/benefit ratio of the study.

An Unexpected Adverse Event is defined as being unexpected if the event exceeds the nature, severity, or frequency described in the protocol, consent form and investigator brochure (when applicable). An unexpected AE also includes any AE that meets any of the following criteria:

- Results in subject withdrawal from study participation,
- Due to an overdose of study medication, or
- Due to a deviation from the study protocol

Adverse events in sentinel sites will be reported to individual IRBs according to the table below:

Table 8: Adverse Events

Institution	Type of Adverse Events	When to Report
**UCSF-CHR**	• External [off-site] adverse event that UCSF PI determines changes the study risks or benefits, OR necessitates modification to the CHR-approved consent document(s) and/or the CHR-approved application/protocol	• Within 10-working days of PI's awareness
**SOMREC**	• All Serious or Unexpected events irrespective of relationship	• Fatal or life-threatening events within 3 working days of awareness<br>• All other SAEs within 7 calendar days
**NDA**	• All serious and Unexpected events irrespective of relationship	• Within 7-calendar days of awareness
**KEMRI SERU**	• All Serious or Unexpected events irrespective of relationship	• Study-related events within 24 hours of awareness<br>• Unrelated events within 10 working days of awareness
**Gilead Sciences**	• Definitely, Probably, or Possibly related to Gilead-supplied Truvada **AND** Serious or Unexpected	• Within 15-calendar days of awareness

12.6 Household Socio-Economic Survey

Household Socio-Economic Survey data will be collected by teams using hand-held computers (tablets). Prior to conducting the survey, the questionnaire will be programmed into the hand-held computers. Programming will include range checks, structure checks and internal consistency checks. Before leaving the household, the completed questionnaire will be checked for mistakes and completeness, ensuring each household has a unique identifier. Data from these devices will be transferred daily via a secure electronic transfer to our data center facility in Kampala and stored on a secure server.

12.7 Health Care Costing Evaluations

Each health facility will be identified using a code. Health facility time in motion data will be recorded on paper and transferred to a database by study staff. Cost data will be collected in Microsoft Excel and imported to a database (Microsoft Access or FileMaker) for storage and manipulation. Although there is no confidential patient information in the cost data, it will be integrated with standard secure methods used for other data in the study. The data files will be regularly backed up on secure servers

12.8 Data Security and Integrity

In order to ensure data security and integrity, the following measures will be implemented:

- All members of the study team will be educated in the study protocol prior to the onset of the study.
- Detailed Standard Operating Procedures (SOPs) will be written for all project activities and be provided to relevant team members.
- Team members will be thoroughly trained on the SOP's.
- Where applicable, team members will receive additional training on the use of GPS devices.
- Where applicable, team members will receive additional training on the use of tablet computers.
- All data transcribed from paper will be double data entered or verified.
- All electronic data will be backed up on a daily basis.
- All data will be transferred to the main Data Center in Kampala to the secure server. This sever is backed up on a daily basis and a monthly backup is stored off-site.
- All computers, including the tablets, will be password protected.
- All computers, including tablets, will be locked in a secure room each night.
- All Log Books and CRF's will be locked in a secure room each night.

13. HUMAN SUBJECTS

13.1 Ethical Considerations

13.1.1 ART Intervention

In this study, ART will be initiated at CD4+ thresholds higher than the national guidelines defining standard of care. We must therefore consider the potential benefits and risks of ART at higher CD4 counts to ensure that the key intervention of this study (ART initiation) meets the strictest ethical guidelines. We submit that initiation of ART at all CD4 thresholds meets and exceeds ethical standards by several criteria:

1) ART is routinely initiated in all HIV-positive patients throughout many countries.

2) ART initiation for all CD4 thresholds is now recommended by many professional HIV medicine societies. For example, ARTs are now formally recommended by the United States Department of Health and Human Services for all patients.

3) Accumulating evidence indicates that there may be substantial clinical benefit to initiating ART as soon as a person is HIV infected. This benefit may accrue from reductions in immune activation and systemic inflammation, as well as limitation of the size of the latent reservoir of HIV. These pathophysiologic discoveries have been partially responsible for the changes in clinical practice guidelines detailed above.

4) The additional amount of time a patient will take ART if initiated at any CD4 threshold vs. country-specific guidelines is relatively small. Accumulated data on the speed of HIV progression (i.e., the time taken for CD4 to decline to 350 cells/uL from the time of infection) indicates that this may take, on average, 2-3 years in most patients. This time is even shorter for countries when policies change to starting at a threshold below 500 cells/uL. This study, therefore, will bridge this length of treatment administration, after which point participants will be allowed to continue ART provided by the local government's country-specific guidelines.

13.2 Institutional Review Board (IRB) and Informed Consent

13.2.1 Obtaining Consent

This protocol, all procedures and consent forms, and any subsequent modifications must be reviewed and approved by the IRBs of all the participating institutions in the U.S., Uganda and Kenya. This includes the UCSF Committee on Human Research (CHR), the Makerere University School of Medicine - Research and Ethics Committee (SOM-REC), the Uganda National Council of Science and Technology (UNCST), the Uganda National Drug Authority (NDA), and the Kenya Medical Research Institute (KEMRI) Scientific and Ethics Review Unit.

The Kenya Pharmacy and Poisons Board (PPB) will be notified of any modifications to the protocol and consent forms as well.

All consent forms will be translated into the local language and back-translated into English to ensure correct use of language. Consent forms will be read aloud to participants or their parents by trained staff. The informed consent will describe the purpose of the study, all the procedures involved, and the risks and benefits of participation. Interviewers will ask participants or their parents/guardians to summarize the study and explain the reasons why they want to participate. Either a signature or a thumbprint (for those who cannot read) will be acceptable to confirm informed consent for participation in the study, in the case of written consent forms.

Verbal consent will be obtained from adults to participate in the Baseline Household Community Level Census and, for those not available during the Census, at the initial Community Health Campaign. Consent will obtained for adults and their children, by reading the approved consent script and documenting agreement by recording their fingerprint biometric on portable, password-protected computers. Verbal consent will also be obtained for adults participating in the Targeted HIV Testing/Key Population Testing procedures. Written consent will be obtained from adults to participate in the Household Socio-Economic Survey, focus groups, in-depth qualitative interviews and surveys on stigma and new infections, and for the ART Intervention activities. In addition, children 13 years or older will provide consent to participate in ART Intervention, with parental co-signature; written assent will be obtained from children 8 to 12 years old, with parental co-signature; and parental written consent will be obtained for children less than 8 years old. In addition, a letter of commitment will be obtained from community leaders for their site's participation in the study. Consent to take photographs or film adults and their children during Community Health Campaign activities, and for de-identified photographs for dermatologic evaluations, will also be obtained from participants. See Appendix B for further details.

14. PUBLICATION OF RESEARCH FINDINGS

The findings from this study may be published in a medical journal. No individual identities will be used in any reports or publications resulting from the study. The researchers will publish results of the study in accordance with NIAID, UCSF, UNCST, KEMRI and Makerere University guidelines.

REFERENCES

1. UNAIDS Report on the global AIDS epidemic 2010. 2010, UNAIDS.

2. Hogan, M.C., et al., Maternal mortality for 181 countries, 1980-2008: a systematic analysis of progress towards Millennium Development Goal 5. Lancet, 2010. 375(9726): p. 1609-23.

3. Beegle, K., J. De Weerdt, and S. Dercon, The intergenerational impact of the African orphans crisis: a cohort study from an HIV/AIDS affected area. Int J Epidemiol, 2009. 38(2): p. 561-8.

4. Kublin, J.G., et al., Effect of Plasmodium falciparum malaria on concentration of HIV-1-RNA in the blood of adults in rural Malawi: a prospective cohort study. Lancet, 2005. 365(9455): p. 233-40.

5. Havlir, D.V., et al., Opportunities and challenges for HIV care in overlapping HIV and TB epidemics. Jama, 2008. 300(4): p. 423-30.

6. Lawn, S.D. and A.D. Harries, Reducing tuberculosis-associated early mortality in antiretroviral treatment programmes in sub-Saharan Africa. Aids, 2011. 25(12): p. 1554-5; author reply 1556.

7. Cohen, M.S., et al., Prevention of HIV-1 infection with early antiretroviral therapy. N Engl J Med, 2011. 365(6): p. 493-505.

8. Granich, R.M., et al., Universal voluntary HIV testing with immediate antiretroviral therapy as a strategy for elimination of HIV transmission: a mathematical model. Lancet, 2009. 373(9657): p. 48-57.

9. Wagner, B. and S. Blower, Costs of eliminating HIV in South Africa have been underestimated. Lancet, 2010. 376(9745): p. 953-4.

10. Wagner, B.G., J.S. Kahn, and S. Blower, Should we try to eliminate HIV epidemics by using a 'Test and Treat' strategy? Aids, 2010. 24(5): p. 775-6.

11. Dieffenbach, C.W. and A.S. Fauci, Thirty years of HIV and AIDS: future challenges and opportunities. Ann Intern Med, 2011. 154(11): p. 766-71.

12. Weber, J., R. Tatoud, and S. Fidler, Postexposure prophylaxis, preexposure prophylaxis or universal test and treat: the strategic use of antiretroviral drugs to prevent HIV acquisition and transmission. Aids, 2010. 24 Suppl 4: p. S27-39.

13. Dodd, P.J., G.P. Garnett, and T.B. Hallett, Examining the promise of HIV elimination by 'test and treat' in hyperendemic settings. Aids, 2010. 24(5): p. 729-35.

14. Wawer, M.J., et al., Circumcision in HIV-infected men and its effect on HIV transmission to female partners in Rakai, Uganda: a randomised controlled trial. Lancet, 2009. 374(9685): p. 229-37.

15. Rosen, S. and M.P. Fox, Retention in HIV care between testing and treatment in sub-Saharan Africa: a systematic review. PLoS Med, 2011. 8(7): p. e1001056.

16. Middelkoop, K., et al., Antiretroviral therapy and TB notification rates in a high HIV prevalence South African community. J Acquir Immune Defic Syndr, 2011. 56(3): p. 263-9.

17. Zachariah, R., et al., Reduced tuberculosis case notification associated with scaling up antiretroviral treatment in rural Malawi. Int J Tuberc Lung Dis, 2011. 15(7): p. 933-7.

18. Flateau, C., G. Le Loup, and G. Pialoux, Consequences of HIV infection on malaria and therapeutic implications: a systematic review. Lancet Infect Dis, 2011. 11(7): p. 541-56.

19. Fowler, M.G., et al., Perinatal HIV and its prevention: progress toward an HIV-free generation. Clin Perinatol, 2010. 37(4): p. 699-719, vii.

20. Schouten, E.J., et al., Prevention of mother-to-child transmission of HIV and the health-related Millennium Development Goals: time for a public health approach. Lancet, 2011. 378(9787): p. 282-4.

21. Donnell, D., et al., Heterosexual HIV-1 transmission after initiation of antiretroviral therapy: a prospective cohort analysis. Lancet, 2010. 375(9731): p. 2092-8.

22. Thirumurthy H, Goldstein M, and Graff Zivin J, The economic impact of AIDS treatment: labor supply in western Kenya. Journal of Human Resources, 2008. 43(June 20): p. 511-552.

23. Habyarimana, J., B. Mbakile, and C. Pop-Eleches, The Impact of HIV/AIDS and ARV Treatment on Worker Absenteeism: Implications for African Firms J. Human Resources, 2010. 45(September 21): p. 809-839.

24. Rosen, S., et al., Economic outcomes of patients receiving antiretroviral therapy for HIV/AIDS in South Africa are sustained through three years on treatment. PLoS One, 2010. 5(9): p. e12731.

25. Thirumurthy, H., et al., Two-year impacts on employment and income among adults receiving antiretroviral therapy in Tamil Nadu, India: a cohort study. Aids, 2011. 25(2): p. 239-46.

26. Graff Zivin, J., H. Thirumurthy, and M. Goldstein, AIDS treatment and intrahousehold resource allocation: Children's nutrition and schooling in Kenya. Journal of Public Economics, 2009. 93(7-8): p. 1008-1015.

27. Integrated National Guidelines on Antiretroviral Therapy, Prevention of Mother to Child Transmission of HIV and on Infant & Young Child Feeding. 2011, Ministry of Health, Republic of Uganda.

28. Guidelines for Antiretroviral Therapy in Kenya, 4th edition 2011. 2011, Ministry of Medical Sciences, Republic of Kenya.

29. Muennig, P.A. and K. Khan, Cost-effectiveness of vaccination versus treatment of influenza in healthy adolescents and adults. Clin Infect Dis, 2001. 33(11): p. 1879-85.

30. Marseille, E.A., et al., Case management to improve adherence for HIV-infected patients receiving antiretroviral therapy in Ethiopia: a micro-costing study. Cost Eff Resour Alloc, 2011. 9: p. 18.

31. Hayes, R.J. and L.H. Moulton, Cluster Randomized Trials 2009: Chapman and Hall.

32. Pagel, C., et al., Intracluster correlation coefficients and coefficients of variation for perinatal outcomes from five cluster-randomized controlled trials in low and middle-income countries; results and methodological implications. Trials, 2011. 12: p. 151.

33. Chingono, A., et al., Balancing science and community concerns in resource-limited settings: Project Accept in rural Zimbabwe. Clin Trials, 2008. 5(3): p. 273-6.

34. Laan, M.J.v.d. and S. Rose, Targeted Learning: Causal Inference for Observational and Experimental Data. 2011, Berlin Heidelberg New York: Springer.

35. van der Laan, M., L. Balzer, and M. Petersen, Adaptive Matching in Randomized trials and Observational Studies. Journal of Statistical Research, 2012 46(2): p. 113-156.

36. Balzer, L., M.L. Petersen, and M.J. Van der Laan, Adaptive pair-matching in the SEARCH trial and estimation of the intervention effect. U.C. Berkeley Division of Biostatistics Working Paper Series, 2014(320).

37. Hayes, R.J. and S. Bennett, Simple sample size calculation for cluster-randomized trials. Int J Epidemiol, 1999. Apr; 28(2): p. 319-26.

38. Guwatudde, D., et al., Relatively Low HIV Infection Rates in Rural Uganda, but with High Potential for a Rise: A Cohort Study in Kayunga District, Uganda. PloS One, 2009. 4(1): p. e4145.

39. Shafer, L.A., et al., HIV prevalence and incidence are no longer falling in southwest Uganda: evidence from a rural population cohort 1989-2005. AIDS, 2008. 22: p. 1641-49.

40. Gray, R.H., et al., Male circumcision for HIV prevention in men in Rakai, Uganda: a randomised trial. Lancet, 2007. 369: p. 657-66.

41. Campbell, M.S., et al., Viral linkage in HIV-1 seroconverters and their partners in an HIV-1 prevention clinical trial. PLoS One, 2011. 6(3): p. e16986.

Appendix A Guidelines for Adverse Event Grading – DAIDS Toxicity Table for Adults and Children

Selected portion of the of Division of AIDS [DAIDS] Table for Grading the Severity of Adult and Pediatric Adverse Events, version Dec. 2004.

CLINICAL				
**PARAMETER**	**GRADE 1<br>MILD**	**GRADE 2<br>MODERATE**	**GRADE 3<br>SEVERE**	**GRADE 4<br>POTENTIALLY LIFE-THREATENING**
**ESTIMATING SEVERITY GRADE**				
Clinical adverse event NOT identified elsewhere in this DAIDS AE grading table	Symptoms causing no or minimal interference with usual social & functional activities	Symptoms causing greater than minimal interference with usual social & functional activities	Symptoms causing inability to perform usual social & functional activities	Symptoms causing inability to perform basic self-care functions OR Medical or operative intervention indicated to prevent permanent impairment, persistent disability, or death
**SYSTEMIC**				
Acute systemic allergic reaction	Localized urticaria (wheals) with no medical intervention indicated	Localized urticaria with medical intervention indicated OR Mild angioedema with no medical intervention indicated	Generalized urticaria OR Angioedema with medical intervention indicated OR Symptomatic mild bronchospasm	Acute anaphylaxis OR Life-threatening bronchospasm OR laryngeal edema
Chills	Symptoms causing no or minimal interference with usual social & functional activities	Symptoms causing greater than minimal interference with usual social & functional activities	Symptoms causing inability to perform usual social & functional activities	NA
Fatigue<br>Malaise	Symptoms causing no or minimal interference with usual social & functional activities	Symptoms causing greater than minimal interference with usual social & functional activities	Symptoms causing inability to perform usual social & functional activities	Incapacitating fatigue/ malaise symptoms causing inability to perform basic self-care functions
Fever (nonaxillary)	37.7 – 38.6°C	38.7 – 39.3°C	39.4 – 40.5°C	> 40.5°C
Pain (indicate body site)<br>DO NOT use for pain due to injection (See Injection Site Reactions: Injection site pain)<br>See also Headache, Arthralgia, and Myalgia	Pain causing no or minimal interference with usual social & functional activities	Pain causing greater than minimal interference with usual social & functional activities	Pain causing inability to perform usual social & functional activities	Disabling pain causing inability to perform basic self-care functions OR Hospitalization (other than emergency room visit) indicated

CLINICAL				
**PARAMETER**	**GRADE 1 MILD**	**GRADE 2 MODERATE**	**GRADE 3 SEVERE**	**GRADE 4 POTENTIALLY LIFE-THREATENING**
Unintentional weight loss	NA	5 – 9% loss in body weight from baseline	10 – 19% loss in body weight from baseline	≥ 20% loss in body weight from baseline OR Aggressive intervention indicated [e.g., tube feeding or total parenteral nutrition (TPN)]
**SKIN – DERMATOLOGICAL**				
Cutaneous reaction – rash	Localized macular rash	Diffuse macular, maculopapular, or morbilliform rash OR Target lesions	Diffuse macular, maculopapular, or morbilliform rash with vesicles or limited number of bullae OR Superficial ulcerations of mucous membrane limited to one site	Extensive or generalized bullous lesions OR Stevens-Johnson syndrome OR Ulceration of mucous membrane involving two or more distinct mucosal sites OR Toxic epidermal necrolysis (TEN)
Pruritis (itching – no skin lesions)<br>(See also Injection Site Reactions: Pruritis associated with injection)	Itching causing no or minimal interference with usual social & functional activities	Itching causing greater than minimal interference with usual social & functional activities	Itching causing inability to perform usual social & functional activities	NA
**GASTROINTESTINAL**				
Anorexia	Loss of appetite without decreased oral intake	Loss of appetite associated with decreased oral intake without significant weight loss	Loss of appetite associated with significant weight loss	Life-threatening consequences OR Aggressive intervention indicated [e.g., tube feeding or total parenteral nutrition (TPN)]
Diarrhea	Transient or intermittent episodes of unformed stools OR Increase of ≤ 3 stools over baseline per 24-hour period	Persistent episodes of unformed to watery stools OR Increase of 4 – 6 stools over baseline per 24-hour period	Bloody diarrhea OR Increase of ≥ 7 stools per 24-hour period OR IV fluid replacement indicated	Life-threatening consequences (e.g., hypotensive shock)
Nausea	Transient (< 24 hours) or intermittent nausea with no or minimal interference with oral intake	Persistent nausea resulting in decreased oral intake for 24 – 48 hours	Persistent nausea resulting in minimal oral intake for > 48 hours OR Aggressive rehydration indicated (e.g., IV fluids)	Life-threatening consequences (e.g., hypotensive shock)
Vomiting	Transient or intermittent vomiting with no or minimal interference with oral intake	Frequent episodes of vomiting with no or mild dehydration	Persistent vomiting resulting in orthostatic hypotension OR Aggressive rehydration indicated (e.g., IV fluids)	Life-threatening consequences (e.g., hypotensive shock)

CLINICAL				
**PARAMETER**	**GRADE 1 MILD**	**GRADE 2 MODERATE**	**GRADE 3 SEVERE**	**GRADE 4 POTENTIALLY LIFE-THREATENING**
**RESPIRATORY**				
Dyspnea or respiratory distress	Dyspnea on exertion with no or minimal interference with usual social & functional activities	Dyspnea on exertion causing greater than minimal interference with usual social & functional activities	Dyspnea at rest causing inability to perform usual social & functional activities	Respiratory failure with ventilatory support indicated

LABORATORY				
**PARAMETER**	**GRADE 1 MILD**	**GRADE 2 MODERATE**	**GRADE 3 SEVERE**	**GRADE 4 POTENTIALLY LIFE-THREATENING**
**HEMATOLOGY**	***Standard International Units are listed in italics***			
Absolute neutrophil count (ANC)	1,000 – 1,300/mm^3<br>*1.000 x 10^9 – 1.300 x 10^9/L*	750 – 999/mm^3<br>*0.750 x 10^9 – 0.999 x 10^9/L*	500 – 749/mm^3<br>*0.500 x 10^9 – 0.749 x 10^9/L*	< 500/mm^3<br>*< 0.500 x 10^9/L*
Hemoglobin (Hgb)<br>**Adult and Pediatric ≥ 57 days**<br>(HIV POSITIVE ONLY)	8.5 – 10.0 g/dL<br>*1.32 – 1.55 mmol/L*	7.5 – 8.4 g/dL<br>*1.16 – 1.31 mmol/L*	6.50 – 7.4 g/dL<br>*1.01 – 1.15 mmol/L*	< 6.5 g/dL<br>*< 1.01 mmol/L*
Platelets, decreased	100,000 – 124,999/mm^3<br>*100.000 x 10^9 – 124.999 x 10^9/L*	50,000 – 99,999/mm^3<br>*50.000 x 10^9 – 99.999 x 10^9/L*	25,000 – 49,999/mm^3<br>*25.000 x 10^9 – 49.999 x 10^9/L*	< 25,000/mm^3<br>*< 25.000 x 10^9/L*
WBC, decreased	2,000 – 2,500/mm^3<br>*2.000 x 10^9 – 2.500 x 10^9/L*	1,500 – 1,999/mm^3<br>*1.500 x 10^9 – 1.999 x 10^9/L*	1,000 – 1,499/mm^3<br>*1.000 x 10^9 – 1.499 x 10^9/L*	< 1,000/mm^3<br>*< 1.000 x 10^9/L*
**CHEMISTRIES**	***Standard International Units are listed in italics***			
ALT (SGPT)	1.25 – 2.5 x ULN	2.6 – 5.0 x ULN	5.1 – 10.0 x ULN	> 10.0 x ULN
AST (SGOT)	1.25 – 2.5 x ULN	2.6 – 5.0 x ULN	5.1 – 10.0 x ULN	> 10.0 x ULN
Bilirubin (Total)	1.1 – 1.5 x ULN	1.6 – 2.5 x ULN	2.6 – 5.0 x ULN	> 5.0 x ULN
Creatinine	1.1 – 1.3 x ULN[†]	1.4 – 1.8 x ULN[†]	1.9 – 3.4 x ULN[†]	≥ 3.5 x ULN[†]
Potassium, serum, high	5.6 – 6.0 mEq/L<br>*5.6 – 6.0 mmol/L*	6.1 – 6.5 mEq/L<br>*6.1 – 6.5 mmol/L*	6.6 – 7.0 mEq/L<br>*6.6 – 7.0 mmol/L*	> 7.0 mEq/L<br>*> 7.0 mmol/L*
Potassium, serum, low	3.0 – 3.4 mEq/L<br>*3.0 – 3.4 mmol/L*	2.5 – 2.9 mEq/L<br>*2.5 – 2.9 mmol/L*	2.0 – 2.4 mEq/L<br>*2.0 – 2.4 mmol/L*	< 2.0 mEq/L<br>*< 2.0 mmol/L*
Sodium, serum, high	146 – 150 mEq/L<br>*146 – 150 mmol/L*	151 – 154 mEq/L<br>*151 – 154 mmol/L*	155 – 159 mEq/L<br>*155 – 159 mmol/L*	≥ 160 mEq/L<br>*≥ 160 mmol/L*

LABORATORY				
**PARAMETER**	**GRADE 1 MILD**	**GRADE 2 MODERATE**	**GRADE 3 SEVERE**	**GRADE 4 POTENTIALLY LIFE-THREATENING**
Sodium, serum, low	130 – 135 mEq/L<br>*130 – 135 mmol/L*	125 – 129 mEq/L<br>*125 – 129 mmol/L*	121 – 124 mEq/L<br>*121 – 124 mmol/L*	≤ 120 mEq/L<br>*≤ 120 mmol/L*
Glucose, serum, high				
Nonfasting	116 – 160 mg/dL<br>*6.44 – 8.88 mmol/L*	161 – 250 mg/dL<br>*8.89 – 13.88 mmol/L*	251 – 500 mg/dL<br>*13.89 – 27.75 mmol/L*	> 500 mg/dL<br>*> 27.75 mmol/L*
Fasting	110 – 125 mg/dL<br>*6.11 – 6.94 mmol/L*	126 – 250 mg/dL<br>*6.95 – 13.88 mmol/L*	251 – 500 mg/dL<br>*13.89 – 27.75 mmol/L*	> 500 mg/dL<br>*> 27.75 mmol/L*
Glucose, serum, low	55 – 64 mg/dL<br>*3.05 – 3.55 mmol/L*	40 – 54 mg/dL<br>*2.22 – 3.06 mmol/L*	30 – 39 mg/dL<br>*1.67 – 2.23 mmol/L*	< 30 mg/dL<br>*< 1.67 mmol/L*

Appendix B Table of Study and Consent and Commitment Procedures

Study component	No. of participants in Mbarara district, Uganda	No. of participants in Tororo district, Uganda	No. of participants in Kenya	Procedures	Timeline	Consent or commitment process
Community Leader	10	10	12	Meet with local community leaders representing each of the 32 selected communities	Prior to the first Household Census and Community Health Campaign in each selected community	Obtain letter of commitment from leader of each selected community
Baseline Household Community Level Census	All individuals in selected communities	All individuals in selected communities	All individuals in selected communities	Census of all community members to collect identifier and location information	Prior to first Community Health Campaign in each selected community; also at Campaign for those not previously consented	Verbal consent of head of household for themselves and other household members; fingerprint biometric will be recorded as consent documentation
Community Health Campaign	All individuals in selected communities	All individuals in selected communities	All individuals in selected communities	• Tests and measurements including rapid HIV testing, malaria testing on children and other health evaluations<br>• Education and referral to local health services<br>• Distribution of anti-malaria medications and vitamin A in children, and male condoms	Prior to first Community Health Campaign in each selected community; also at Campaign for those not previously consented	Verbal adult consent for themselves and their children to participate in Campaign; fingerprint biometric will be recorded as consent documentation
Targeted/Key Population Testing	Any individuals in selected key population groups	Any individuals in selected key population groups	Any individuals in selected key population groups	• Rapid HIV testing<br>• CD4 testing, if HIV-positive<br>• An optional survey on background and sexual history	Any time after initial Community Health Campaign in each selected community	Verbal consent of participating adult, age ≥15 years
Household Socio-Economic Survey	200 x 10 communities, 100 HIV+ & 100 HIV- (n = 2,000)	200 x 10 communities, 100 HIV+ & 100 HIV- (n = 2,000)	200 x 12 communities, 100 HIV+ & 100 HIV- (n = 2,400)	A survey to collect demographic, health and education information of household members	2-4 weeks after each Campaign	Written consent, adults, conducted at Community Health Campaign
Photography/filming at Community Health Campaigns	Any participants at selected Community Health Campaigns	Any participants at selected Community Health Campaigns	Any participants at selected Community Health Campaigns	Taking photographs or filming participants at selected Campaigns	At selected Community Health Campaigns	Written consent of adults and their children at Campaigns
Photography for dermatologic	Eligible participants in selected	Eligible participants in selected	Eligible participants in selected	De-identified photography of skin conditions at	At selected Community Health Campaigns	Verbal consent of adults at Campaigns

evaluations	communities	communities	communities	selected Campaigns		
ART Intervention	All HIV+ individuals who do not meet in-country treatment guidelines	All HIV+ individuals who do not meet in-country treatment guidelines	All HIV+ individuals who do not meet in-country treatment guidelines	Distribution of ART and routine testing in a streamlined model of care	144 weeks from ART initiation for each participant	• Written consent, adults<br>• Parental written consent, children <8 years<br>• Written assent, children 8-12 years, with parental written consent<br>• Written consent, children ≥13 years, parental co-sign
Qualitative evaluation	a.) 2-4 Community leaders in 2 communities<br><br>b.) 24-30 Community Health Campaign participants in communities conducting CHCs<br><br>c.) 10 randomly selected service providers in 2 communities (5 per community)<br><br>d.) Sub-sample of Household Socio-Economic Survey in 2 communities: 14 x 2 communities,<br>9 HIV+ & 5 HIV- (n = 28)	a.) 2-4 Community leaders in 2 communities<br><br>b.) 24-30 Community Health Campaign participants in communities conducting CHCs<br><br>c.) 10 randomly selected service providers in 2 communities (5 per community)<br><br>d.) Sub-sample of Household Socio-Economic Survey in 2 communities: 14 x 2 communities,<br>9 HIV+ & 5 HIV- (n = 28)	a.) 22-4 Community leaders in 4 communities<br><br>b.) 24-30 Community Health Campaign participants in communities conducting CHCs<br><br>c.) 10 randomly selected service providers in 4 communities (5 per community)<br><br>d.) Sub-sample of Household Socio-Economic Survey in 4 communities: 14 x 4 communities,<br>9 HIV+ & 5 HIV- (n = 56)	a.) In-depth semi-structured interviews<br><br>b.) Focus group discussions (1 for women, 1 for men, and 1 mixed gender group per community; 8-10 individuals in each group)<br><br>c.) In-depth semi-structured interviews<br><br>d.) In-depth semi-structured interviews	a.) 1-2 weeks after each Community Health Campaign<br><br>b.) Within 1-2 weeks after each Community Health Campaign<br><br>c.) 2-4 weeks after each Household Socio-Economic Survey<br><br>d.) 2-4 weeks after each Household Socio-Economic Survey	For all research activities related to qualitative evaluation of SEARCH: Written consent, adults
New infection qualitative cohort	A subset of participants enrolled in the ART Intervention arm in selected communities	A subset of participants enrolled in the ART Intervention arm in selected communities	A subset of participants enrolled in the ART Intervention arm in selected communities	A survey on experiences related to new HIV infection	Within 6 months of enrollment in ART Delivery arm	Written consent, adults
Stigma questionnaire	A random subset of HIV- and HIV+ community members	A random subset of HIV- and HIV+ community members	A random subset of HIV- and HIV+ community members	A questionnaire on stigma issues related to HIV	Any time at CHCs, participant homes or work places, or at health clinics in selected communities	Written consent, adults

Appendix C Uganda Antiretroviral Therapy Guidelines, in place until 2014

Selected portion of The Integrated National Guidelines on Antiretroviral Therapy, Prevention of Mother to Child Transmission of HIV and Infant & Young Child Feeding, Ministry of Health, Uganda.

ART Initiation in Adults

The current MoH guidelines are as summarized in a table bellow

Including HIV positive Partner in a discordant relationship irrespective of CD4 count

Category	Recommendation	First line treatment	
All children <15years	Start ART irrespective of CD4 count	<3yrs	ABC/3TC/NVP
		3-9.9yrs	ABC/3TC/EFV
		10-14.9	<35kg ABC/3TC/EFV
			>35KG TDF/3TC/EFV
		15>	TDF/3TC/EFV
Pregnant women	Start ART irrespective of CD4	OptionB+<br>TDF/3TC/EFV	
Adults	With CD4 less ≤ than 500<br>Stage 3and 4, TB , Hep B, HepC , TB co-infection irrespective of CD4	TDF/3TC/EFV	
Most at risk populations (fisher folks, truckers, CSWs)	Start ART irrespective of CD4	TDF/3TC/EFV	

Eligibility criteria for initiating art in infants and children

Three parameters guide the decision making process for initiation of ART in infants and children; these are the age, immunological status and WHO clinical Staging. However ART can also be started in children under 18 months of age presumptively (*as will be described in the next section.*)

Appendix D Kenya Antiretroviral Therapy Guidelines

Selected portion of the Guidelines on Use of Antiretroviral Drugs for Treating and Preventing HIV Infection, Rapid Advice, June 2014.

When to Initiate Antiretroviral Therapy in Adolescents and Adults

The following are the recommendations/indications for initiating ART in HIV-infected adolescents and adults with documented HIV infection.

Table D.1: Criteria for Initiation of ART in Adults and Adolescents

**Population**	**Recommendations**
When to start ART in adolescents ≥15 years and adults	• All HIV-infected adolescents and adults with CD4 count <u><</u>500 cells/mm3 irrespective of WHO stage<br>• All HIV-infected pregnant women irrespective of CD4 count, WHO stage or gestation age<br>• All HIV-infected breastfeeding women irrespective of CD4 count, WHO stage<br>• All HIV-infected spouses and sexual partners in sero-discordant relationships irrespective of their WHO stage or CD4 cell count<br>• All HIV-infected adolescents and adults with WHO stage 3 and 4 disease irrespective of CD4 count<br>• All Hepatitis B Virus/HIV co-infected persons irrespective of CD4 count<br>• All TB/HIV co-infected persons irrespective of CD4 count

When to start antiretroviral therapy in children less than 15 years

The following are the recommendations/indications for initiating ART in HIV-infected children less than 15 years.

Table D.2: Criteria for Initiation of ART in Children less than 15 years

**Population**	**Recommendations**
When to start ART in children less than 15 years	• ART should be initiated in all HIV-infected children **aged 10 years and below**, regardless of WHO stage or CD4 count/%.<br>• ART should be initiated in all HIV infected children **above 10 years of age** with CD4 cell count ≤500 cells/mm3, regardless of WHO stage<br>• All HIV-infected children above 10 years with WHO stage 3 and 4 disease, Hepatitis B Virus/HIV, TB/HIV co-infection should be initiated on ART irrespective of CD4 count<br>• In circumstances where DNA PCR testing is not readily available ART should be initiated in any child younger than 18 months of age who meets criteria for presumptive diagnosis of severe HIV disease, **confirmatory DNA PCR testing should be done as soon as possible**

<u>Use of antiretroviral drugs for treating HIV-positive pregnant and breastfeeding women</u>

The Ministry of Health recommends immediate initiation of life-long ART for pregnant and breastfeeding women upon HIV diagnosis. The use of ART in pregnant and breastfeeding women markedly reduces the transmission of HIV infection from mother to child. Continuing ART for life for the mother provides additional benefit of keeping mothers healthy and alive, and may also offer benefits for preventing sexual transmission of HIV in sero-discordant relationships.

Appendix E Sample Pediatric ART Dosing Chart

		3 - 5.9 kg	6 - 9.9 kg	10 - 13.9 kg	14 - 19.9 kg	20 - 20.9 kg	25 - 25.9 kg
Tablets/Capsules	EFV 200 - 100 - 50mg	NR	NR	200mg daily	300mg daily	300mg daily	400mg daily
	ABC 300mg	NR	NR	NR	0.5 BID	1 AM/0.5 PM	1 BID
	ABC 60mg	1 BID	1.5 BID	2 BID	2.5 BID	3 BID	Use adult
	3TC 150mg	NR	NR	NR	0.5 BID	1 AM/0.5 PM	1 BID
	LPV/r 100/25mg	NR	NR	2 AM/1 PM	2 BID	2 BID	3 BID
	LPV/r 200/50mg	NR	NR	NR	1 BID	1 BID	2 AM/1PM
	AZT 300mg	NR	NR	NR	0.5 BID	1 AM/0.5 PM	1 BID
	NVP 200mg	NR	NR	NR	1 AM/0.5 PM	1 AM/0.5 PM	1 BID
	NVP 50mg	1 BID	1.5 BID	2 BID	2.5 BID	3 BID	Use adult
Oral Solutions	ABC 20mg/ml	3ml BID	4ml BID	6ml BID	NR	NR	NR
	3TC 10mg/ml	3ml BID	4ML BID	6ml BID	NR	NR	NR
	LPV/r 80/20 mg/ml	3-3.9kg: 1ml BID<br>4-5.9kg: 1.5ml BID	1.5ml BID	2ml BID	2.5ml BID	3ml BID	3.5ml BID
	AZT 10mg/ml	6ml BID	9ml BID	12ml BID	NR	NR	NR
	NVP 10mg/ml	5ml BID	8ml BID	10ml BID	NR	NR	NR
