# Supplementary material for: Characteristics of HIV seroconverters in the setting of universal test and treat: Results from the SEARCH trial in rural Uganda and Kenya
Source: PLoS One. 2021 Feb 5;16(2):e0243167. doi: 10.1371/journal.pone.0243167 (PMC7864429; doi:10.1371/journal.pone.0243167)
Supplement: S3 File — (DOCX) [file pone.0243167.s003.docx]

**Appendix 2: Additional analytic details**

First, we provide descriptive statistics of members of the HIV Incidence Cohort who seroconverted over the 3-year trial. These statistics help us to understand the characteristics of the seroconverters and are reported overall and by gender and region. Next, we calculate HIV incidence rates (seroconversions per 100 person-years) with exact Poisson confidence intervals for each gender and within gender for each of the following subgroups: age group (<25 years or 25+ years), marital status (single, married, divorced or separated, widowed), occupation (formal, high-risk informal, low-risk informal, jobless, other), wealth index quintiles, contraceptive use (yes, no, declined to answer), alcohol use (yes, no, declined to answer), mobility (1+ month away from community at baseline or not), self-reported HIV testing prior to baseline, and the location of the baseline HIV test (health fair or home-based). For persons not acquiring HIV, person-time-at-risk is calculated as the difference between the final HIV test (at year 3) and the initial HIV test (at baseline). For persons acquiring HIV, person-time-at-risk is calculated as half the difference between the final HIV test (at year 3) and the initial HIV test (at baseline). Primary analyses will exclude members of the HIV incidence cohort who move out of the study community.

Then, to quantify gender-specific predictors of HIV seroconversion, we stratify on gender and within men and women separately, estimate variable importance measures(1-3) for each of the following predictors of interest: age group (<25 years or 25+ years), marital status (single, married, divorced or separated, widowed), occupation (formal, high-risk informal, low-risk informal, jobless, other), wealth index quintiles, contraceptive use (yes, no, declined to answer), alcohol use (yes, no, declined to answer), mobility (1+ month away from community at baseline or not), self-reported HIV testing prior to baseline, and the location of the baseline HIV test (health fair or home-based). These measures capture how much information a given predictor provides, after adjusting for the other predictors. Although not predictors of interest in the primary analysis, variable importance analyses additionally adjust for region and intervention arm. Variable importance measures are estimated on the relative scale (i.e. relative risks) with targeted maximum likelihood estimation (TMLE) that additionally controls for incomplete follow-up.

TMLE is a semiparametric efficient estimation method that incorporates machine learning to minimize bias due to model misspecification and to avoid over-fitting(1). To estimate a variable importance measure with TMLE, we implement the following steps for each predictor of interest. First, using Super Learner, an ensemble machine learning algorithm, we estimate the outcome regression (here, the conditional probability of HIV infection, given all the covariates) and the propensity score (here, the conditional probability having the predictor of interest, given the remaining ones). Second, we update the initial estimate of the outcome regression, using the corresponding estimate of the propensity score. Finally, we average the updated estimates and obtain inference with through the influence function. For each predictor of interest, this approach provides flexibility in the functional form of the outcome regression and propensity score, while avoiding reliance on correctly specified parametric models. Incomplete follow-up is incorporated by additional estimation of the probability of having a missing HIV outcome, given adjustment variables. In secondary analyses, we report unadjusted analyses as well as adjusted analyses pooling over gender and restricting to the intervention arm, where universal test-and-treat (UTT) was implemented from baseline.

Finally, based on qualitative interviews of participants who seroconverted, we will create descriptive tables of the self-reported suspected source and mode of HIV infection, overall and by gender and region.

All analyses were pre-specified in the Statistical Analysis Plan for the SEARCH trial(4).

**REFERENCES**

1. Van der Laan MJ, Rose S. Targeted learning: causal inference for observational and experimental data: Springer Science & Business Media; 2011.

2. Rose S. Targeted Learning for Variable Importance. Handbook of Big Data. 2016;411.

3. Lendle SD, Schwab J, Petersen ML, van der Laan MJ. ltmle: an R package implementing targeted minimum loss-based estimation for longitudinal data. Journal of Statistical Software. 2017;81(1):1-21.

4. Balzer LB, Havlir DV, Schwab J, Van Der Laan MJ, Petersen ML. Statistical Analysis Plan for SEARCH Phase I: Health Outcomes among Adults. arXiv preprint arXiv:180803231. 2018.
