# Supplementary material for: Characteristics of HIV seroconverters in the setting of universal test and treat: Results from the SEARCH trial in rural Uganda and Kenya
Source: PLoS One. 2021 Feb 5;16(2):e0243167. doi: 10.1371/journal.pone.0243167 (PMC7864429; doi:10.1371/journal.pone.0243167)
Supplement: S1 Table — (DOCX) [file pone.0243167.s004.docx]

**S1 Table. Descriptive characteristics in percent (numerator/denominator) of 117,114 members of the HIV incidence cohort, overall and by gender.**

| **Seroconversion Predictor** | **Level** | **All (117114)** | **Male (52286)** | **Female (64828)** |
| --- | --- | --- | --- | --- |
| Age | 25+ years old | 63% (74334/117114) | 61% (31881/52286) | 65% (42453/64828) |
|  | <25 years old | 37% (42780/117114) | 39% (20405/52286) | 35% (22375/64828) |
| Marital Status | Divorced or separated | 3% (3732/116801) | 2% (956/52109) | 4% (2776/64692) |
|  | Single | 28% (33025/116801) | 38% (19961/52109) | 20% (13064/64692) |
|  | Married | 61% (70808/116801) | 59% (30540/52109) | 62% (40268/64692) |
|  | Widowed | 8% (9236/116801) | 1% (652/52109) | 13% (8584/64692) |
| Occupation | Formal | 23% (26771/116800) | 29% (15255/52108) | 18% (11516/64692) |
|  | High-risk Informal | 4% (4997/116800) | 7% (3570/52108) | 2% (1427/64692) |
|  | Low-risk Informal | 63% (73627/116800) | 53% (27696/52108) | 71% (45931/64692) |
|  | Jobless | 5% (6049/116800) | 4% (2150/52108) | 6% (3899/64692) |
|  | Other | 5% (5356/116800) | 7% (3437/52108) | 3% (1919/64692) |
| Wealth index | First, least wealth | 16% (18802/116929) | 15% (7859/52201) | 17% (10943/64728) |
|  | Second | 18% (20815/116929) | 17% (9051/52201) | 18% (11764/64728) |
|  | Third | 20% (23681/116929) | 20% (10397/52201) | 21% (13284/64728) |
|  | Fourth | 22% (25709/116929) | 22% (11689/52201) | 22% (14020/64728) |
|  | Fifth, most wealth | 24% (27922/116929) | 25% (13205/52201) | 23% (14717/64728) |
| Contraceptive use | No | 59% (69005/117114) | 63% (32841/52286) | 56% (36164/64828) |
|  | Yes | 21% (24854/117114) | 21% (11144/52286) | 21% (13710/64828) |
|  | Declined to respond | 20% (23255/117114) | 16% (8301/52286) | 23% (14954/64828) |
| Alcohol use | No | 79% (92071/117114) | 70% (36709/52286) | 85% (55362/64828) |
|  | Yes | 14% (16701/117114) | 23% (12260/52286) | 7% (4441/64828) |
|  | Declined to respond | 7% (8342/117114) | 6% (3317/52286) | 8% (5025/64828) |
| Mobile | No | 90% (105777/117114) | 88% (46131/52286) | 92% (59646/64828) |
|  | Yes | 10% (11337/117114) | 12% (6155/52286) | 8% (5182/64828) |
| Prior HIV test | No | 46% (53231/116981) | 52% (27144/52237) | 40% (26087/64744) |
|  | Yes | 54% (63750/116981) | 48% (25093/52237) | 60% (38657/64744) |
| Baseline testing | Home-based | 20% (22969/117114) | 25% (13016/52286) | 15% (9953/64828) |
|  | Health fair | 80% (94145/117114) | 75% (39270/52286) | 85% (54875/64828) |
