# Supplementary material for: Characteristics of HIV seroconverters in the setting of universal test and treat: Results from the SEARCH trial in rural Uganda and Kenya
Source: PLoS One. 2021 Feb 5;16(2):e0243167. doi: 10.1371/journal.pone.0243167 (PMC7864429; doi:10.1371/journal.pone.0243167)
Supplement: S2 Table — (DOCX) [file pone.0243167.s005.docx]

S2 Table. Descriptive characteristics in percent (numerator/denominator) of 704 seroconverters identified in the SEARCH test-and-treat trial in 32 rural Uganda and Kenyan communities, overall and by region.

| **Characteristic** |  | **All (704)** | **W Uganda (265)** | **E Uganda (109)** | **Kenya (330)** |
| --- | --- | --- | --- | --- | --- |
| Sex | Male | 37% (261/704) | 43% (115/265) | 37% (40/109) | 32% (106/330) |
|  | Female | 63% (443/704) | 57% (150/265) | 63% (69/109) | 68% (224/330) |
| Age | 15-24 years | 37% (263/704) | 43% (114/265) | 17% (19/109) | 39% (130/330) |
|  | 25-34 years | 34% (236/704) | 32% (84/265) | 37% (40/109) | 34% (112/330) |
|  | 35-44 years | 18% (126/704) | 17% (45/265) | 25% (27/109) | 16% (54/330) |
|  | 45-54 years | 7% (46/704) | 6% (15/265) | 9% (10/109) | 6% (21/330) |
|  | 55+ years | 5% (33/704) | 3% (7/265) | 12% (13/109) | 4% (13/330) |
| Marital Status | Single | 25% (174/703) | 33% (88/265) | 13% (14/108) | 22% (72/330) |
|  | Married | 62% (434/703) | 50% (133/265) | 63% (68/108) | 71% (233/330) |
|  | Widowed | 6% (45/703) | 7% (19/265) | 6% (7/108) | 6% (19/330) |
|  | Divorced or separated | 7% (50/703) | 9% (25/265) | 18% (19/108) | 2% (6/330) |
| Among married | Polygamous marriage | 22% (97/434) | 13% (17/133) | 28% (19/68) | 26% (61/233) |
| Occupation | Farmer | 50% (350/703) | 54% (143/265) | 75% (81/108) | 38% (126/330) |
|  | Fishing/Fishmonger | 7% (48/703) | 0% (1/265) | 0% (0/108) | 14% (47/330) |
|  | Student | 11% (77/703) | 12% (33/265) | 5% (5/108) | 12% (39/330) |
|  | Hotel/Restaurant | 1% (10/703) | 1% (2/265) | 4% (4/108) | 1% (4/330) |
|  | Transport | 2% (15/703) | 2% (5/265) | 1% (1/108) | 3% (9/330) |
| Wealth index | First, least wealth | 21% (145/702) | 31% (81/264) | 22% (24/109) | 12% (40/329) |
|  | Second | 18% (127/702) | 25% (67/264) | 16% (17/109) | 13% (43/329) |
|  | Third | 20% (142/702) | 23% (61/264) | 17% (18/109) | 19% (63/329) |
|  | Fourth | 20% (142/702) | 13% (34/264) | 23% (25/109) | 25% (83/329) |
|  | Fifth, most wealth | 21% (146/702) | 8% (21/264) | 23% (25/109) | 30% (100/329) |
| Contraception | Report use | 31% (199/632) | 25% (61/242) | 22% (21/96) | 40% (117/294) |
| Alcohol | Report use | 19% (123/631) | 26% (70/265) | 32% (35/109) | 7% (18/257) |
| Mobility | 1+ month away | 12% (84/704) | 13% (34/265) | 17% (19/109) | 9% (31/330) |
| Testing | Report prior test | 68% (477/704) | 60% (160/265) | 42% (46/109) | 82% (271/330) |
|  | Test at heath fair | 74% (518/704) | 75% (199/265) | 84% (92/109) | 69% (227/330) |
| Relation to household head | Self | 38% (268/703) | 39% (104/264) | 46% (50/109) | 35% (114/330) |
|  | Spouse | 29% (202/703) | 21% (56/264) | 28% (31/109) | 35% (115/330) |
|  | Child | 24% (167/703) | 28% (73/264) | 17% (19/109) | 23% (75/330) |
|  | Parent | 24% (167/703) | 28% (73/264) | 17% (19/109) | 23% (75/330) |
| Partner | Discordant partner | 0% (2/703) | 0% (1/264) | 1% (1/109) | 0% (0/330) |
|  | Partner did not test | 18% (85/467) | 11% (17/159) | 14% (11/80) | 25% (57/228) |
