# Supplementary material for: Characteristics of HIV seroconverters in the setting of universal test and treat: Results from the SEARCH trial in rural Uganda and Kenya
Source: PLoS One. 2021 Feb 5;16(2):e0243167. doi: 10.1371/journal.pone.0243167 (PMC7864429; doi:10.1371/journal.pone.0243167)
Supplement: S3 Table — (DOCX) [file pone.0243167.s006.docx]

**S3 Table. Adjusted relative risk (95% confidence intervals) for HIV seroconversion by gender. Analyses conducted with targeted maximum likelihood, controlling for incomplete follow-up and also for region and intervention arm. Reference categories are age 25+ years, separated or divorced, formal occupation, lowest wealth quintile, no contraceptive use, no alcohol use, non-mobile, no prior HIV test reported, and home-based testing.**

|  | Men | Women |
| --- | --- | --- |
| Age <25 years | 1.22 (0.69-2.17) | 1.91 (1.27-2.9) |
| Single | 0.5 (0.25-1.01) | 0.78 (0.53-1.15) |
| Married | 0.37 (0.21-0.65) | 0.33 (0.22-0.49) |
| Widowed | 1.11 (0.53-2.32) | 0.53 (0.31-0.92) |
| High-risk informal job | 3.06 (0.95-9.84) | 2.58 (0.92-7.2) |
| Low-risk informal job | 2.44 (0.86-6.94) | 2.81 (1.08-7.32) |
| Jobless | 2.3 (0.67-7.89) | 2.33 (0.82-6.62) |
| Other job | 3.22 (1.14-9.07) | 1.8 (0.59-5.5) |
| 2nd wealth quintile | 1.24 (0.74-2.07) | 0.73 (0.49-1.08) |
| 3rd wealth quintile | 0.93 (0.59-1.45) | 0.78 (0.57-1.06) |
| 4th wealth quintile | 1.15 (0.76-1.76) | 0.51 (0.39-0.68) |
| Highest wealth quintile | 0.75 (0.42-1.31) | 0.60 (0.43-0.84) |
| Contraceptive use | 0.95 (0.67-1.34) | 1.62 (1.24-2.11) |
| Contraceptive declined resp. | 0.44 (0.2-0.95) | 0.76 (0.56-1.02) |
| Alcohol use | 1.76 (0.77-4.06) | 2.07 (1.34-3.22) |
| Alcohol declined resp. | 1.60 (1.28-2) | 0.98 (0.82-1.16) |
| Mobile | 1.68 (1.09-2.6) | 1.49 (1.04-2.11) |
| Prior HIV test | 1.32 (0.97-1.8) | 1.34 (1.06-1.7) |
| Tested at health fair | 0.41 (0.31-0.53) | 0.9 (0.68-1.2) |
