# Supplementary figures and images for: Characteristics of HIV seroconverters in the setting of universal test and treat: Results from the SEARCH trial in rural Uganda and Kenya
Source: PLoS One. 2021 Feb 5;16(2):e0243167. doi: 10.1371/journal.pone.0243167 (PMC7864429; doi:10.1371/journal.pone.0243167)

**S1 Fig. Unadjusted relative risks (95% confidence intervals) for HIV seroconversion by gender.**

**
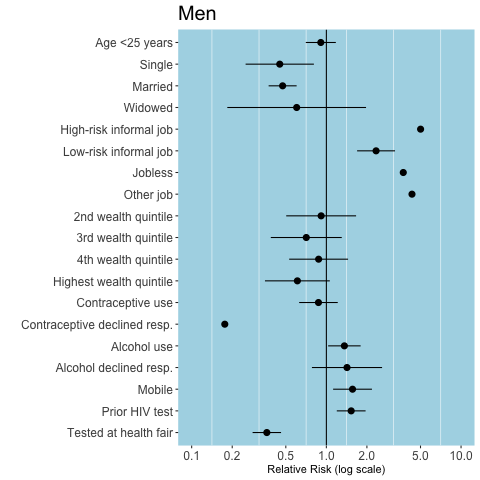

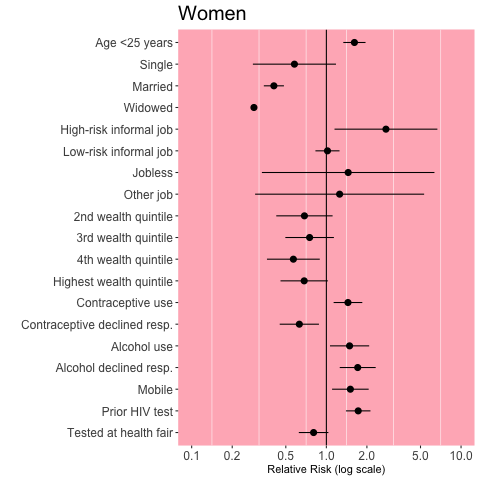
**

Supplement: S1 Fig — (DOCX) [file pone.0243167.s007.docx]
