# Supplementary material for: Characteristics of HIV seroconverters in the setting of universal test and treat: Results from the SEARCH trial in rural Uganda and Kenya
Source: PLoS One. 2021 Feb 5;16(2):e0243167. doi: 10.1371/journal.pone.0243167 (PMC7864429; doi:10.1371/journal.pone.0243167)
Supplement: S2 Fig — (DOCX) [file pone.0243167.s008.docx]

S2 Fig. Adjusted risk ratios for HIV seroconversion pooling over gender. Analyses conducted among members of the HIV incidence cohort using targeted maximum likelihood estimation, controlling for incomplete follow-up and additionally adjusting for region and randomization arm. Reference categories were female, age 25+ years, separated or divorced, formal occupation, lowest wealth quintile, no contraceptive use, no alcohol use, non-mobile, no prior HIV test reported, and home-based testing

**
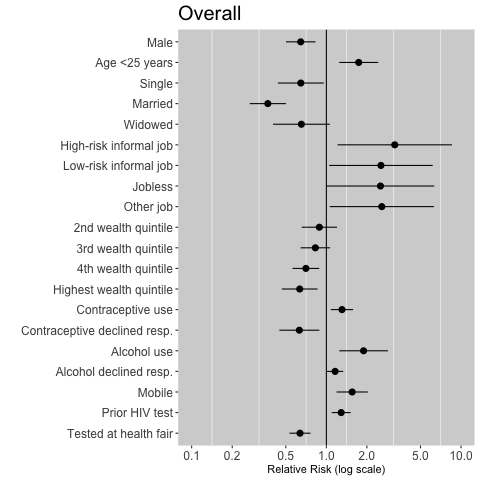
**
